# Supplementary material for: Temperature-dependent jumonji demethylase modulates flowering time by targeting H3K36me2/3 in Brassica rapa
Source: Nat Commun. 2024 Jun 28;15:5470. doi: 10.1038/s41467-024-49721-z (PMC11211497; doi:10.1038/s41467-024-49721-z)
Supplement: Supplementary file 1 — Supplementary Information [file 41467_2024_49721_MOESM1_ESM.pdf]

Supplementary Figure 1. Genetic structures of 210 *Brassica rapa* varieties

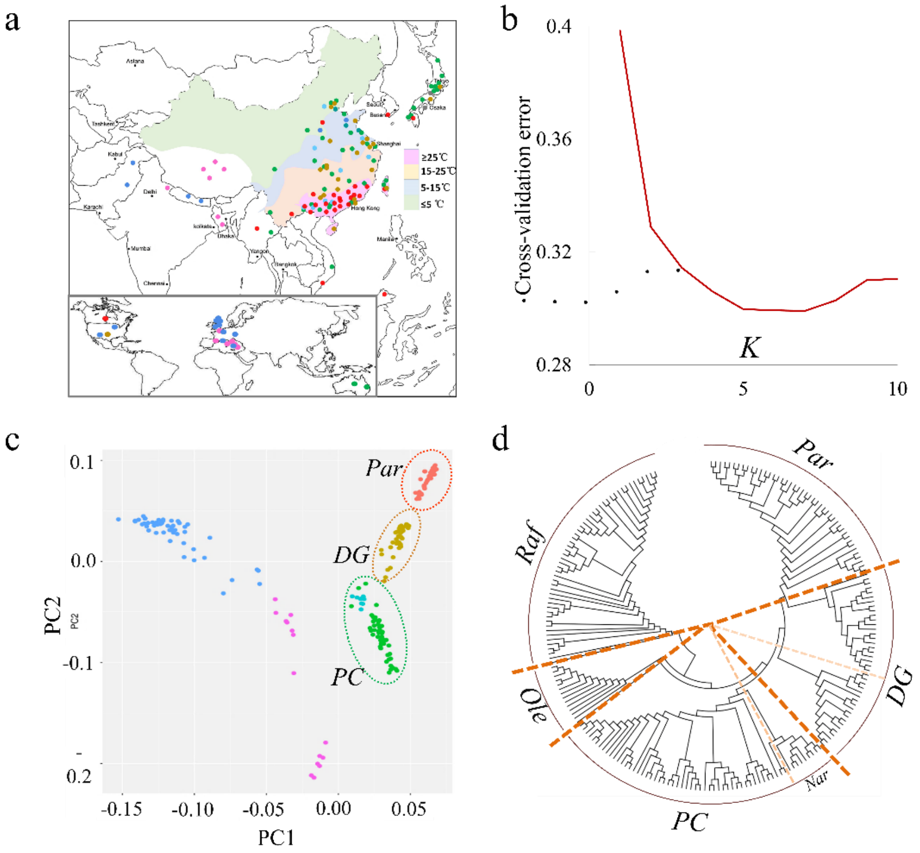

(a) Geographical origins of the 210 lines of different *Brassica rapa* morphotypes. Different colors show regions with different yearly average maximum temperatures. *Par* represents *B. rapa subsp. chinensis var. parachinensis* (marked with red dots), *DG* represents *B. rapa subsp. chinensis var.* Dark-Green (brown dots), *PC* represents *B. rapa subsp. chinensis* (pak choi) (green dots), *Nar* represents *B. rapa subsp. chinensis var. narinosa* (light green dots), *Ole* represents *B. rapa subsp. Oleifera* (rosy dots), and *Raf* represents *B. rapa subsp. rapifera* (blue dots).

(b) Cross-validation at different K levels, with K ranging from 1 to 10. K-fold Cross Validation in the R program was used to estimate the best K value.K = 7 clusters maximized the marginal likelihood and decreasing the number of clusters by one or two, respectively, did not substantially change the observed clustering pattern. For K = 5, *Ole* was clearly separated from *Raf*; For K = 6, *Nar* was divided from *PC*; For K = 7, *DG* was further divided into two clusters, subsp. *chine*nsis *HY* and subsp. *chinensis NB* (Figure 1c; Supplementary Figure 1b). K = 5 explained the population structure best and maximized the marginal likelihood. We selected K=5 for the following analysis. Most of the morphologically distinct crops long recognized as subspecies were largely resolved as distinct clusters in our Structure analyses, which was consistent with the empirical classification.

(c) Principal component analysis (PCA) of diversity in the *B. rapa* collection using the first two principal components. *Par* represents *B. rapa subsp. chinensis var. parachinensis* (marked with red dots), *DG* represents *B. rapa subsp. chinensis var*. Dark-Green (brown dots), *PC* represents *B. rapa subsp. chinensis* (pak choi) (green dots).

(d) A maximum likelihood (ML) tree of all the 210 *B. rapa* accessions inferred from single nucleotide polymorphisms (SNPs) at four-fold-degenerate sites. The phylogenetic tree was constructed using IQ-TREE.

Supplementary Figure 2. The gene flow (migration rate) pattern and population divergence (pairwise F_{ST}) values were evaluated among *PC*, *DG*, and *Par*.

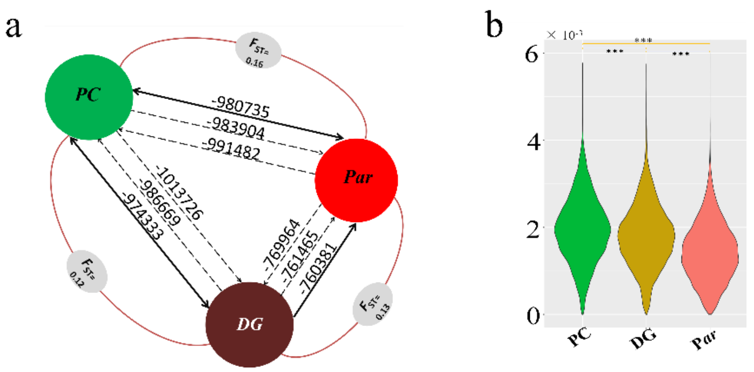

(a) The gene flow (migration rate) pattern and population divergence (pairwise F_{ST}) values were evaluated among *PC*, *DG*, and *Par*. Hypothetical evolutionary relationships among *PC*, *DG*, and *Par* are indicated by gene flow. All of the probable evolutionary patterns (dashed arrows) among the *PC*, *DG,* and *Par* groups are summarized, and the proven evolutionary patterns are shown as solid dark lines. The numbers on the lines of arrows represent the marginal likelihoods of each probable evolutionary pattern among the *PC*, *DG,* and *Par* groups. High values of marginal likelihood suggest a high possibility of the corresponding pattern, and arrows point in the direction of the inferred gene flow. For F_{ST} analysis, The largest population divergence was found between the *Par*/*PC* comparison (F_{ST} = 0.160), following by the *Par*/*DG* (0.131) and *DG*/*PC* (0.118) comparisons, respectively.

(b) Nucleotide diversity (π) within each group. the π values progressively reduced from *PC* (2.04×10^{-3}) to *DG* (1.89×10^{-3}), and then to *Par* (1.79×10^{-3}).

Supplementary Figure 3. Images of of *PC*, *DG* and *Par*.

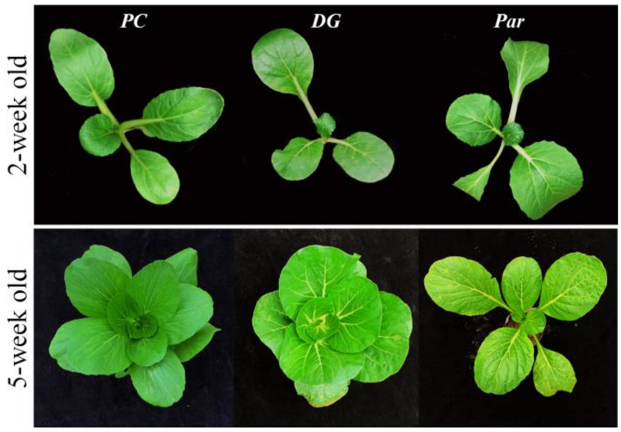

The three subspecies resemble each other at the seedling stage (the upper panel); while at adult juvenile stage, *PC* and *DG* share similar plant architecture, and *DG* and *Par* have similar the leaf shapes and color (the lower panel). Photographs of plants grown under natural field conditions in Beijing for 2- and 5-week were shown. *Par* represents *B. rapa* subsp. *chinensis* var. *parachinensis*, *DG* represents *B. rapa* subsp. *chinensis* var. *Dark-Green*, *PC* represents *B. rapa* subsp. *chinensis* (pak choi).

Supplementary Figure 4. *Par* showed higher thermotolerance comparing with that of *PC* and *DG*.

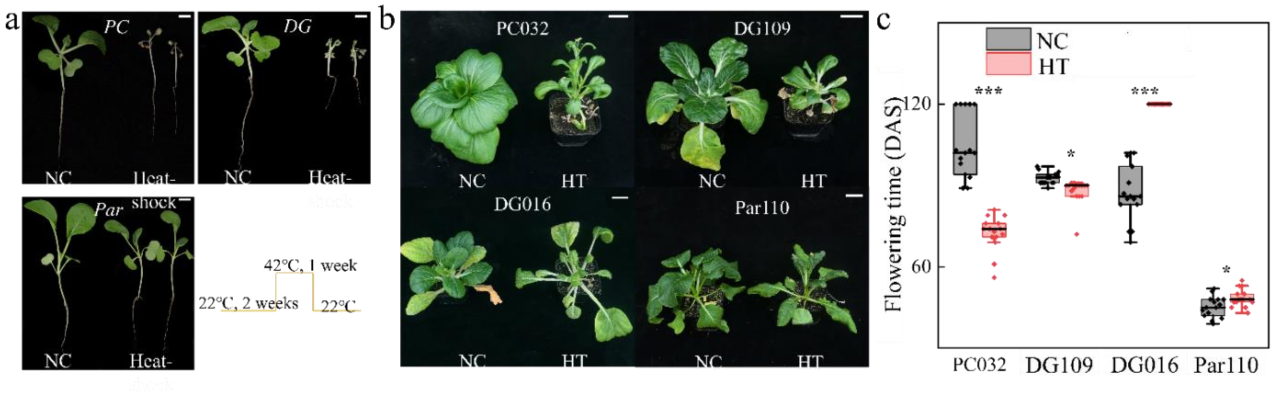

(a) For heat shock treatment, 14-day seedlings grown under normal conditions (NC) were moved to NC and heat-shock conditions (16/8 h day/night, 42°C/42° C), respectively, for another one week. After the high temperature treatment, the plants were recovered at 22°C for 5 days. At the end of recovery, photographs were taken. Scale bar = 2 cm. (b) For high temperature treatment, PC032, a *BrJMJ18^{PC}*-carrying *PC* line, DG109, a *BrJMJ18^{PC}*-carrying *DG* line, DG016, a *BrJMJ18^{Par}*-carrying *DG* line, and Par110, a *BrJMJ18^{Par}*-carrying *Par* line were randomly selected from our germplasm collection. 4-week-old seedlings grown under NC were moved to NC and high temperature (16/8 h day/night, 29°C/29°C, HT) conditions, respectively until flowering. DG106 under HT condition did not flower within the observe window of 120 days. Scale bar = 5 cm. (c) Flowering time of plants shown in (b). Flowering time of DG106 under HT condition was set to 120 days. Data are means ± SD, n = 15. The box encompasses two middle quartiles, with central line showing median. Whiskers extend to the furthest data point within 1.5 times the interquartile range. Asterisks indicate significant differences between NC and HT, two-tailed Student's t-test (*p < 0.05, **p < 0.01, ***p < 0.001). PC032, *p*= 1.58×10^{-08}; DG109, *p*= 0.0014; DG016, *p*= 1.18×10^{-09}; Par110, *p*=0.039. Source data are provided as a Source Data file.

Supplementary Figure 5. Geographical locations and temperature characteristics of cities Zhangjiakou (ZJK) and Beijing (BJ).

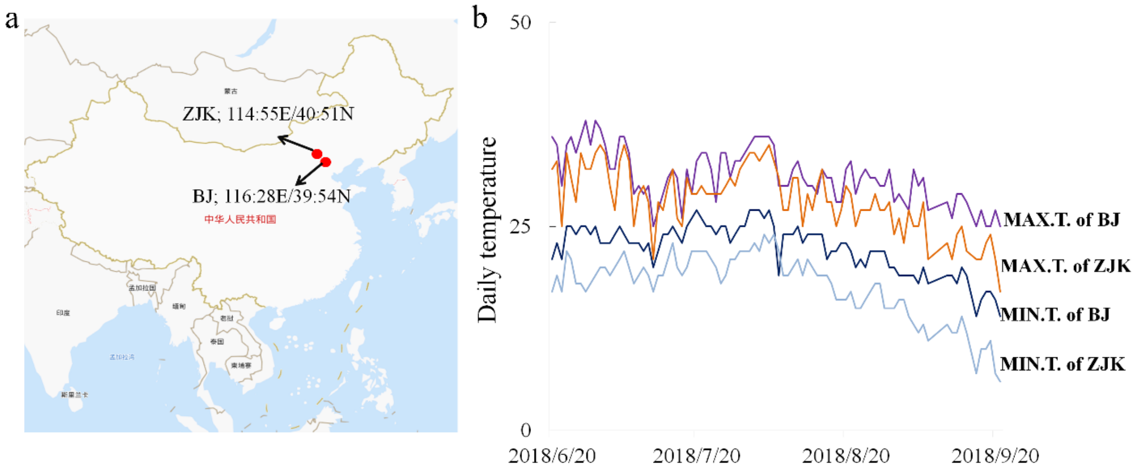

ZJK is 125 miles northwest of BJ (a); both cities have very similar climate characteristics, except for the temperature: The daily temperature of BJ is an average of approximately 5 ºC higher than that of ZJK from the 20th of June to the 20th of September, annually (b). MAX.T., daily maximum temperatures; MIN.T., daily minimum temperatures.

Supplementary Figure 6. Candidate genes that are specifically involved in the domestication of *Par*.

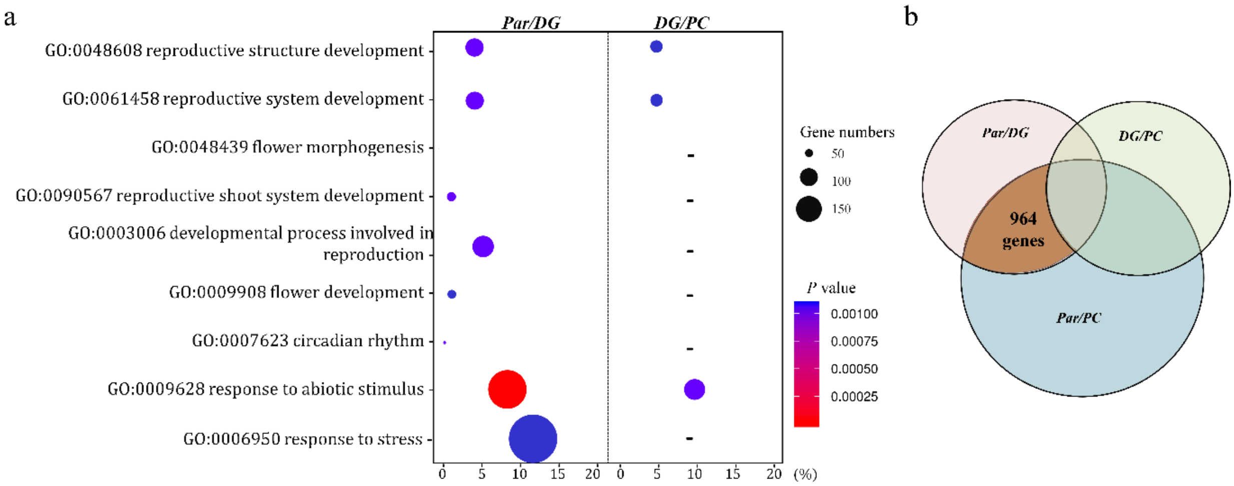

(a) GO terms associated with reproduction and abiotic stress were specifically enriched in the *Par/DG* comparison Diagram showing genes in the selective loci that differentiate between *Par/DG*, but not *DG/PC*. A total of 24 loci and 964 candidate genes were found (Supplementary Data 1, 2).

(b) Genes in the selective loci that differentiate between *Par/DG*, but not between *DG/PC*. A total of 24 loci and 964 candidate genes were found.

Supplementary Figure 7. Fourteen of the 21 *AtJMJ* genes respond to short-term heat stress.

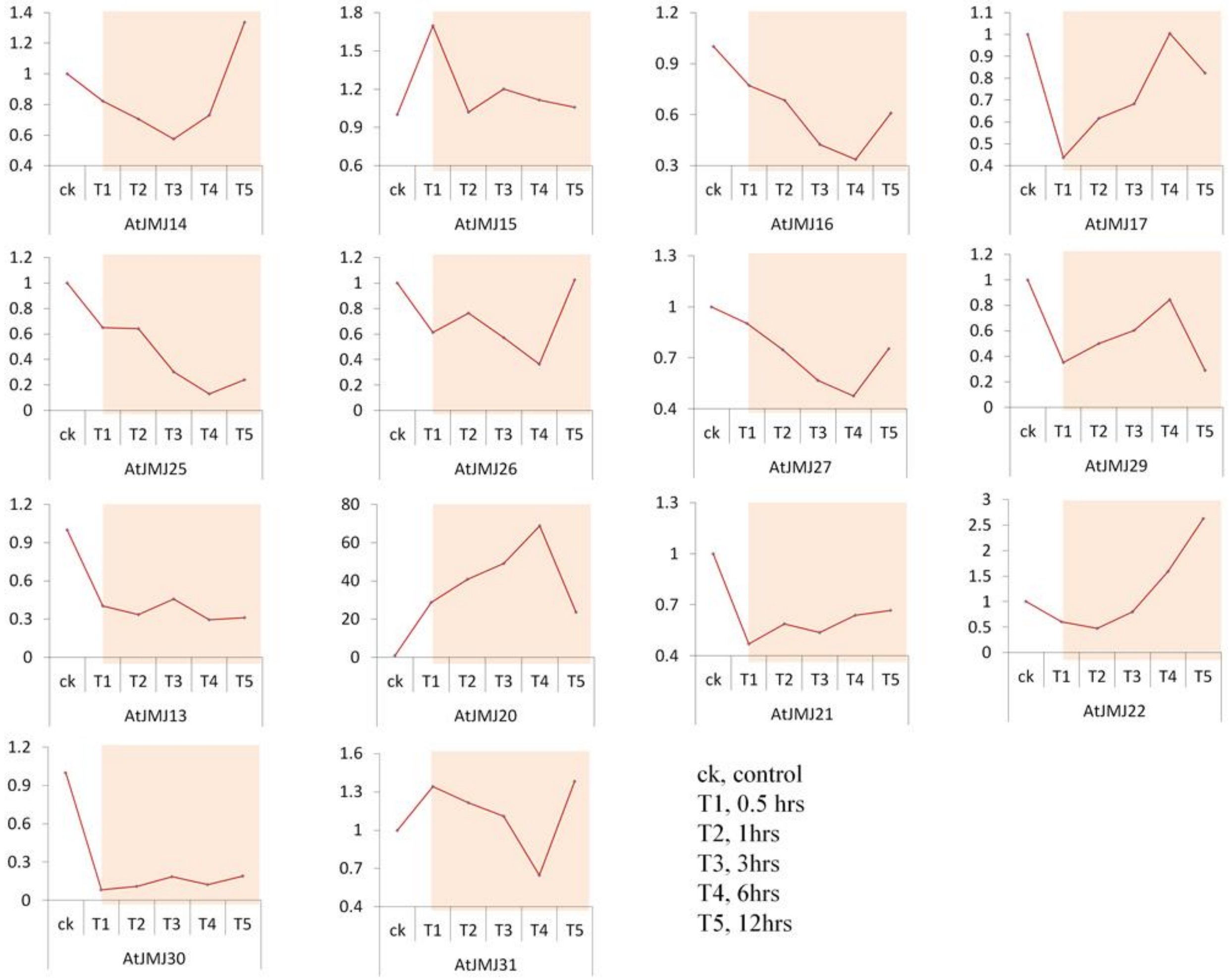

Plots were generated from online Arabidopsis microarray data at TAIR. Treatment: On day 16, heat stress treatment started after 3 h of the light period; samples were taken at 0.5, 1, 3, 6, 12 h after treatment.

Supplementary Figure 8 *BrJMJ18* locates in a pairwise linkage disequilibrium (LD) block around *gQTL^{A09-1}* of *Par/DG.*

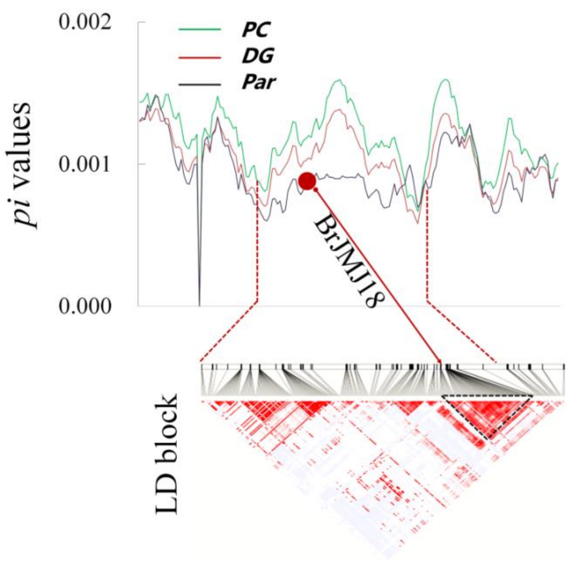

BrJMJ18 was found to be located in a linkage disequilibrium (LD) block of *Par/DG*. The local π value features for the selection sweep on chromosome A09 of *PC*, *DG*, and *Par* around gQTLA09-1 were shown in the Y-axis. Red dot indicates the position of *BrJMJ18*.

Supplementary Figure 9. Expression of *BrJMJ18* under different temperatures.

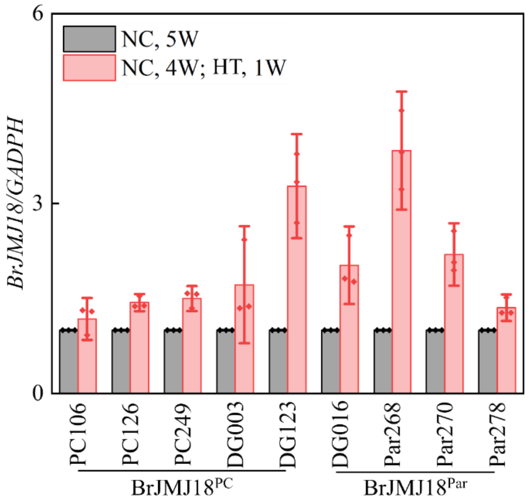

BrJMJ18 was induced by heat, but to a similar degree, in *DG* and *Par*. 3 *BrJMJ18^{PC}*-carrying *PC* (PC016, PC126, PC249) accessions, 2 *BrJMJ18^{PC}*-carrying *DG* (DG003, DG123) accessions, 1 *BrJMJ18^{Par}*-carrying *DG* (DG016) accessions and 3 *BrJMJ18^{Par}*-carrying *Par* (Par268, Par270, Par278) accessions were randomly selected from our germplasm collection. 5-weed-old plants grown under normal conditions (NC), and 4-week-old plants grown under NC following high temperature (HT) for one week were used for *BrJMJ18* Q-PCR. *GADPH* was used as internal control. The values are the mean ± standard deviation from three biological replicates. Source data are provided as a Source Data file.

Supplementary Figure 10. Frequency distribution of *BrJMJ18*^{PC} and *BrJMJ18*^{Par} in the ancient *subsp. rapifera* and *subsp. oleifera* groups, respectively.

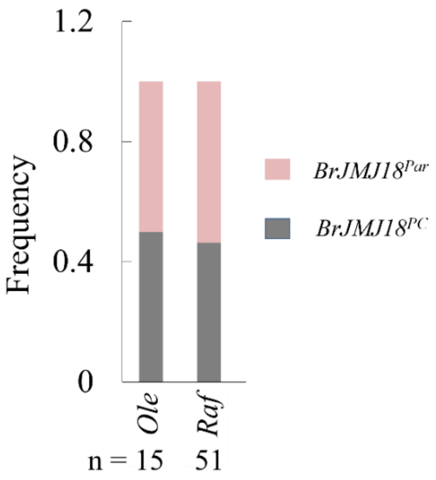

To further identify other natural variants of *BrJMJ18*, we extend the haplotype analysis to all 210 accessions used in this study. No other haplotypes beyond of *BrJMJ18*^{PC} and *BrJMJ18*^{Par} were found. However, it was noteworthy that *BrJMJ18*^{PC} and *BrJMJ18*^{Par} were evenly represented in the ancient *subsp. rapifera* and *subsp. oleifera* groups, respectively.

Supplementary Figure 11. Long-term high temperature exerted a stronger effect on *BrJMJ18*^{PC}- than on *BrJMJ18*^{Par}-carrying lines.

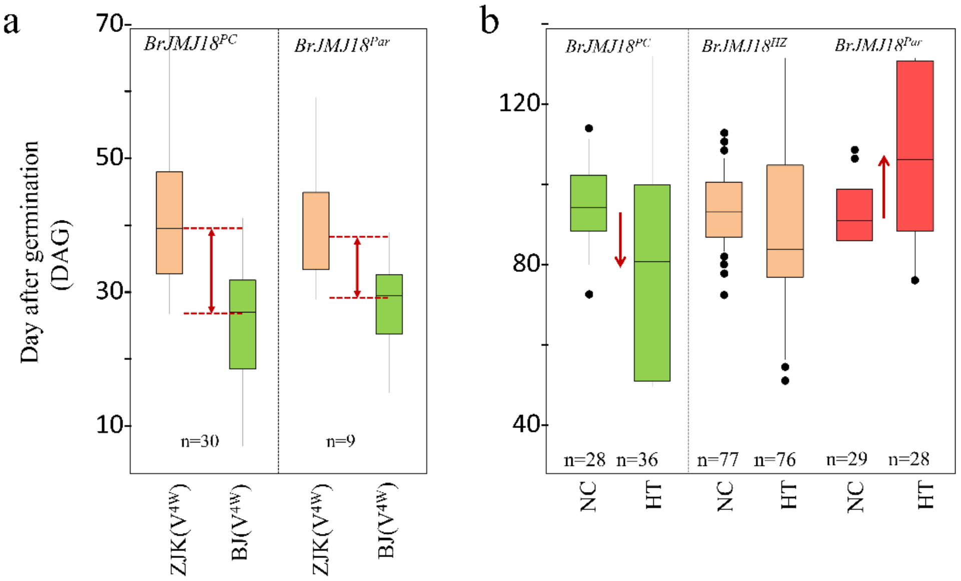

(a) The flowering times of *BrJMJ18*^{PC}- and *BrJMJ18*^{Par}-carrying *DG* lines (n = 39) farmed under natural field conditions in Zhangjiakou (ZJK) and Beijing (BJ), respectively, were used for analysis. The daily temperature of BJ is an average of 5 °C higher than that of ZJK. The earlier flowering induced by high temperature was attenuated significantly in *BrJMJ18*^{Par}-carrying plants. Flowering time, days after germination (DAS), was defined as the number of days from sowing to the appearance of the visible buds.

(b) Flowering time of *BrJMJ18*^{PC}-, *BrJMJ18*^{Par}- and *BrJMJ18*$^{PC/Par}$-carrying lines of the F2 population, which was generated from the F1 crosses of *PC* and *Par*. The flowering time was evaluated under normal conditions (NC) and high temperature (HT) conditions, respectively. NC seedlings were grown at 22°C under a long-day regime (16/8 h day/night) for 4 weeks, and then transplanted in pots under NC until bolting. HT, 4-week seedlings grown under NC were transplanted and moved to HT conditions until bolting. Flowering time, days after sowing (DAS), was defined as the number of days from sowing to the appearance of the visible bud. HT conditions caused early flowering in *BrJMJ18*^{PC}-carrying plants, but delayed flowering in *BrJMJ18*^{Par}-carrying plants (as shown by red arrows).

Data are means ± SD. The box encompasses two middle quartiles, with central line showing median. Whiskers extend to the furthest data point within 1.5 times the interquartile range.

Supplementary Figure 12. Flowering characterizations of the Arabidopsis *BrJMJ18* transgenic plants under different temperatures.

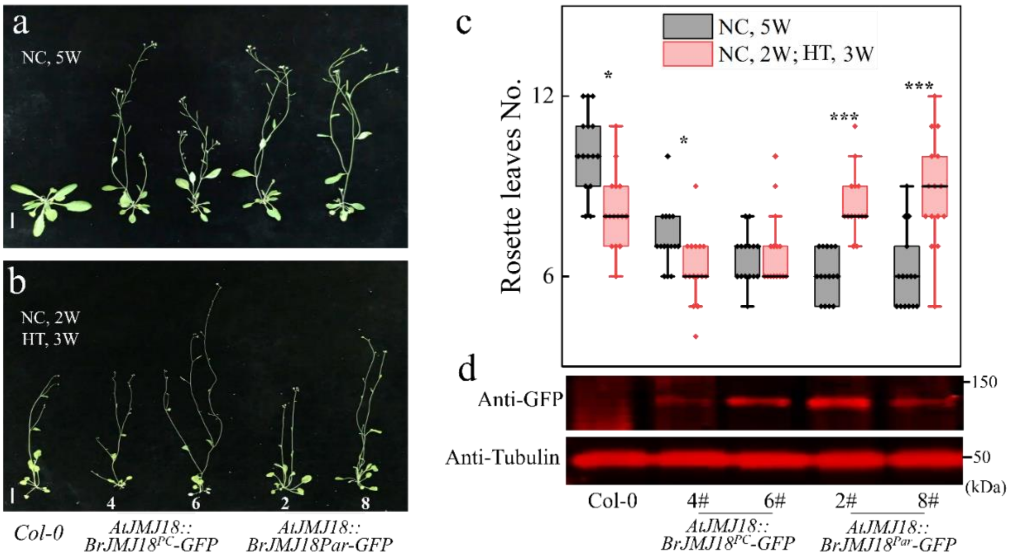

(a) Phenotypes of the *BrJMJ18* transgenic plants grown under normal conditions (NC) for 5 weeks. Both *BrJMJ18*^{PC} and *BrJMJ18*^{Par} greatly promoted flowering under NC. The open reading frames (ORFs) of *BrJMJ18*^{PC} and *BrJMJ18*^{Par} driven by the promotor (2 kb) of *AtJMJ18*, respectively, were transformed into *Arabidopsis Col-0* plants, Transgenic T2 lines with similar protein expressions were used for study. Scale bar, 2 cm.

(b) Phenotypes of the *BrJMJ18* transgenic plants grown under NC conditions for 2 weeks, following another 3-weeks of high temperature (HT) conditions. The increase in temperature accelerated flowering in *Col-0* and *AtJMJ18::BrJMJ18*^{PC}*-GFP* plants, but not in the *AtJMJ18::BrJMJ18*^{Par}*-GFP* plants. Scale bar, 2 cm.

(c) Total primary rosette leaves before bolting of the plants shown in (a) and (b). Data are means ± SD, n = 15. The box encompasses two middle quartiles, with central line showing median. Whiskers extend to the furthest data point within 1.5 times the interquartile range. Asterisks indicate significant differences between NC and HT, two-tailed Student's t test (*$^{*}p < 0.05$, $^{**}p < 0.01$, $^{***}p < 0.001$.*). Col-0, p= 0.0011; 4#, p= 0.026; 2#, p= 9.59×10^{-6}; 8#, p= 9.47×10^{-5}. Source data are provided as a Source Data file. (d) Confirmation of BrJMJ18 protein expression in the transgenic plants under NC conditions by immunoblotting analysis. The anti-GFP antibody recognized a specific endogenous protein of approximately 150 kDa in transgenic, but not in *Col-0* plants. Tubulin, detected by blotting with anti-tubulin antibodies, served as a loading control.

Supplementary Figure 13. Protein sequence alignment of BrJMJ18 and AtJMJs.

Phylogenetic analysis of BrJMJ18 and AtJMJ proteins. BrJMJ18 shows the highest similarity (87%) at the amino acid level to AtJMJ18 among all the 21 AtJMJs of *Arabidopsis*. The phylogenetic tree was generated using the DNAMAN software. Estimated similarities are indicated at each branch.

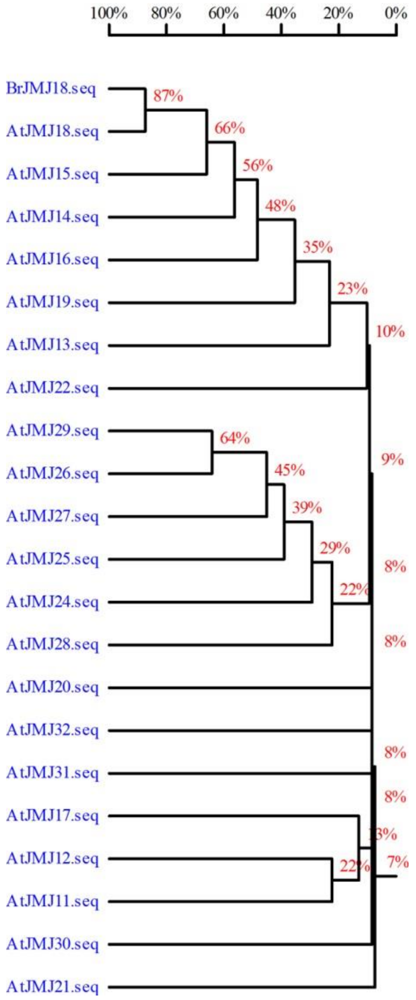

Supplementary Figure 14 BrJMJ18 can't demethylase H3K4me3/2, H3K9me3/2 and H3K27me3/2.

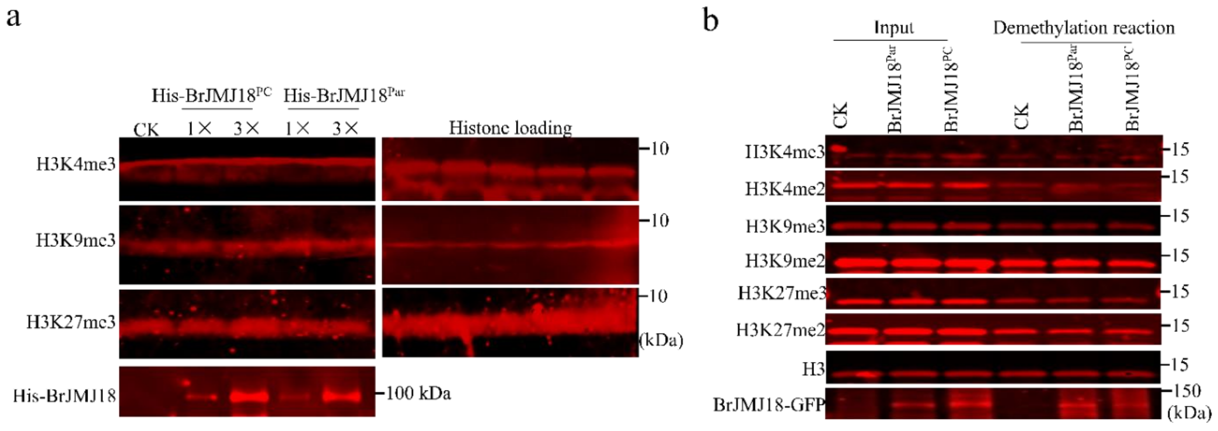

Both BrJMJ18^{PC}-GFP and BrJMJ18^{Par}-GFP demethylate H3K36me3 and H3K36me2, but not H3K4me3 and H3K4me2, H3K9me3 and H3K9me2, and H3K27me3 and H3K27me2, *in vitro*. (a) The two allelic His-BrJMJ18 proteins were affinity-purified from *Escherichia coli* cells. Purified His was used as a negative control. And synthesized Histone H3 peptides with H3K4me3, H3K9me3 and H3K27me3 modification were used as substrates, respectively. (b) The two allelic BrJMJ18-GFP proteins were immunoaffinity-purified from *Arabidopsis BrJMJ18* transgenic lines (*AtJMJ18::BrJMJ18^{orf}-GFP*) and subjected to *in vitro* demethylase analysis using histone from calf thymus as a substrate. *Col-0* was used as a negative control. The *in vitro* demethylation mixture was separated by SDS-PAGE and immunoblotted using the antibodies specified on the left. Immunoaffinity-purified BrJMJ18-GFP proteins were detected with anti-GFP antibodies to confirm equal loading of BrJMJ18^{PC}-GFP and BrJMJ18^{Par}-GFP. H3, detected by blotting with anti-H3 antibodies, served as a loading control. The experiments were repeated three times with similar results.

Supplementary Figure 15. BrJMJ18 antibody specifically recognized BrJMJ18 protein in *Par*.

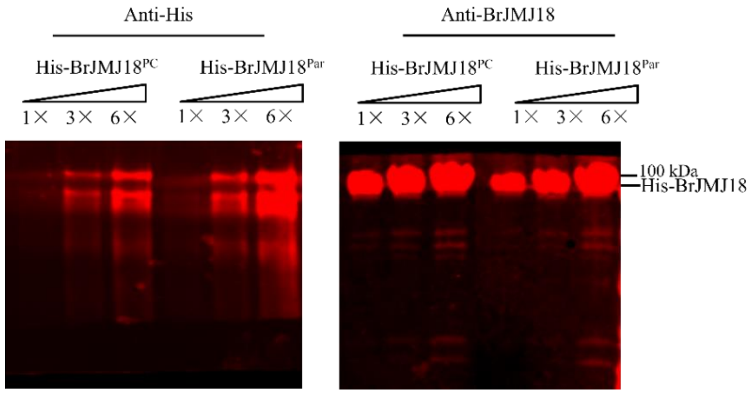

Recombinant His-BrJMJ18$^{PC/Par}$ proteins were affinity-purified from *Escherichia coli* cells. About 0.8-4.8 μg His-BrJMJ18 proteins were separated by SDS-PAGE. Immunoblotting analyses were conducted using anti-His (HT501, TransGen, China) and anti-BrJMJ18 antibodies, respectively. The His antibody identified the His-BrJMJ18 band at about 100 KD and several non-specific bands. BrJMJ18 antibody identified exactly the same His-BrJMJ18 bands with His antibody. The experiments were repeated three times with similar results.

Supplementary Figure 16. The enrichments of BrJMJ18^{PC} and BrJMJ18^{Par} at *BrFLC1*, *2* and *5* under different temperatures.

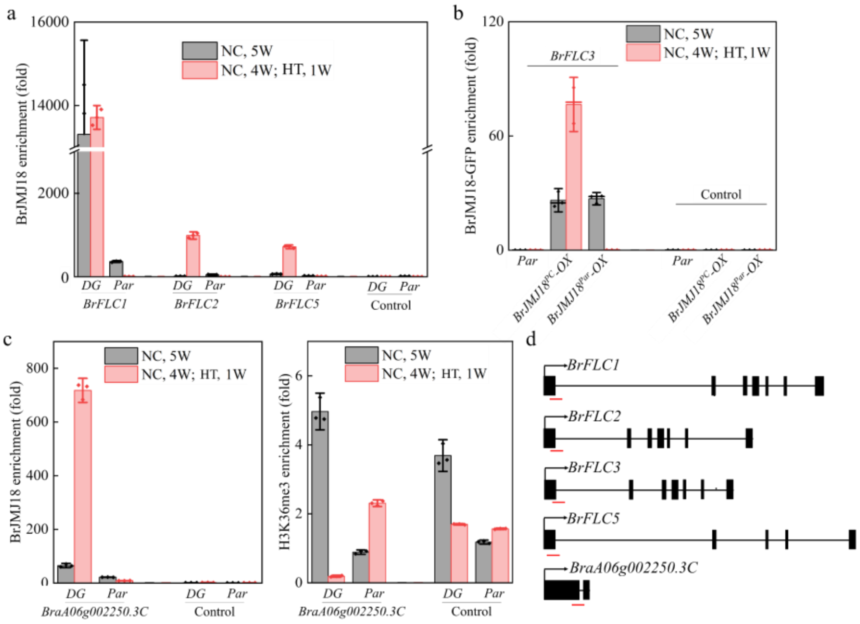

(a) Chromatin immunoprecipitation (ChIP) analysis of the BrJMJ18 and H3K36me3 level across *BrFLCs* were performed in *BrJMJ18^{Par}*-carrying *Par* and *BrJMJ18^{PC}*-carrying *DG* plants. Five-week-old plants grown under normal conditions (NC) and four-week old plants grown under NC following 1-week of high temperature (HT) were used for the analysis. Together with Figure 5A, we showed that in *DG*, BrJMJ18^{PC} binds strongly to *BrFLC1-3,* and high temperature aggravates their binding markedly. While in *Par*, BrJMJ18^{Par} binds strongly to *BrFLC3*, and slightly to *BrFLC1*; and intriguingly, we noticed that high temperature thoroughly disassociated the binding of BrJMJ18^{Par} to *BrFLC3* (Figure 6A). (b) To further test the binding of BrJMJ18 to *BrFLC3*, anti-GFP ChIP-qPCR was conducted using *Par* and *BrJMJ18$^{PC/Par}$-OX* plants grown under NC for 5 weeks or 4 weeks followed by 1 week HT treatment. Under NC, both BrJMJ18^{PC}-GFP and BrJMJ18^{Par}-GFP proteins bind to *BrFLC3*. Binding of BrJMJ18^{PC}-GFP to *BrFLC3* was aggravated by HT, while BrJMJ18^{Par}-GFP protein thoroughly disassociated from *BrFLC3*. (b) To investigate the binding of BrJMJ18 to targets other than *BrFLCs*, we conducted ChIP-qPCR using anti-BrJMJ18 antibody (left penal) and anti-H3K36me3 antibody (right penal) on the randomly selected BrJMJ18-binding gene *BraA06g002250.3C* from Supplementary Data 7. Both allel of BrJMJ18 protein could bind to *BraA06g002250.3C* under NC. High temperature aggravates BrJMJ18^{PC}'s binding to *BraA06g002250.3C* while disassociated the binding of BrJMJ18^{Par}, sharing the same changing pattern of binding to *BrFLC3*. Compared to NC, H3K36me3 levels of *BraA06g002250.3C* loci decreased in *DG* while increased in *Par* under HT. The fold enrichment of BrJMJ18 and H3K36me3 level was calculated using IgG as control. *GADPH* was used as a BrJMJ18-independent control. Control is a locus gene desert regions where BrJMJ18 does not bind. The values are the mean ± standard deviation from three biological replicates. (d) Cartoons showing the analyzed region of *BrFLC*s and *BraA06g002250.3C* by ChIP-qPCR. Black boxes represent the extrons and black bars between them represent introns. Analyzed regions are represented by the red bars. Source data of (a) to (c) are provided as a Source Data file.

Supplementary Figure 17. *BrFLC3* is one of the two expressed *BrFLC*s and the only downregulated *BrFLC* during floral transition in *Par.*

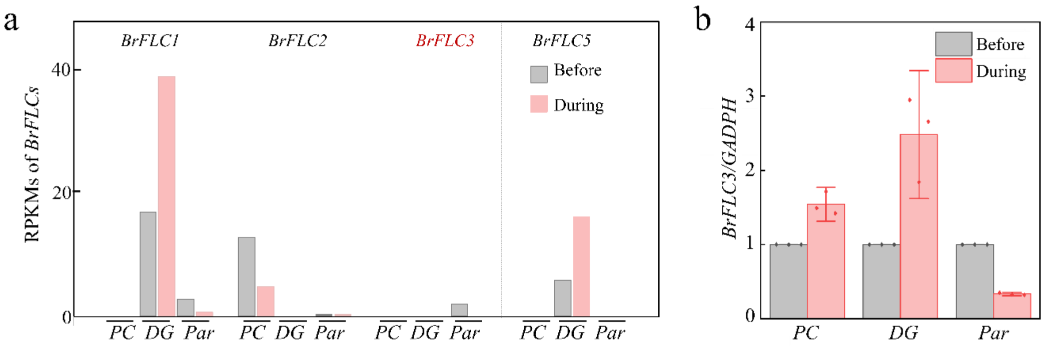

(a) The expression data derived from RNA sequencing data of *PC, DG and Par* plants grown under natural field conditions before and during bolting. (b) *BrFLC3* expression in *PC, DG and Par* grown under natural field conditions before and during bolting detected by Q-PCR. *GADPH* was used as internal control. Source data are provided as a Source Data file.

Supplementary Figure 18. Expression of *AtFLC* and *AtFT* in *AtJMJ18::BrJMJ18^{PC}-GFP* and *AtJMJ18::BrJMJ18^{Par}-GFP* plants under different temperatures.

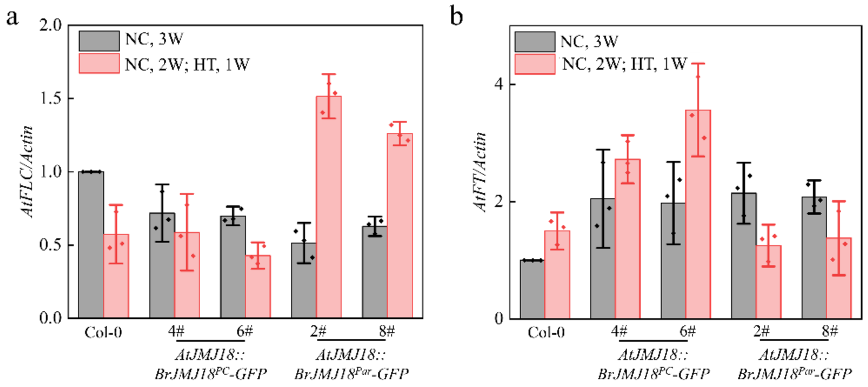

(a) *AtFLC* expression decreased in *AtJMJ18::BrJMJ18^{PC}-GFP* but increased in *AtJMJ18::BrJMJ18^{Par}-GFP* plants upon high temperature treatment. (b) The induction degree of the *AtFT*'s expression by high temperature was in strongly weakened in *AtJMJ18::BrJMJ18^{Par}-GFP* transgenic plants, which is consistent with their flowering time variations.

Three-week old plants grown under normal conditions (NC), and two-week old plants grown under NC following 1-week of high temperature (HT) were used for the analysis. Actin was used as internal control. The values are the mean ± standard deviation from three biological replicates. Source data are provided as a Source Data file.

Supplementary Figure 19. Chromatin immunoprecipitation (ChIP) analysis of H3K36me3 enrichment on the *AtFLC* locus in *BrJMJ1*8 overexpression *Arabidopsis* lines under normal conditions (NC) and high temperature (HT) conditions, respectively.

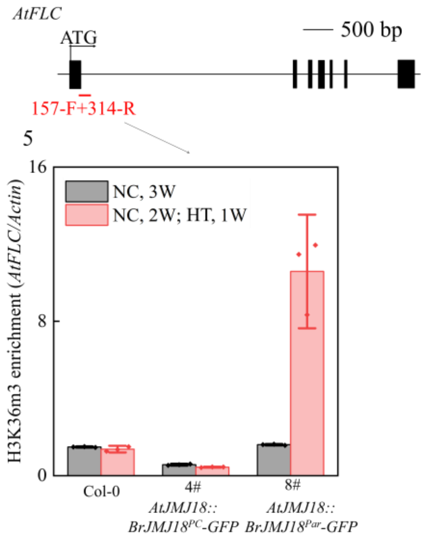

The H3K36me3 level at *AtFLC* was downregulated in *AtJMJ18::BrJMJ18^{PC}-GFP* but upregulated in *AtJMJ18::BrJMJ18^{Par}-GFP* plants by heat. Three-week old plants grown under NC, and two-week old plants grown under NC following 1-week of HT were used for the analysis. *Actin2* was used as internal control of H3K36me3 as reported by Yang, et al, 2017 previously [1]. The values are the mean ± standard deviation from three biological replicates. Source data are provided as a Source Data file.

Supplementary Figure 20. High temperature exerts different impact of the growth of *Par*, *BrJMJ18-OX*, and *BrJMJ18*^{Par}*-CR* plants.

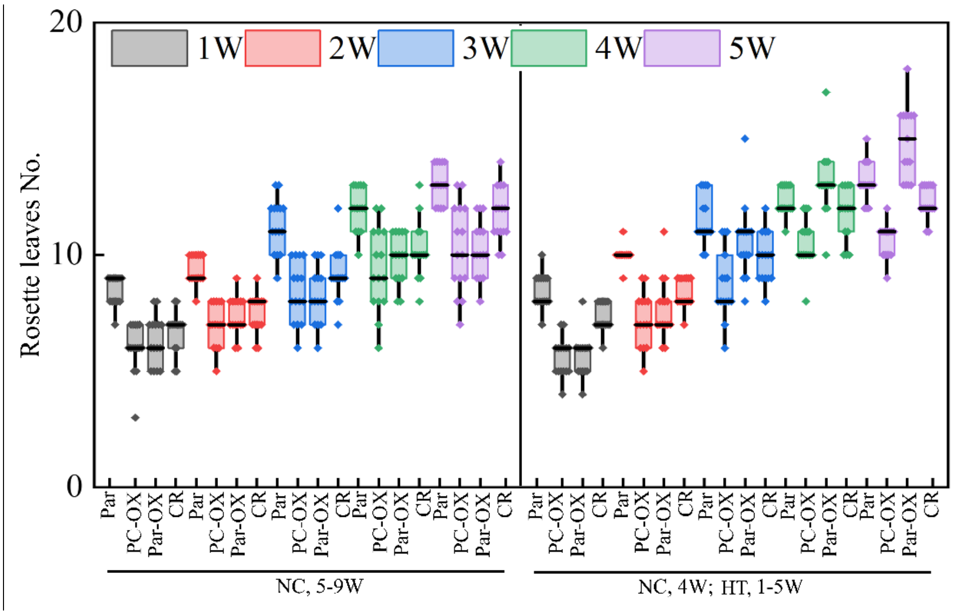

4-week-old *Par*, *BrJMJ18-OX*, and *BrJMJ18*^{Par}*-CR* plants grown under NC condition were moved to NC and HT treatments. True leaves numbers were recorded weekly until flowering. Under NC conditions, the two allelic of *BrJMJ18-OX* plants had fewer true leaves than *Par* plants, while *BrJMJ18*^{Par}*-CR* plants had a leaf count similar to *Par* plants. Under HT treatment, only *BrJMJ18*^{Par}*-OX* plants showed a noteworthy increase in true leaf number, whereas the leaf counts for the other three plant types exhibited no significant difference compared to NC condition. Data are means ± SD. The box encompasses two middle quartiles, with central line showing median. Whiskers extend to the furthest data point within 1.5 times the interquartile range. n = 15. *Par* represents wild type *Par* plants, PC-OX represents *BrJMJ18*^{PC}*-OX* plants, Par-OX represents *BrJMJ18*^{Par}*-OX* plants, CR represents *BrJMJ18*^{Par}*-CR* plants. Source data are provided as a Source Data file.

Supplementary Figure 21. GO analysis of enriched genes identified by BrJMJ18 in *BrJMJ18*^{PC}*-OX* and *BrJMJ18*^{Par}*-OX* plants grown under NC and HT, respectively.

Similar enriched entries between *BrJMJ18*^{PC}*-OX* and *BrJMJ18*$^{Par-}$*OX* plants under NC (a and b) and HT (c and d) were labeled with green and red arrows, respectively.

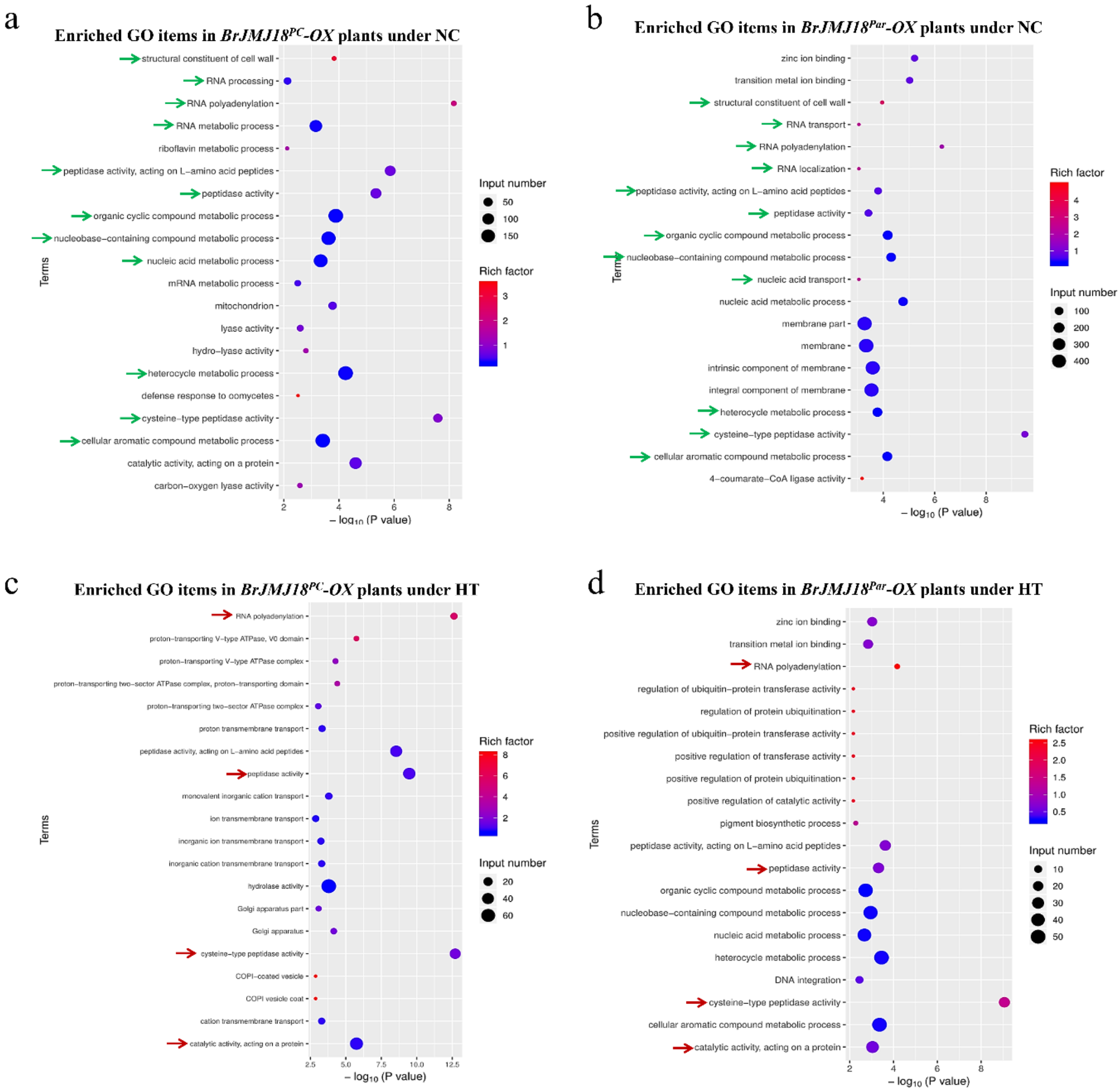

Supplementary Figure 22. GO analysis of DEGs of *BrJMJ18^{PC}-OX* and *BrJMJ18^{Par}-OX* plants under NC and HT, respectively.

In terms of GO categories, there were no big differences between *BrJMJ18^{PC}-OX* and *BrJMJ18^{Par}-OX* plants under NC (a), similar enriched entries were labeled with green arrows. While under HT, GO categories presented significant distinctions between the two plants (b).

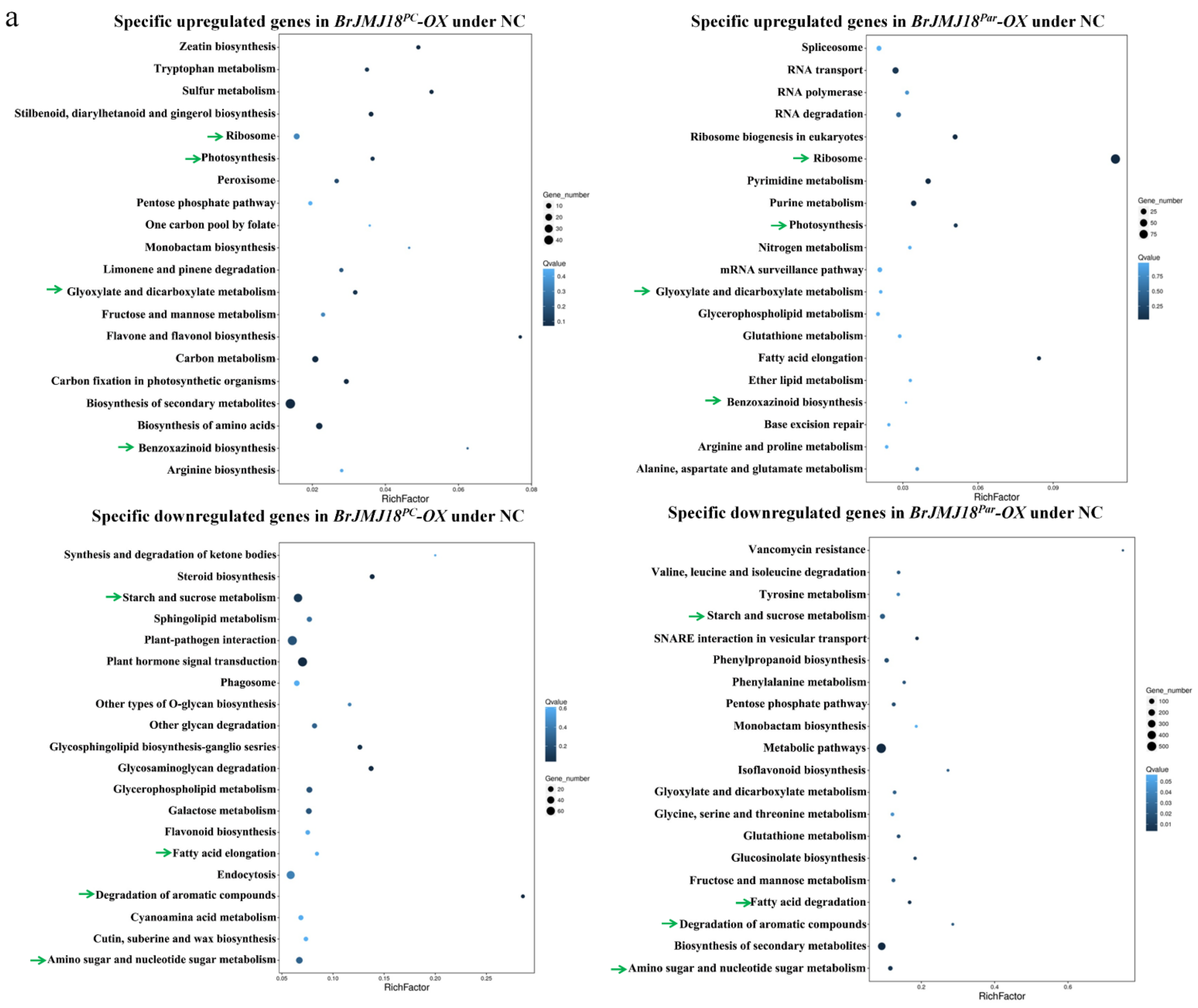

b

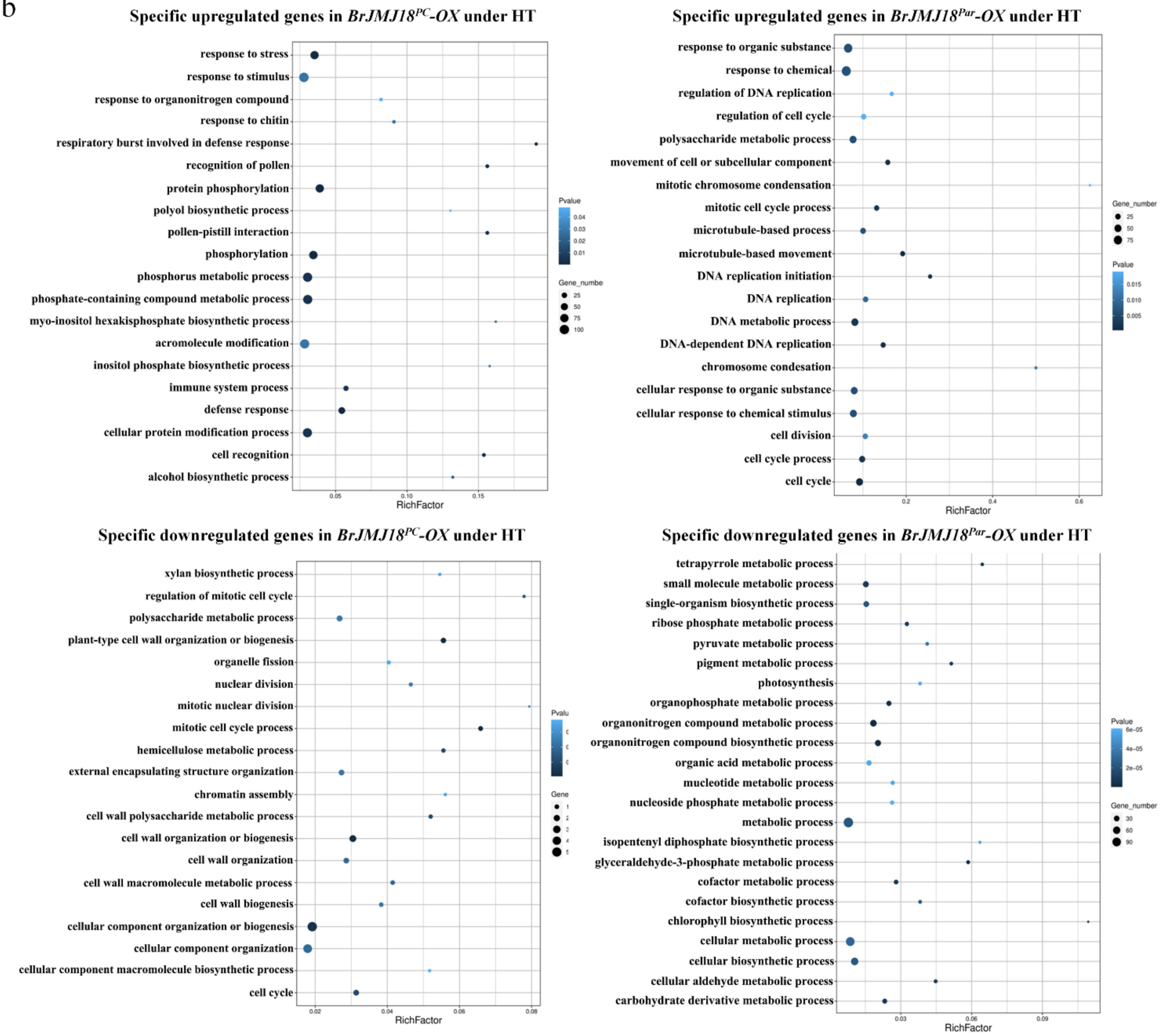

Specific upregulated genes in BrJMJ18PC-OX under HT
response to stress
response to stimulus
response to organonitrogen compound
response to chitin
respiratory burst involved in defense response
recognition of pollen
protein phosphorylation
polyol biosynthetic process
pollen-pistill interaction
phosphorylation
phosphorus metabolic process
phosphate-containing compound metabolic process
myo-inositol hexakisphosphate biosynthetic process
acromolecule modification
inositol phosphate biosynthetic process
immune system process
defense response
cellular protein modification process
cell recognition
alcohol biosynthetic process
0.05
0.10
0.15
RichFactor
Pvalue
0.04
0.03
0.02
0.01
Gene_numbe
25
50
75
100
Specific upregulated genes in BrJMJ18Par-OX under HT
response to organic substance
response to chemical
regulation of DNA replication
regulation of cell cycle
polysaccharide metabolic process
movement of cell or subcellular component
mitotic chromosome condensation
mitotic cell cycle process
microtubule-based process
microtubule-based movement
DNA replication initiation
DNA replication
DNA metabolic process
DNA-dependent DNA replication
chromosome condesation
cellular response to organic substance
cellular response to chemical stimulus
cell division
cell cycle process
cell cycle
0.2
0.4
0.6
RichFactor
Gene_number
25
50
75
Pvalue
0.015
0.010
0.005
Specific downregulated genes in BrJMJ18PC-OX under HT
xylan biosynthetic process
regulation of mitotic cell cycle
polysaccharide metabolic process
plant-type cell wall organization or biogenesis
organelle fission
nuclear division
mitotic nuclear division
mitotic cell cycle process
hemicellulose metabolic process
external encapsulating structure organization
chromatin assembly
cell wall polysaccharide metabolic process
cell wall organization or biogenesis
cell wall organization
cell wall macromolecule metabolic process
cell wall biogenesis
cellular component organization or biogenesis
cellular component organization
cellular component macromolecule biosynthetic process
cell cycle
0.02
0.04
0.06
0.08
RichFactor
Pvalu
Gene
Specific downregulated genes in BrJMJ18Par-OX under HT
tetrapyrrole metabolic process
small molecule metabolic process
single-organism biosynthetic process
ribose phosphate metabolic process
pyruvate metabolic process
pigment metabolic process
photosynthesis
organophosphate metabolic process
organonitrogen compound metabolic process
organonitrogen compound biosynthetic process
organic acid metabolic process
mucleotide metabolic process
nucleoside phosphate metabolic process
metabolic process
isopentenyl diphosphate biosynthetic process
glyceraldehyde-3-phosphate metabolic process
cofactor metabolic process
cofactor biosynthetic process
chlorophyll biosynthetic process
cellular metabolic process
cellular biosynthetic process
cellular aldehyde metabolic process
carbohydrate derivative metabolic process
0.03
0.06
0.09
RichFactor
Pvalue
6e-05
4e-05
2e-05
Gene_number
30
60
90

Supplementary Figure 23. Flowering characterizations of transgenic *Arabidopsis* plants of *BrJMJ18^PC^*, *BrJMJ18^Par^*, and its variants under different temperatures.

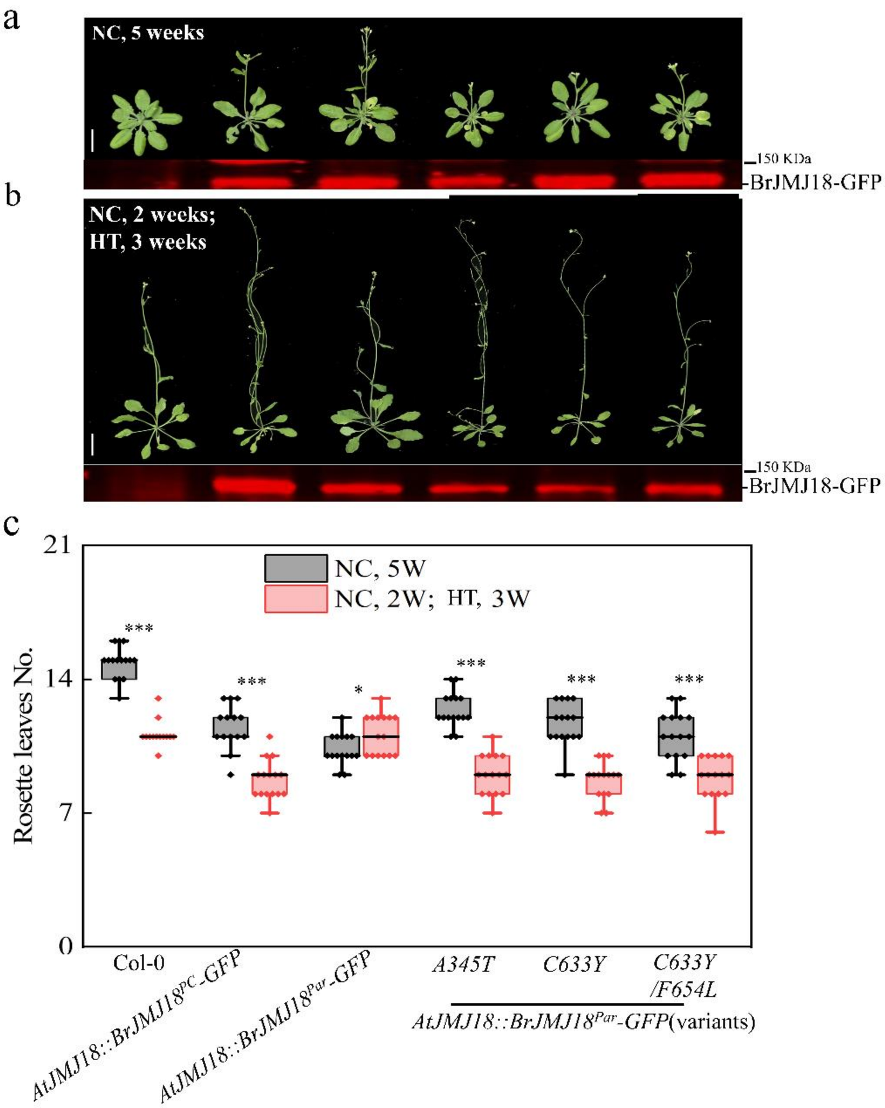

(a) Under NC, transgenic plants of *BrJMJ18*$^{Par(A345T)}$, *BrJMJ18*$^{Par(C633Y)}$ *and BrJMJ18*$^{Par(C633Y)/(F654L)}$ flowered at approximately the same time as *BrJMJ18*^{Par}- and *BrJMJ18*^{PC}-expressing plants; (b) under HT, high temperature significantly delayed flowering in the *BrJMJ18*^{Par} plants, but not in the *BrJMJ18*$^{Par(A345T)}$, *BrJMJ18*$^{Par(C633Y)}$, and *BrJMJ18*$^{Par(C633Y)/(F654L)}$ plants. Besides, transgenic plants of *BrJMJ18*$^{Par(A345T)}$, *BrJMJ18*$^{Par(C633Y)}$, and *BrJMJ18*$^{Par(C633Y)/(F654L)}$ exhibited very similar morphological characteristics to *BrJMJ18*^{PC}-expressing plants. BrJMJ18-GFP proteins were detected with anti-GFP antibody to confirm equal expression of exogenous genes. Scale bar = 2 cm. (c) Phenotypes of the *BrJMJ18s* transgenic *Arabidopsis* plants grown under NC conditions (5 weeks), and HT conditions (plants were grown under NC conditions for 2 weeks following another 3-weeks under high temperature), respectively, were used for flowering time evaluation. In (c), data are means ± SD, n = 15. The box encompasses two middle quartiles, with central line showing median. Whiskers extend to the furthest data point within 1.5 times the interquartile range. Asterisks indicate significant differences between NC and HT, Student's t-test (*p < 0.05, **p < 0.01, ***p < 0.001). Col-0, p= 1.13E×10^{-8}; PC-4#, p= 3.33×10^{-5}; Par-8#, p= 0.034; A345T, p= 4.66×10^{-7}; C633Y, p= 3.32×10^{-6}; C633Y/F654L, p= 4.75×10^{-5}. Source data are provided as a Source Data file.

Supplementary Figure 24. A combined summary of estimated demographic modeling and written history of leafy *B. rapa* domestication events.

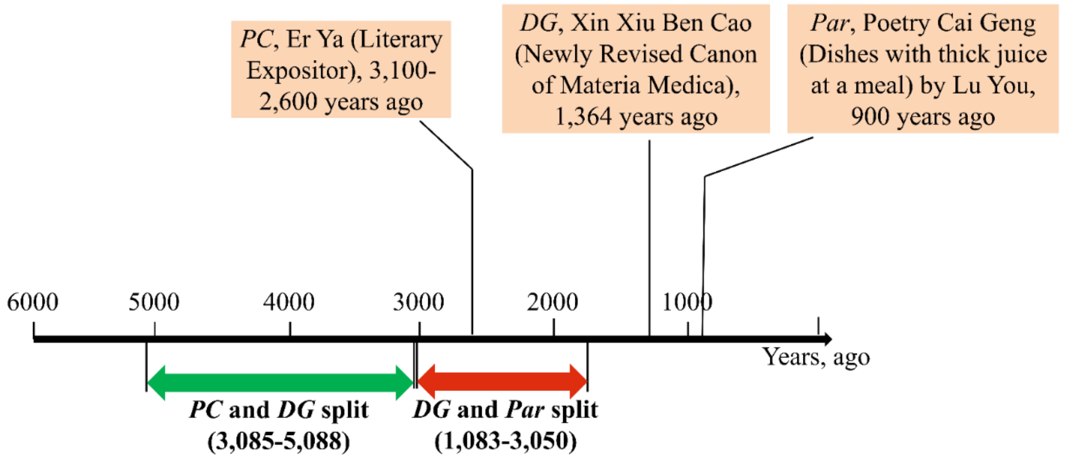

The black horizontal arrow represents time scale. The pink callouts above represent the historical written records of *PC*, *DG* and *Par* (Subspecies, source, time). The green and red bars below represent the estimated split time of *DG* and *Par* based on our ∂a∂i analysis. Turnips was described as "Feng" (葑) in the oldest Chinese poetry collection, Shi Jing (Classic of Poetry), about 3,100-2,600 years ago. And *PC* was called "Song" (菘) and was firstly described in the oldest Chinese encyclopedia, Er Ya (Literary Expositor), about 3,000-2,700 years ago in our country. Additionally, the word "Song" was used as a general term for leafy *B. rapa* crops, and different types of "Song" were recorded. For instance, three types of "Song" were recorded in Xin Xiu Ben Cao (Newly Revised Canon of Material Medica, 1364 years ago): "Niu Du Song (Big-Tummy Song)" with large and curved leaves, "Zi Song (Purple Song)" with purple and slightly bitter leaves (probably purple Pak Choi), and "Bai Song (White Song)" with white petioles and dark green leaves. According to the morphological features, the Bai Song is likely to be the *DG*. With regarding to *Par*, *Par* was recorded to be cultivated in the Taihu Lake area of China in the Song Dynasty (AD 960-AD 1,279) and was mentioned in a poetry Cai Geng by poet Lu You (AD 1,125 – AD 1,210). The recorded order of *PC*, *DG*, and *Par* corresponds to the appearance times inferred from genome sequencing. However, historical records of these times are indeed behind the predicted times from genome sequencing. We speculate that this could be due to the fact that species are generally not recorded immediately after their formation but before they are fully developed and widespread.

Supplementary Figure 25. A working model of BrJMJ18^{Par} under different temperature conditions.

Under NC, the overexpression of BrJMJ18^{PC} and BrJMJ18^{Par} downregulates *BrFLC3* by demethylating its H3K36me3/2, consequently promoting flowering. Under high temperature conditions, the flowering promotion function of BrJMJ18^{PC} is strengthened in *BrJMJ18^{PC}-OX* plants. However, in *BrJMJ18^{Par}-OX* plants *BrJMJ18^{Par}* represses early flowering via a mechanism in which the binding and subsequent demethylation activity of BrJMJ18^{Par} of *BrFLC3*, is notably weakened by heat. At the same time BrJMJ18^{Par} promotes vegetative growth by regulating chlorophyll biosynthesis. The symbol "↓" represents the positive regulation to the downstream factors and "⊥" represents negative regulation to the downstream factors.

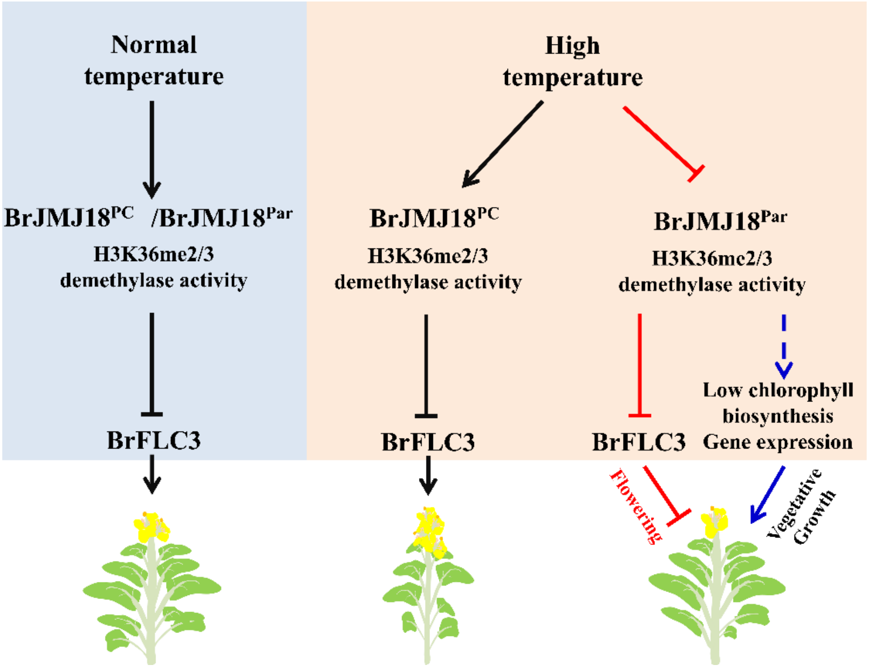

Supplementary Figure 26. *BrFLC3* showed the lowest genomic similarity to *AtFLC* among all four *BrFLC*s.

Genomic sequences of *AtFLC* and *BrFLC1-3*, *5* were used for the analysis. The phylogenetic tree was constructed using MEGA 7.0 software.

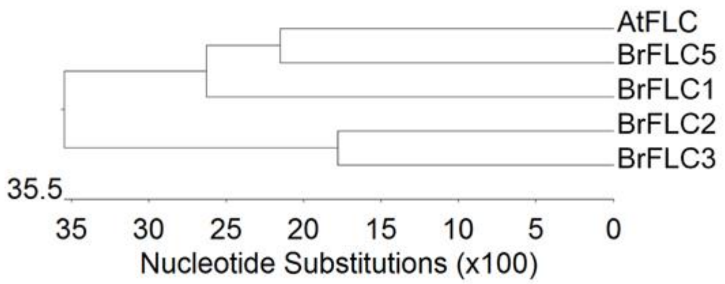

Supplementary Figure 27. Haplotype analysis of *BrFLC3* in *PC*, *DG*, and *Par* showed that 126 of the 135 lines were classified into seven haplotype groups; however, none of them was specific to *Par*.

Par represents *B. rapa* subsp. *chinensis* var. *parachinensis*, *DG* represents *B. rapa* subsp. *chinensis* var. *Dark-Green*, *PC* represents *B. rapa* subsp. *chinensis* (pak choi).

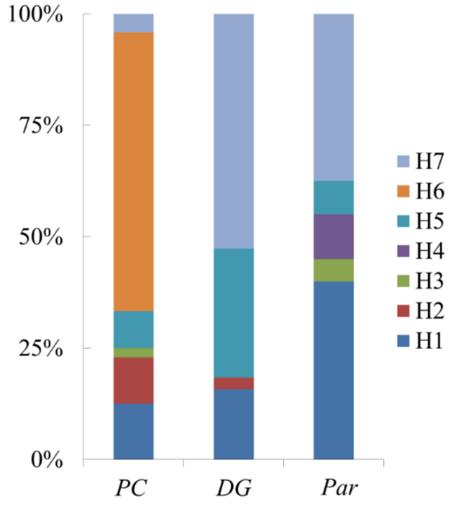

Supplementary Table 1. Geographical and pedigree information of the 210 *Brassica rapa* lines.

	Sequencing ID	Collection number	Acession number	Chinese name	Name	Origin
subsp.oletifera(Ole)	SRR3201745	(Cheng et al., 2016) [2]				
	SRR3201999					
	SRR3201729					
	SRR3201998					
	SRR3201785					
	SRR3201751					
	YY415	W-SO-035	W-SO-035	/	/	Bangladeshi
	YY416	W-SO-039	W-SO-039	/	/	Bangladeshi
	YY420	W-YS-033	W-YS-033		/	Germany
	YY421	W-YS-143	W-YS-143	/	/	India
	YY149	101-180		/	/	/
	YY316	YY672-1		/	/	Tibet, China
	YY306	6300-1		/	/	Tibet, China
	YY315	YY671-1		/	/	Tibet, China
	YY305	10400-1		/	/	Tibet, China
sbusp.rapifera (Raf)	SRR3204346	(Cheng et al., 2016) [2]				
	SRR3203997					
	SRR3203915					
	SRR3203830					
	SRR3204355					
	SRR3203657					
	SRR3203802					
	SRR3203777					
	SRR3203854					
	SRR3203920					
	SRR3203805					
	SRR3203839					
	SRR3203886					
	SRR3204261					
	SRR3204245					
	SRR3204221					
	SRR3203925					
	SRR3203633					
	SRR3204219					
	SRR3204020					
	SRR3203804					
	SRR3203781					
	SRR3203680					
	SRR3203685					
	SRR3203779					
	SRR3203669					
	SRR3203914					
	SRR3203985					

	SRR3203684					
	SRR3203644					
	SRR3203803					
	SRR3203918					
	SRR3203853					
	SRR3204220					
	SRR3204024					
	WJ289	721.0002		/	/	
	WJ287	408.0075		/	Melon Red Top	Pakistan
	WJ297	408.0032		/	purple top white globe	Europe
	WJ425	408.0018		/	PURPLE TOP WHITE GLOBE	America
	WJ295	408.0089		/	/	Europe
	WJ389	408.0054		/	/	Nepal
	WJ384	408.0049		/	Parple TOP white globe	FAO
	WJ382	408.0047		/	purple top WG	Nepal
	WJ286	408.0066		/	Golden Ball	Pakistan
	WJ377	408.0042		/	QUARANTINO	FAO
	WJ378	408.0043		/	NATALINO	FAO
	WJ361	408.0026		/	MLLAN PLIRPEE TOP FORCLNG	UK
	WJ292	ECD-02		/	ECD-02	Europe
	WJ367	408.0032		/	MARBLE GREEN TOP	UK
	WJ290	MM		/	MM	Europe
	WJ364	408.0029		/	IMPERIAL GREEN GLOBE	UK
subsp.var.narinosa (Nar)	TC116	18Q-116		如皋乌塌菜 2	RGWTC2	/
	TC126	18Q-126		/	/	/
	TC132	18Q-132		百叶塌菜	BYTC	/
	TC105	18Q-105		/	/	/
	TC003	TC3		/	/	/
	TC002	TC2		/	/	/
	TC094	94#		/	/	/
	TC001	TC1		/	/	/
	TC095	95#		/	/	/
sbusp.chinensis (PC)	PC002	18Q-104		蚕白菜	CBC	Southern China
	PC137	18Q-137		长梗白菜	CGBC	Southern China
	PC114	11N-303		紫油菜 X 百惠	ZYCXBH	/
	PC094	18Q-94		紫油菜(小)	ZYC(X)	/
	PC261	12N-310		紫油菜	ZYC	Northern China
	PC001	18Q-97		红油菜.青梗菜(高)	HYC.QGC(G)	/
	PC121	10N-55		矮抗.黑叶 4.矮抗	AK.HY4.AK	Southern China
	PC128	09N-3		黑叶 4 月.青梗菜	HY4Y.QGC	/
	PC131	11N-336		青梗菜	QGC	/
	PC010	18Q-10		华京.上四 2.华京(矮)	HJ.SS2.HJ(A)	/

	PC011	18Q-11		华冠×上海四月 8(矮)	HG×SHSY8(A)	/
	PC012	18Q-12		18N-183	18N-183	/
	PC099	11N-1		上海 4 月 4	SH4Y4	Southern China
	PC100	10N-53		东方矮抗. 上海 4 月	DFAK. SH4Y	/
	PC009	18Q-9		黑叶四月混	HYSYH	/
	PC098	10N-350		上海 4 月	SH4Y	Southern China
	PC249	12N-11		中蔬五月慢深	ZSWYMS	Northern China
	PC096	04N-351		上海 5 月	SH5Y	Southern China
	PC021	18Q-127		味美菜(小松菜)	WMC(XSC)	/
	PC023	18Q-131		抗热 605 青菜	KR605QC	/
	PC005	18Q-115		虹明青菜	HMQC	/
	PC020	18Q-125		青轴パクチョィ	QINGYOU	/
	PC111	18Q-111		勺菜	SC	/
	PC003	18Q-106		矮箕白菜	AJBC	Southern China
	PC104	04N-353		矮抗青(灰)	AKQ(H)	Southern China
	PC032	18Q-32		四季青深	SJQS	/
	PC008	18Q-122		台湾青江白菜	TWQJBC	/
	PC129	18Q-129		日本大头青江白菜	RBDTQJBC	/
	PC132	08N-369		澳洲抗热清江白	AZKRQJB	Austrilia
	PC250	04N-258		黄金白菜	HJBC	Southern China
	PC120	11N-340		澳抗显	AKX	Austrilia
	PC106	10N-697		四季青	SJQ	Southern China
	PC113	11N-2		春华 x 蓟县新四(直)	CHxJXXS(Z)	/
	PC125	10N-696		春华	CH	Southern China
	PC243	12N-514		城研青菜	CYQC	Northern China
	PC244	12N-519		改良 21	GL21	Southern China
	PC013	18Q-13		北极直	BJZ	/
	PC097	10N-56		上海 5 月 3.华玉(旺)	SH5Y3.HY(W)	/
	PC115	11N-467		金夏莳 P1	JXZP1	Japan
	PC126	11N-339		华玉	HY	Japan
	PC034	18Q-34		夏青(浅)	XQ(Q)	/
	PC242	12N-510		冬莳	DZ	Japan
	PC127	07N-337		夏帝	XD	Japan
	PC116	11N-517		日本华光 P1	RBHGP1	Japan
	PC122	10N-687		白沙一号	BSYH	Southern China
	PC117	11N-513		旗舰 P1	QJP1	Northern China
	PC119	05N-217		早生华京	ZSHJ	Japan
	PC133	09N-548		华冠抗	HGK	Japan
	PC118	10N-688		华冠大叶	HGDY	Japan
	PC130	10N-689		华冠小	HGX	Japan
ssp. chinensis var. parachinensis (Par)	Par165	18Q-190		宝青 50 天油心	BQ50TYX	/
	Par079	18Q-178		绿满园香脆，高	LMYXC，G	/
	Par091	18Q-181		加拿大菜心	JNDCX	/
	Par125	18Q-187		翠绿 80 天菜心	CL80TCX	/
	Par124	08N-510		60 天菜心	60TCX	Southern China
	Par013	18Q-160		金苗美绿 702 晚	JMML702 W	/
	Par117	18Q-186		油绿 702 菜心	YL702CX	/

	Par270	11A9-50③		长合 70 天	CH70T	Southern China
	Par110	18Q-185		金韩 70 天菜心	JH70TCX	/
	Par272	10A9-252①		迟 80 天(柳)	C80T(L)	Southern China
	Par001	18Q-155		金满田 80 天(早直)	JMT80T(ZZ)	/
	Par269	11A9-224⑤		利隆矮脚 70 天早	LLAJ70TZ	Southern China
	Par010	18Q-159		东莞 70 天尖叶(从)	DZ70TJY(C)	/
	Par055			粤友迟心 1 号(晚)矮柳	YYCX1H(W)A L	/
	Par099	18Q-183		圣农粗条 90 天	SNCT90T	/
	Par023	18Q-162		60 天(周)(晚)	60T(Z)(W)	/
	Par006	18Q-158		迟 80 天(大叶，晚) 翠绿	C80T(DY，W) CL	/
	Par049	18Q-167		坡头 31 号 早	PT31H Z	/
	Par033	18Q-164		一哥白沙 45 天 直中	YGBS45T ZZ	/
	Par063	18Q-174		广东 1 只花(晚 大叶)(早)	GD1ZH(W DY)(Z)	/
	Par056	18Q-170		金韩秋菊 直立	JHQJ ZL	/
	Par057	18Q-171		新世纪 20 号，中	XSJ20H，Z	/
	Par089	18Q-180		45 天菜心，早叶少	45TCX，ZYS	/
	Par038	18Q-166		019 (皱)	019 (Z)	/
	Par271	11A-74②		中南 80 天油绿甜菜心	ZN80TYLTCX	Southern China
	Par061	18Q-173		日本油美 1 号(晚)	RBYM1H(W)	/
	Par077	18Q-177		新苗 T28 细	XMT28 X	/
	Par037	18Q-165		4 号早	4HZ	/
	Par051	18Q-168		香港油青(早)	XGYQ(Z)	/
	Par265	10A9-31①		范记 50 天早	FJ50TZ	Southern China
	Par002	18Q-156		红亮 85 天(晚)	HL85T(W)	/
	Par274	11A9-8⑦		利农 5 号(粗)	LN5H(C)	Southern China
	Par275	11A9-15①		宝丰矮脚大叶	BFAJDY	Southern China
	Par031	18Q-163		利隆矮脚 45 天(早)	LLAJ45T(Z)	/
	Par021	18Q-161		金韩 60 天，早	JH60T，Z	/
	Par268	10A-96①		圣农绿宝柳叶 70 天菜心	SNLBLY70TCX	Southern China
	Par277	10A9-235③		惟勤全年粗条(柳)	WQQNCT(L)	Southern China
	Par273	11A9-142②		欣农澳洲 5 号早	XNAZ5HZ	Southern China
	Par278	11A9-198①		广良油青(芽)	GLYQ(Y)	Southern China
	Par058	18Q-172		农鑫抗病油青(旺)(开展，晚)	NXKBYQ(W)(KZ，W)	/
	Par266	11A9-148③		广研菜场 60 天油青(晚)	GYCC60TYQ(W)	Southern China
	Par264	10A9-73①		弘农东莞 30 天	HNDZ30T	Southern China
	Par276	11A9-2③		美青一号(晚)	MQYH(W)	Southern China
	Par267	11A9-156⑧		坡头 60 天浅	PT60TQ	Southern China
subsp.chinensis (DG)	DG001	HY/NB1		苗丰黑叶白菜(直)	MFHYBC(Z)	/
	DG016	18Q-36		长筒白	CTB	/
	DG041	18Q-123		葵扇黑叶白菜	KSHYBC	/
	DG247	247#		白玫瑰深	BMGS	Japan
	DG006	HY/NB6		广良黑叶白 P 深	GLHYBPS	/
	DG024	HY/NB24		华盛玉玲珑 P 浅	HSYLZPQ	/
	DG109	109#		I(典)	I(D)	Southern China

	DG023	HY/NB23		绿园四季黑叶	LYSJHY	/
	DG004	HY/NB4		夏宝黑叶小叶	XBHYXY	/
	DG019	HY/NB19		玉兔杂交黑叶	YTZJHY	/
	DG021	HY/NB21		玉兔杂交黑叶 P 直	YTZJHYPZ	/
	DG112	112#		湛江白菜	ZJBC	Southern China
	DG135	18Q-135		光泽矮脚黑叶白菜	GZAJHYBC	/
	DG008	HY/NB8		佳信港种奶白菜(杂)	JXGZNBC(Z)	/
	DG003	HY/NB3		尖峰矮脚黑叶白	JFAJHYB	/
	DG040	18Q-40		尖峰黑叶白	JFHYB	/
	DG018	HY/NB18		旺田黑叶	WTHY	/
	DG038	18Q-38		金冠黑叶	JGHY	/
	DG005	HY/NB5		米高梅黑叶深	MGMHYS	/
	DG039	18Q-39		米高梅黑叶浅	MGMHYQ	/
	DG002	HY/NB2		欣农中脚葵扇黑叶甜白菜(深)	XNZJKSHYTBC(S)	/
	DG022	18Q-37		严选黑叶白菜	YXHYBC	/
	DG054	18Q-54		白公主矮脚奶白	BGZAJNB	/
	DG129	129#		中脚黑叶浅	ZJHYQ	/
	DG017	HY/NB17		XP001P 深	XP001PS	/
	DG049	18Q-49		玉龙 262	YL262	/
	DG025	HY/NB25		玉龙 262 展	YL262Z	/
	DG108	108#		惠州矮脚奶白(帮厚)	HZAJNB(BH)	Southern China
	DG136	18Q-136		惟勤香港矮脚奶白菜	WQXGAJNBC	/
	DG026	HY/NB26		金地奶白菜旺	JDNBCW	/
	DG007	HY/NB7		10 龙湖 161-3 深展	10LH161-3SZ	/
	DG009	HY/NB9		斗白(大)	DB(D)	/
	DG053	18Q-53		斗白大	DBD	/
	DG246	246#		鹤斗白中	HDBZ	Southern China
	DG110	110#		奶白 A	NBA	Southern China
	DG123	123#		奶白(弱)	NB(R)	Southern China
	DG014	18Q-58		上海五月 3×中脚奶白 2(塌地)	SHWY3×ZJNB2(TD)	/
	DG111	111#		中脚黑叶深	ZJHYS	Southern China
	DG013	HY/NB13		黑叶四月混.(乌白叶.Ⅱ 2)2(浅)	HYSYH.(WBY.Ⅱ 2)2(Q)	/
	DG012	18Q-55		上海五月 3×(乌白叶.Ⅱ 2)2(脉显)	SHWY3×(WBY.Ⅱ 2)2(MX)	/
	DG048	18Q-48		/	/	

Supplementary Table 2. The parameters and confidence intervals inferred in ∂a∂i simulation.

These parameters correspond to those displayed in Figure 1 A. The 95% confidence interval (CI) was calculated using the Godambe bootstrapping method. Unit of absolute effective population size: Individual; unit of absolute time: Yearable.

Parameter	Relative value (v)	95% CI	Absolute value (μ1 = 1.5E-8)	95% CI	Absolute value (μ2 = 9E-9)	95% CI
Ne_{DG}	0.028	0.0038	627	85	1045	143
Ne_{Par}	0.35	0.13	7938	2858	13250	4730
T1	0.027	0.006	1220	278	2038	451
T2	0.04	0.005	1838	230	3050	382
θ	10446.25					
Log-likelihood	20487.5					
Ne_{PC}			5760		7250	

Supplementary Table 3. Summary of the QTL mapping of flowering loci in which *BrFLC*s are candidates in *Brassica rapa* crops.

Reference	Population used	Planting conditions used for collecting flowering time data			Trait	QTL ID in the paper	Contribution Explain (%)	BrFLCs contained
		Vernalization	Growth temperature	Duration of day				
Lou et al, 2008 [3]; Zhao et al, 2010 [4]	DH population 38 derived from PC-175 (pak choi) cross with YS-143 (Yellow Sarson)	without vernalization	greenhouse 24/18℃ (day/night)	16 h	Flowering Time without vernalization	FLQTL-1 (A02)	63.8	BrFLC2
		vernalized in the dark at 5℃ for 18d			Flowering time after 18d vernalization	FLQTL-2 (A02)	30.9	BrFLC2
		vernalized in the dark at 5℃ for 31d			vernalization response after 18d vernalization	VRQTL-1 (A02)	71.9	BrFLC2
					vernalization response after 31d vernalization	VRQTL-2 (A02)	77.1	BrFLC2
Li et al, 2009 [5]	F2 population derived from "Yellow Sarson C634" (early flowering Indian oilseed rape) cross with a DH line P11 of "Osome"(commercial variety of late floweing leafy vegetable)		unheated greenhouse	October 2007 to April 2008 in Sendai, Japan	Bolting Time in unheated greenhouse	DBOQTL1 (A02)	16	BrFLC2
					Bolting time in heated green house	DBOQTL1 (A02)	14	BrFLC2
						DBOQTL3 (A10)	19.9	BrFLC1
			heated greenhouse above 10℃		Budding Time in unheated greenhouse	DBUQTL1 (A02)	27.9	BrFLC2
					Budding Time in heated greenhouse	DBUQTL1 (A02)	21.7	BrFLC2

						DBUQTL2 (A10)	26.4	BrFLC1
					Flowering Time in unheated greenhouse	DFLQTL1(A02)	18.4	BrFLC2
					Flowering Time in heated greenhouse	DFLQTL1(A02)	18.1	BrFLC2
						DFLQTL2 (A10)	26.1	BrFLC1
Yuan et al, 2009 [6]	30 accessions with a wide range of flowering-time variation (19 DH lines and 11 inbred lines)	vernalized at 4 ℃ in the dark for 25 d	growth chamber at 25/20℃(day/night)	16 h	Average of flowering time from five individuals in the growth chamber and 15 individuals in the open field			SNP in intron 6 of BrFLC1
			grown in the open field.	transplanted on 21 March 2007				
Kakizaki et al, 2011 [7]	F2 populaiton derived from Chukanbohon Nou 6 gou (PL6, Chinese cabbage) cross with Chukanbohon Nou 7 gou (A9709, Chinese cabbage)	without vernalization	grown in the field	February to April 2009	Bolting Time	field-QTL1 (R10)	30.7	BrFLC1
						field-QTL2 (R03)	27.6	BrFLC5
Wu et al, 2012 [8]	159 *B.rapa* accessions		grown in the open field. The lowest mean daily temperature is 8.2℃	12 October 2009 to 1 April 2010.The day length vaired between 142.7 h in October and 244.8 h in April.	Days to flowering both in the open field and in the greenhouse			InDel across exon 4 and intron

			greenhouse 15-25℃	23 October 2010 to 20 March 2011.The shortest days are in winter (581.6 h in total during December to February), and the longest days are in spring (778.8 h in total from March to May).				4 of BrFLC2
Dong Xiao 2013 [9]	A *B. rapa* DH population DH68 of 163 DH lines was established from three F 1 plants of a cross between Yellow sarson YS-143 (acces- sion no. FIL500) as the female parent and Pak choi PC-175 (culti- var: Nai Bai Cai; accession no. VO2B0226) as the male parent.		Plants were grown in a heated greenhouse (18–21 °C) at Wageningen, The Netherlands. When the days were shorter than 16 h, artificial light was supplied until a photoperiod (200 μmol m –2 s –1) of 16 h.		Flowering time was defined as number of days from transplanting to appearance of the first open flower.	FLC1_A10.RL	18.6	FLC1_A10.RL
						FLC2_A02.RL	71.4	FLC2_A02.RL
						FLC3_A03.RL	22.7	FLC3_A03.RL
						FLC5_A03.RL	17.9	FLC5_A03.RL
Dong Xiao et al, 2013 [9]	DH population 68 derived from YS-143 (Yellow Sarson) cross with PC-175 (pak choi)	without vernalization	18-21℃	16 h	Flowering time	A02	33.5	BrFLC2
Kitamoto et al,	F2 population derived from	venalized at	25/18℃ (day/night)	16 h	Number of days to	R2	46	BrFLC2

2014 [10]	Tuskena No.2 (extremely late bloting non-heading leafy vegetable) cross with "Early"(early bolting Chinese cabbage, Sakata Co.)	4℃ with a 16 h photoperiod for 40 days			bolting	R3	9.9	BrFLC3
					Number of days to floweing	R2	43.6	BrFLC2
						R3	10.2	BrFLC3
					Number of leaves to flowering	R2	46.2	BrFLC2
						R3	7.7	BrFLC3
Su et al, 2018 [11]	A collection of 194 Chinese cabbage inbred lines, including 40 spring lines, 37 summer lines, and 117 autumn lines	vernalized at 4 ℃ under a long-day regime (16/8 h day/night) for 5 weeks.	greenhouse 25 ℃ /20℃ (day/night)	16 h	Bolting time	*ftA10.1*		BrFLC1
D. Xiao 2018 [12]	DH population 68 derived from YS-143 (Yellow Sarson) cross with PC-175 (pak choi)	08Sept-L	10-24℃/16hrs	16	Days to flowering	fQTL-3		BrFLC2
		09Apri-L	22-35℃/16-17hrs	16-17		fQTL-3		BrFLC2
		10Jan-L	10-24℃/16hrs	16		fQTL-3		BrFLC2
		10Feb-High-L	14-27℃/16hrs	16		fQTL-3		BrFLC2
		10Feb-High-S	14-27℃/8 hrs	8		fQTL-3		BrFLC2
		10Feb-Low-L	6-22℃/16 hrs	16		fQTL-3		BrFLC2
		10Feb-Low-S	6-22℃/8hrs	8		fQTL-3		BrFLC2
		11Mar-L	22-35℃/16-17hrs	16-17		fQTL-3		BrFLC2
		11Mar-S	22-35℃/8 hrs	8		fQTL-3		BrFLC2
		08Sept-L	10-24℃/16hrs	16	Days to flowering	fQTL-6		BrFLC5
		09Apri-L	22-35℃/16-17hrs	16-17		fQTL-6		BrFLC5
		10Jan-L	10-24℃/16hrs	16		fQTL-6		BrFLC5
		10Feb-High-L	14-27℃/16hrs	16		fQTL-6		BrFLC5
		10Feb-High-S	14-27℃/8 hrs	8		fQTL-6		BrFLC5

		10Feb-Low-L	6-22℃/16 hrs	16		fQTL-6		BrFLC5
		10Feb-Low-S	6-22℃/8hrs	8		fQTL-6		BrFLC5
		11Mar-L	22-35℃/16-17hrs	16-17		fQTL-6		BrFLC5
		11Mar-S	22-35℃/8 hrs	8		fQTL-6		BrFLC5

Supplementary Table 4. Primers used in this study

Pirmer name	Sequence 5'-3'
Vector construction primers	
AtJMJ18pro BamHI-F	GGATCCCGTTCAATTTACTCCTACTCTC
AtJMJ18pro SpeI-R	ACTAGTATGAATTGAAAAATCAATACTACTC
BrJMJ18CDSEcoRISpeI-F	GAATTCACTAGTATGGAGCATCTCAGTGTTGC
BrJMJ18CDSNCKpnI-R	GGTACCCATCAAATCCACTCCGAAAAG
JMJ18 CDSEcoRI-F	GGATCCGAATTCATGGAGCATCTCAGTG
JMJ18 CDSSacI-R	TCGACGGAGCTCCATCAAATCCACTCCGA
BrJMJ18 DT1-BsF	ATATATGGTCTCGATTGAAATGGACACCGTCTGTGGGTT
BrJMJ18 DT1-F0	TGAAATGGACACCGTCTGTGGGTTTTAGAGCTAGAAATAGC
BrJMJ18 DT2-R0	AACTTTCTTGTAAAACCCGCTTCAATCTCTTAGTCGACTCTAC
BrJMJ18 DT2-BsR	ATTATTGGTCTCGAAACTTTCTTGTAAAACCCGCTTCAA
qRT-PCR primers	
BrFLC1 RT-F	GCTGGAAGAGGAGAACCATGTTTTGGC
BrFLC1 RT-R	CTATAAAAATTCCACTCCCACTAACCCC
BrFLC2 RT-F	TCTGATGTAAGCGTCGACTCCCTCGTTC
BrFLC2 RT-R	GATTATTCTTCTCCATCTGGCTAGCCAAACCC
BrFLC3 RT-F	GTCGGTGGTGTAAGCGTGGACACCCTC
BrFLC3 RT-R	CTTCTTCTCCTTCTGGCCAGCCAAAGCC
BrFLC5 RT-F	AGAAATCAAGCGAATCGAGAAAAACAG
BrFLC5 RT-R	CTATCCCCGGAGGAGAAGCTGTAGAG
BrJMJ18NewRT-F	GGGAACTCTCAGCTTCTTCCGTTG
BrJMJ18NewRT-R	CACCAAGCTCAAAGACTCCCTAC
BraA09g029800-F	ATGGCGTCTCTTCAGCAAACCATGTTATC
BraA09g029800-R	GAGTATCGTTCCTCTTCGCAACATCTG
BraA04g028660-F	ATGTCAATCCTCCAGCTCTCTAGCTCTTC
BraA04g028660-R	CTCATAAGCCAAACCGGAGGTCTGTC
BraA07g034950-F	ATGGCACTCTCACGTCTCTCATCTCG
BraA07g034950-R	GGAAGAAGGAGAGGAGCTCTTGGG
BraA07g022160-F	ATGGAGACCAGCGTCATTAGCTACTC
BraA07g022160-R	CACCATTGTTCTTCGCCTTCGTGGC
BraA08g004460-F	CAACACCGTGCCGTTTTCCTGAAACCATG
BraA08g004460-R	GATAGGTTTCTGCTTGGTGGAAGCTGAAG
GADPH-F	CAGGTTTGGAATTGTCGAGG

GADPH-R	GAGCTGTGGAAGCACCTTTC
AtFLC RT-F	GGCTAGCCAGATGGAGAATAATCA
AtFLC RT-R	CCGCCGATTTAAGGTGGCTA
AtFT RT-F	CTGGAACAACCTTTGGCAAT
AtFT RT-R	TACACTGTTTGCCTGCCAAG
ACTIN2-F	GGTAACATTGTGCTCAGTGGTGG
ACTIN2-R	AACGACCTTAATCTTCATGCTGC'
ChIP PCR primers	
FLC_157_F	CGACAAGTCACCTTCTCCAAA
FLC_314_R	AGGGGGAACAAATGAAAACC
ACTIN_728_F	GATATTCAGCCACTTGTCTGTG
ACTIN_812_R	CTTACACATGTACAACAAAGAAGG
BrFLC1 CHIP 2-F	TCAGCTTTCCGTTCTCTGTGACGC
BrFLC1 CHIP 2-R	CCACACAAGATTCGCAGGAAGTATT
BrFLC2 CHIP 2-F	ATGGGAAGAAAGAAACTAGAGATCAAG
BrFLC2 CHIP 2-R	GAAGTTGTAAAGCTTGCCGGAGGCT
BrFLC3 CHIP 2-F	CTTTCTGTTCTCTGCGATGCATCCGTCG
BrFLC3 CHIP 2-R	CAGAAGCTTAAAAGGCTAATAAAG
BrFLC5 CHIP 2-F	ACAGTAGCAGACAAGTCACCTTCTGC
BrFLC5 CHIP 2-R	GAGGAGAAGCTGTAGAGCTTGTCGGA
BraA06g002250-F	CCGAGGAAGTTCAAGCTATCCTATTGCTAAG
BraA06g002250-R	CCACCAGACCTGGTAAACTTACCACTCTC
GADPH-F	CAGGTTTGGAATTGTCGAGG
GADPH-R	GAGCTGTGGAAGCACCTTTC

Reference

1. Yang, H., et al. Distinct phases of Polycomb silencing to hold epigenetic memory of cold in *Arabidopsis*. *Science* **357**, 1142-1145(2017).10.1126/science.aan1121
2. Cheng, F., et al. Subgenome parallel selection is associated with morphotype diversification and convergent crop domestication in *Brassica rapa* and *Brassica oleracea*. *Nature genetics* **48**, 1218-24(2016).10.1038/ng.3634
3. Lou, P., et al. Quantitative trait loci for glucosinolate accumulation in *Brassica rapa* leaves. The *New phytologist* **179**, 1017-1032(2008).10.1111/j.1469-8137.2008.02530.x
4. Zhao, J., et al. *BrFLC2* (*FLOWERING LOCUS C*) as a candidate gene for a vernalization response QTL in *Brassica rapa*. *Journal of experimental botany* **61**, 1817-25(2010).10.1093/jxb/erq048
5. Li, F., Kitashiba, H., Inaba, K.&Nishio, T. A *Brassica rapa* linkage map of EST-based SNP markers for identification of candidate genes controlling flowering time and leaf morphological traits. *DNA research*: *an international journal for rapid publication of reports on genes and genomes* **16**, 311-23(2009).10.1093/dnares/dsp020
6. Yuan, Y. X., et al. A naturally occurring splicing site mutation in the *Brassica rapa FLC1* gene is associated with variation in flowering time. *Journal of experimental botany* **60**, 1299-308(2009).10.1093/jxb/erp010
7. Kakizaki, T., et al. Identification of quantitative trait loci controlling late bolting in Chinese cabbage (*Brassica rapa L.*) parental line Nou 6 gou. *Breeding Science* **61**, 151-159(2011).10.1270/jsbbs.61.151
8. Wu, J., et al. A naturally occurring InDel variation in *BraA.FLC.b* (*BrFLC2*) associated with flowering time variation in *Brassica rapa*. *BMC plant biology* **12**, 151(2012).10.1186/1471-2229-12-151
9. Xiao, D., et al. The *Brassica rapa FLC* homologue *FLC2* is a key regulator of flowering time, identified through transcriptional co-expression networks. *Journal of experimental botany* **64**, 4503-16(2013).10.1093/jxb/ert264
10. Kitamoto, N., Yui, S., Nishikawa, K., Takahata, Y.&Yokoi, S. A naturally occurring long insertion in the first intron in the *Brassica rapa FLC2* gene causes delayed bolting. *Euphytica* **196**, 213-223(2014).10.1007/s10681-013-1025-9
11. Su, T., et al. A Genomic Variation Map Provides Insights into the Genetic Basis of Spring Chinese Cabbage (*Brassica rapa ssp. pekinensis*) Selection. *Molecular plant* **11**, 1360-1376(2018).10.1016/j.molp.2018.08.006
12. Xiao, D., et al. Genetic dissection of flowering time in *Brassica rapa* responses to temperature and photoperiod. *Plant science: an international journal of experimental plant biology* **280**, 110-119(2019).10.1016/j.plantsci.2018.10.027
