# Supplementary material for: Temperature-dependent jumonji demethylase modulates flowering time by targeting H3K36me2/3 in Brassica rapa
Source: Nat Commun. 2024 Jun 28;15:5470. doi: 10.1038/s41467-024-49721-z (PMC11211497; doi:10.1038/s41467-024-49721-z)
Supplement: Supplementary file 3 — Description of additional supplementary files [file 41467_2024_49721_MOESM3_ESM.pdf]

Description of Additional Supplementary Files

File name: Supplementary Data 1

Description: Genetic regions and candidate gene list underlying selection of *DG* vs. *PC* and *Par* vs. *DG*, respectively.

s1 Overlapping regions of FST (top 5%) and compared π (50%) analysis of DG/PC.

s2 Genes within putative selective sweeps of DG from PC.

s3 Overlapping regions of FST (top 5%) and compared π (50%) analysis of Par/PC.

s4 Genes within putative selective sweeps of Par from DG.

s5 Differently expressed genes during (DEGs) floral transition in the selected regions of DG/PC

s6 Differently expressed genes during (DEGs) floral transition in the selected regions of Par/DG

File name: Supplementary Data 2

Description: Enriched Gene Ontology (GO) categories of the selected genes in Supplementary Data 1, s1, and s3.

s1 A total of 171 GO terms were specifically enriched in the *DG*/*PC* comparison.

s2 A total of 45 GO terms were specifically enriched in the *Par*/*DG* comparison.

File name: Supplementary Data 3

Description: The selective loci and gene list which were specially differentiates between *Par* vs. *DG* but not *DG* vs. *PC*.

s1 The selective loci specially differentiates between *Par*/*DG* but not *DG*/*PC*.

s2 The selective genes which were specially differentiates between *Par*/*DG* but not *DG*/*PC*.

File name: Supplementary Data 4

Description: Flowering time QTL(HS)s which specially respond to high temperature

File name: Supplementary Data 5

Description: Differentially expressed genes (DEGs) in the RNA sequencing data of *PC*, *DG* and *Par* planted under natural field.

s1 DEGs of *PC* planted under natrual field conditions in Beijing

s2 DEGs of *DG* planted under natrual field conditions in Beijing

s3 DEGs of *Par* planted under natrual field conditions in Beijing

s4 Expression patterns of the three candidates BrJMJs in gQTLA09-1 of *PC*, *DG* and *Par* before and during bolting under natural field conditions in Beijing.

s5 Mapping information of the RNA sequencing samples

File name: Supplementary Data 6

Description: Candidate BrJMJ18 binding genes screened by ChIP-seq and RNA-seq.

File name: Supplementary Data 7

Description: Genes enriched in anti-GFP ChIP-seq using BrJMJ18-OX *Par* plants under NC and HT conditions.

s1 Genes enriched in BrJMJ18^{PC}-OX *Par* plants under NC condition.

s2 Genes enriched in BrJMJ18^{Par}-OX *Par* plants under NC condition.

s3 Genes enriched in BrJMJ18^{PC}-OX *Par* plants under HT condition.

s4 Genes enriched in BrJMJ18^{Par}-OX *Par* plants under HT condition.

s5 Data quality

File name: Supplementary Data 8

Description: Differentially expressed genes (DEGs) in the RNA sequencing data of BrJMJ18^{PC}-OX and BrJMJ18^{Par}-OX *Par* plants.

s1 DEGs in BrJMJ18^{PC}-OX *Par* plants under NC conditions

s2 DEGs in BrJMJ18^{Par}-OX *Par* plants under NC conditions

s3 DEGs in in BrJMJ18^{PC}-OX *Par* plants under HS conditions

s4 DEGs in BrJMJ18^{Par}-OX *Par* plants under HS conditions

s5 Mapping information
