# Supplementary material for: Temperature-dependent jumonji demethylase modulates flowering time by targeting H3K36me2/3 in Brassica rapa
Source: Nat Commun. 2024 Jun 28;15:5470. doi: 10.1038/s41467-024-49721-z (PMC11211497; doi:10.1038/s41467-024-49721-z)
Supplement: Supplementary file 12 — Reporting Summary [file 41467_2024_49721_MOESM12_ESM.pdf]

nature portfolio

Corresponding author(s): Fenglan Zhang, Shuancang Yu and Tongbing Su

Last updated by author(s): Jun 6, 2024

Reporting Summary

Nature Portfolio wishes to improve the reproducibility of the work that we publish. This form provides structure for consistency and transparency in reporting. For further information on Nature Portfolio policies, see our Editorial Policies and the Editorial Policy Checklist.

Statistics

For all statistical analyses, confirm that the following items are present in the figure legend, table legend, main text, or Methods section.

n/a	Confirmed	
☐	☒	The exact sample size (*n*) for each experimental group/condition, given as a discrete number and unit of measurement
☐	☒	A statement on whether measurements were taken from distinct samples or whether the same sample was measured repeatedly
☐	☒	The statistical test(s) used AND whether they are one- or two-sided<br>*Only common tests should be described solely by name; describe more complex techniques in the Methods section.*
☒	☐	A description of all covariates tested
☒	☐	A description of any assumptions or corrections, such as tests of normality and adjustment for multiple comparisons
☐	☒	A full description of the statistical parameters including central tendency (e.g. means) or other basic estimates (e.g. regression coefficient) AND variation (e.g. standard deviation) or associated estimates of uncertainty (e.g. confidence intervals)
☐	☒	For null hypothesis testing, the test statistic (e.g. *F*, *t*, *r*) with confidence intervals, effect sizes, degrees of freedom and *P* value noted<br>*Give P values as exact values whenever suitable.*
☒	☐	For Bayesian analysis, information on the choice of priors and Markov chain Monte Carlo settings
☒	☐	For hierarchical and complex designs, identification of the appropriate level for tests and full reporting of outcomes
☒	☐	Estimates of effect sizes (e.g. Cohen's *d*, Pearson's *r*), indicating how they were calculated

Our web collection on statistics for biologists contains articles on many of the points above.

Software and code

Policy information about availability of computer code

Data collection	Zeiss Zen software (v 14.0.0.201) were used to acquire images. LightCycler 480 software were used to acquire qRT-PCR data.
Data analysis	Pod and hypocotyl length of Arabidopsis and Par plants were photographed and measured using Image J software (https://imagej.net/ij/download.html). Adobe Photoshop (v CS5) was used to crop and combine images. Plants penotype and qRT-PCR data were analyzed using Microsoft excel (2013). Bar graphs and box-and-whisker plots were generated using OriginPro 2022 (https://www.originlab.com/). Data processing, SNP calling, population genetic analyses and gene flow estimates were conducted as described in Su et al (2018). Filtered reads were mapped to the reference genome using the "mem" algorithm of the Burrows-Wheeler Aligner (version 0.7.5a-r405). The mapping results were processed by sorting and duplicate marking using functions in SAMTOOLS (Li et al., 2009) and PICARD (http://broadinstitute.github.io/picard/), local realigment around indels was performed using Indel-Realigner in GATK (McKenna et al., 2010) and HaplotypeCalller in GATK was used to call SNPs and small InDels (Insetion/Deletions). The phylogenetic tree visualizing, editing and root assigning were performed using ITOL (http://itol.embl.de/). The software PopLDdecay (https://github.com/BGI-shenzhen/PopLDdecay) was used to calculate linkage disequilibrium (LD). FST was calculated using VCFtools (v 0.1.13).

For manuscripts utilizing custom algorithms or software that are central to the research but not yet described in published literature, software must be made available to editors and reviewers. We strongly encourage code deposition in a community repository (e.g. GitHub). See the Nature Portfolio guidelines for submitting code & software for further information.

Data

Policy information about availability of data

All manuscripts must include a data availability statement. This statement should provide the following information, where applicable:

- Accession codes, unique identifiers, or web links for publicly available datasets
- A description of any restrictions on data availability
- For clinical datasets or third party data, please ensure that the statement adheres to our policy

The ChiP-seq data is accessible on NCBI under the GEO accession number GSE223969. All the raw sequencing data are archived at Genome Sequence Archive of China National Center for Bioinformation (https://www.cncb.ac.cn/) with the accession number PRJCA025632.

Research involving human participants, their data, or biological material

Policy information about studies with human participants or human data. See also policy information about sex, gender (identity/presentation), and sexual orientation and race, ethnicity and racism.

Reporting on sex and gender	N/A
Reporting on race, ethnicity, or other socially relevant groupings	N/A
Population characteristics	N/A
Recruitment	N/A
Ethics oversight	N/A

Note that full information on the approval of the study protocol must also be provided in the manuscript.

Field-specific reporting

Please select the one below that is the best fit for your research. If you are not sure, read the appropriate sections before making your selection.

☒ Life sciences ☐ Behavioural & social sciences ☐ Ecological, evolutionary & environmental sciences

For a reference copy of the document with all sections, see nature.com/documents/nr-reporting-summary-flat.pdf

Life sciences study design

All studies must disclose on these points even when the disclosure is negative.

Sample size	For phenotyping of Arabidopsis and Par plants, 15 individual plants of each line were measured. For tobacco leaf transient transformation experiment, images of at least 30 epidermis cells expressing each allele of BrJMJ18-GFP protein under NC or HS condition were acquired using the confocal laser scanning system Zeiss LSM510, respectively. BrJMJ18-GFP protein was localized in the nucleus without exception. Sample size was chosen based on previous experience and standards in the field.
Data exclusions	Data were not excluded from analysis.
Replication	The phenotyping and transient transformation experiments were repeated for three times. All attempts of replication were successful and gave similar results.
Randomization	All planted arabidopsis and Par plants were used in the phenotyping experiment. And the epidermis cell were selected randomly in the tobacco transient tranformation experiment.
Blinding	The persons who performing the phenotyping and transient transformation experiments were unaware of the sample identity. These data was analyzed in a double-blind approach.

Reporting for specific materials, systems and methods

We require information from authors about some types of materials, experimental systems and methods used in many studies. Here, indicate whether each material, system or method listed is relevant to your study. If you are not sure if a list item applies to your research, read the appropriate section before selecting a response.

Materials & experimental systems

n/a	Involved in the study
☐	☒ Antibodies
☒	☐ Eukaryotic cell lines
☒	☐ Palaeontology and archaeology
☒	☐ Animals and other organisms
☒	☐ Clinical data
☒	☐ Dual use research of concern
☐	☒ Plants

Methods

n/a	Involved in the study
☐	☒ ChIP-seq
☒	☐ Flow cytometry
☒	☐ MRI-based neuroimaging

Antibodies

Antibodies used

Antibodies against the following proteins were used: GFP (HT801-01, TransGen Biotech, Beijing, China), β-Tubulin (HC101, TransGen Biotech, Beijing, China), Histone H3 mono/di/tri methyl K4 (ab8895/ab32356/ab213224, Abcam, Cambridge, MA, USA), Histone H3 mono/di/tri methyl K9 (ab176880/ab32521/ab176916, Abcam, Cambridge, MA, USA), Histone H3 Histone H3 mono/di/tri methyl K27 (ab194688/ab24684/ab195477, Abcam, Cambridge, MA, USA), and Histone H3 mono/di/tri methyl K36 (ab176920/ab176921/ ab195489, Abcam, Cambridge, MA, USA). Anti-BrJMJ18 antibodies were produced by immunizing rabbits with a recombinantly expressed BrJMJ18 protein fragment (amino acids 1–133) expressed in Escherichia coli and purified using an BrJMJ18 antigen column (HuaBio, Hangzhou, China). IRDye 680RD Goat anti-mouse (926-68070, LI-COR, Lincoln, NE, USA) or anti rabbit (926-68071, LI-COR, Lincoln, NE, USA)) antibodies were used as secondary antibodies.

Validation

Antibodies from TransGen Biotech and Abcam have been validated by the manufacturers by immunoblotting. Secondary antibodies from LI-COR have been validated by the manufacturer by dot blot and and/or solid-phase adsorbed for minimal cross-reactivity with human, rabbit, goat, rat, and horse serum proteins. Anti-BrJMJ18 antibody has been validated by HuaBio company and us by immunobloting.

Plants

Seed stocks

Arabidopsis Col-0 seeds were obtained from the European Arabidoposis stock center. A collection of 210 varieties of different B. rapa morphotypes were used for genetic structure and selection analyses. These inbred Brassica rapa lines were collected by the co-authors from all over the world and self-pollinated for at least six generations. The origin locations were listed in Table S1. The inbred Brassica rapa lines used for F2 population generation, high temperature treatment and transgenic plants construction are all included in our germplasm collection.

Novel plant genotypes

The Arabidopsis transgenic plants AtBrJMJ18::BrJMJ18:gfp was generated the flower-dipping method. Two independent lines of each BrJMJ18 alleles were used for analysis on T2 generation. The subsp. chinensis var. parachinensis (Par) BrJMJ18 overexpression and gene edited plants were generated by Agrobacterium-mediated transformation using cotyledonary petiole as explant. For 35S::BrJMJ18:gfp transgenic Par plants, one independent line of each BrJMJ18 alleles was used for analysis on T1 generation. The BrJMJ18 gene-edited plant was generated through CRISPR/Cas9 system. Guide RNAs (sgRNA) "GAAATGGACACCGTCTGTGG" and "GAAGCGGGTTTTACAAGAAA" were inserted into pKSE401.

Authentication

The Arabidopsis and Par BrJMJ18 transgenic plants were screened using 15 mg/L hygromycin or 25 mg/L kanamycin. Transgene expression was detected by immunoblotting using anti-GFP antibody (HT801-01, TransGene, Beijing, China). For the Par BrJMJ18 CRISPR/Cas9 plants, the target gene BrJMJ18 from T0 plants was amplified, purified, and cloned into a vector using the pMD™18-T Vector Cloning Kit (Takara) for sequencing.

ChIP-seq

Data deposition

☒ Confirm that both raw and final processed data have been deposited in a public database such as GEO.

☒ Confirm that you have deposited or provided access to graph files (e.g. BED files) for the called peaks.

Data access links
May remain private before publication.

https://www.ncbi.nlm.nih.gov/geo/query/acc.cgi?acc=GSE223969

Files in database submission

GSM7008932 HCK Input; GSM7008933 LCK Input; GSM7008934 LH1 Input; GSM7008935 LH2 Input; GSM7008936 HH1 Input; GSM7008937 HH2 Input; GSM7008937 HH2 Input; GSM7008939 LCK IP; GSM7008940 LH1 IP; GSM7008941 LH2 IP; GSM7008942 HH1 IP; GSM7008943 HH2 IP. "L" represents samples were treated with NC (22?/22?, 16h/8h). "H" represents samples were treated with HS (29?/29?, 16h/8h). "CK" r represents wild type Par control. "H1" represents BrJMJ18PC-OX plants. "H2" represents BrJMJ18Par-OX plants.

Genome browser session
(e.g. UCSC)

http://genome.ucsc.edu/cgi-bin/hgTracks?hgsid=1718034740_Vkl1DrmRiOa4mUuEAdbYguIYMniJ

Methodology

Replicates

Two biological replicates were performed for each sample.

Sequencing depth

For ChIP-seq using anti-GFP antibody, the total clean reads of the Immunoprecipitated sample were 23,943,076-2,929,666. Clean bases were 4.9-3.52G. 58.32%-72.55% reads were mapped to the reference genome. Unique reads were 24.82%-29.01. And the reads were paired-end. For ChIP-seq using anti-GFP antibody, the total clean reads of Immunoprecipitated samples were 40,519,410

and 43,068,060, containing 5996225762 and 6352462960 clean bases, respectively. 65.3% and 76.59% of the reads were mapped to the reference genome. 481948 and 712279 unique reads were found. The reads were also paired-end.

Antibodies	Anti-GFP antibody (ab290, Abcam). Anti-BrJMJ18 antibody (HuaBio, Hangzhou, China).
Peak calling parameters	ChIP-seq experiment was carried out by IGENEBOOK Biotechnology Company (Wuhan, China) according to a previously described method (Landt et al., 2012). All parameters used are default without modification. We will confirm again with the company.
Data quality	Data quality is evaluated in accordance with the company's standard process. The square (R2) of the Pearson correlation coefficient of the two biological repeats was greater than 0.8. We will contact the company for more details. For the ChIP-seq with anti-GFP antibody, about 1,000 and 4,500 peaks were at FDR 5% in the samples treated with NC and HS, respectively. For the ChIP-seq with anti-BrJMJ8 antibody, about 2,000 peaks were at FDR 5%.
Software	Immunoprecipitated DNA was used to construct sequencing libraries following the protocol provided by the I NEXTFLEX® ChIP Seq Library Prep Kit for Illumina® Sequencing (NOVA-5143-02, Bioo Scientific, Austin, TX, USA) and sequenced on Illumina Xten with the PE 150 method (Illumina Inc. San Diego, CA, USA). Trimmomatic (version 0.38) was used to filter out low-quality reads (Bolger et al., 2014). Clean reads were mapped to the B. rapa genome v3.0 using Bwa (version 0.7.15) (Li and Durbin, 2009). Samtools (version 1.3.1) was used to remove potential PCR duplicates (Li et al., 2009). MACS2 software (version 2.1.1.20160309) was used to call peaks using default parameters (bandwidth, 300 bp; model fold, 5, 50; q value, 0.05). If the summit of a peak located closest to the transcription start site (TSS) of one gene, the peak was assigned to that gene (Salmon-Divon et al., 2010). Gene Ontology (GO) enrichment analysis was performed using the EasyGO gene ontology enrichment analysis tool (http://bioinformatics.cau.edu.cn/easygo/) (Zhou and Su, 2007). The GO term enrichment was calculated using hypergeometric distribution, with a P value cutoff of 0.01. P values obtained by Fisher's exact test were adjusted by the false discovery rate (FDR) for multiple comparisons to detect overrepresented GO terms.
