# Supplementary figures and images for: Temperature-dependent jumonji demethylase modulates flowering time by targeting H3K36me2/3 in Brassica rapa
Source: Nat Commun. 2024 Jun 28;15:5470. doi: 10.1038/s41467-024-49721-z (PMC11211497; doi:10.1038/s41467-024-49721-z)

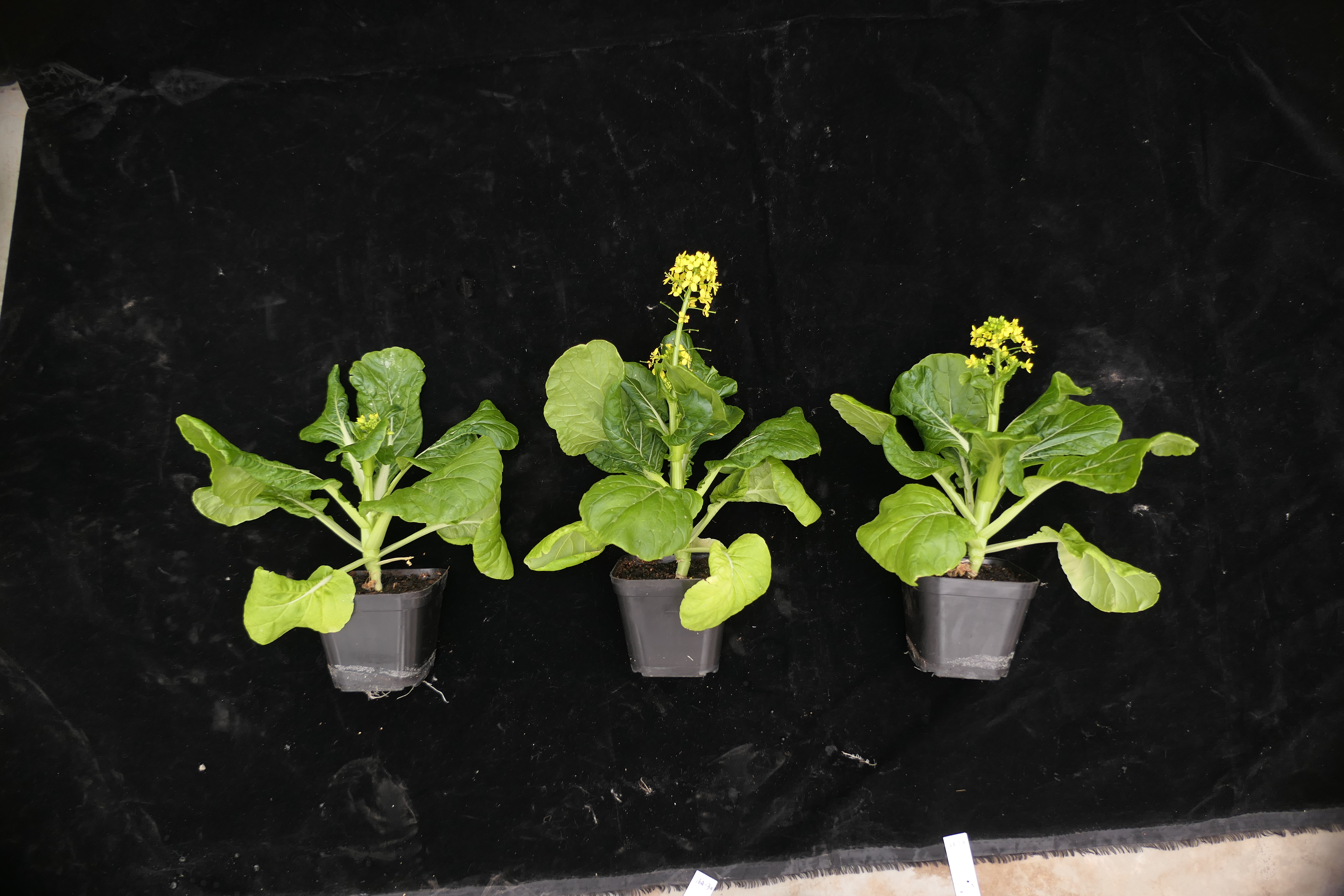

Supplement: Supplementary file 13 — Source Data [file 41467_2024_49721_MOESM13_ESM.zip › 406988_4_data_set_9156724_sddqhm/Source data-Supplementary Dataset/Fig3A.JPG]

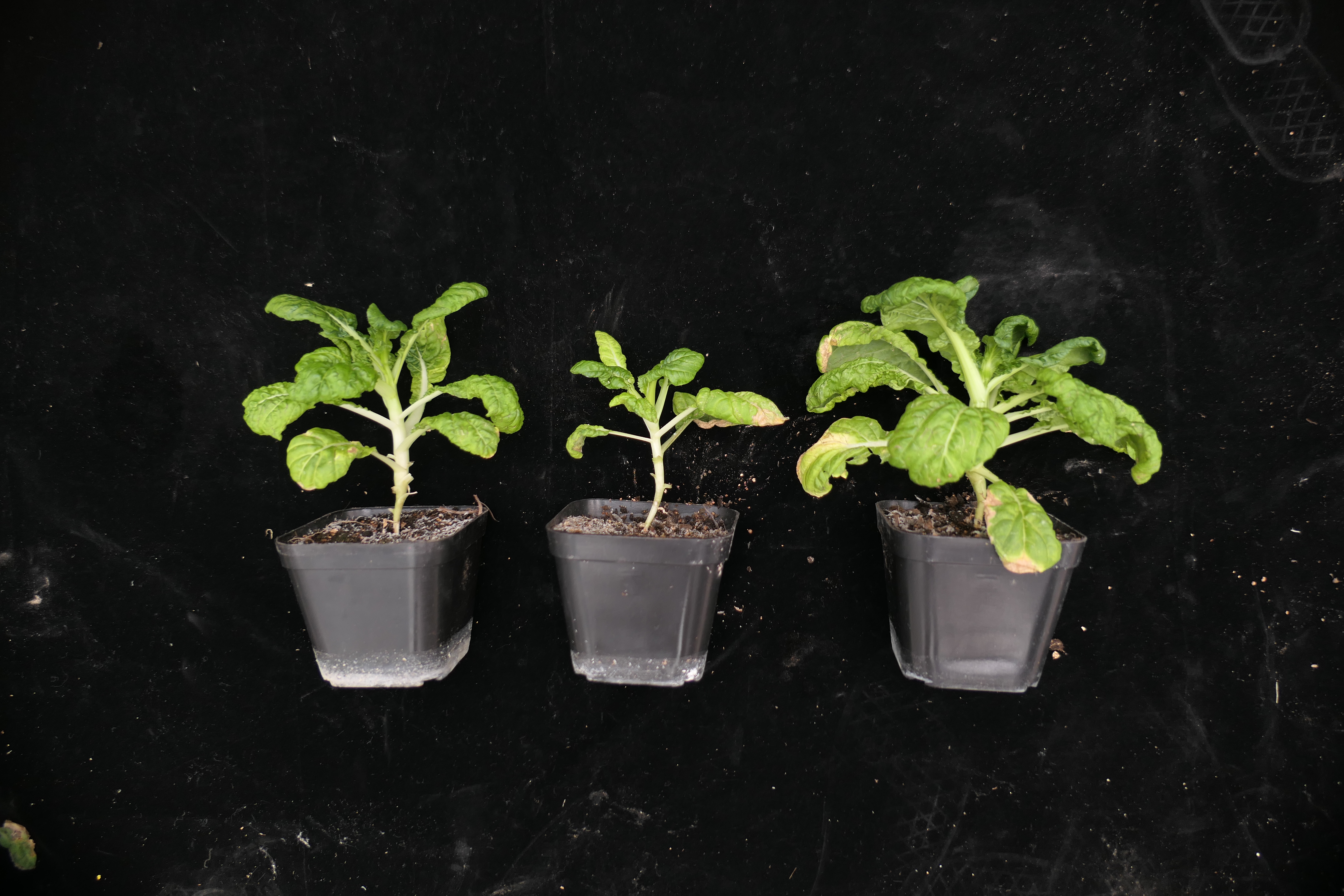

Supplement: Supplementary file 13 — Source Data [file 41467_2024_49721_MOESM13_ESM.zip › 406988_4_data_set_9156724_sddqhm/Source data-Supplementary Dataset/Fig3B.JPG]

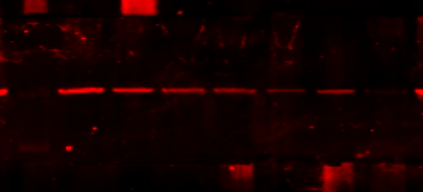

Supplement: Supplementary file 13 — Source Data [file 41467_2024_49721_MOESM13_ESM.zip › 406988_4_data_set_9156724_sddqhm/Source data-Supplementary Dataset/Fig3D-1.png]

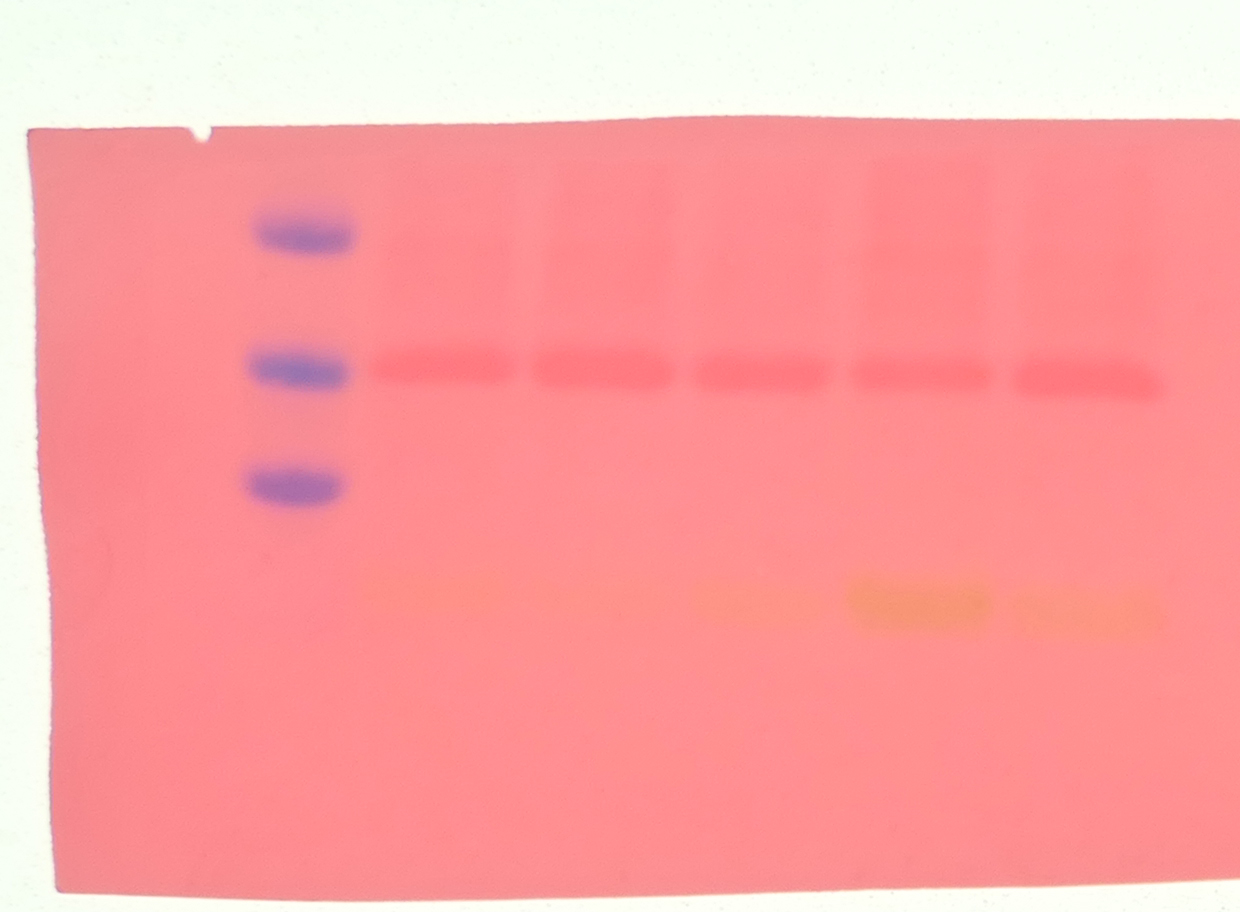

Supplement: Supplementary file 13 — Source Data [file 41467_2024_49721_MOESM13_ESM.zip › 406988_4_data_set_9156724_sddqhm/Source data-Supplementary Dataset/Fig3D-2.jpg]

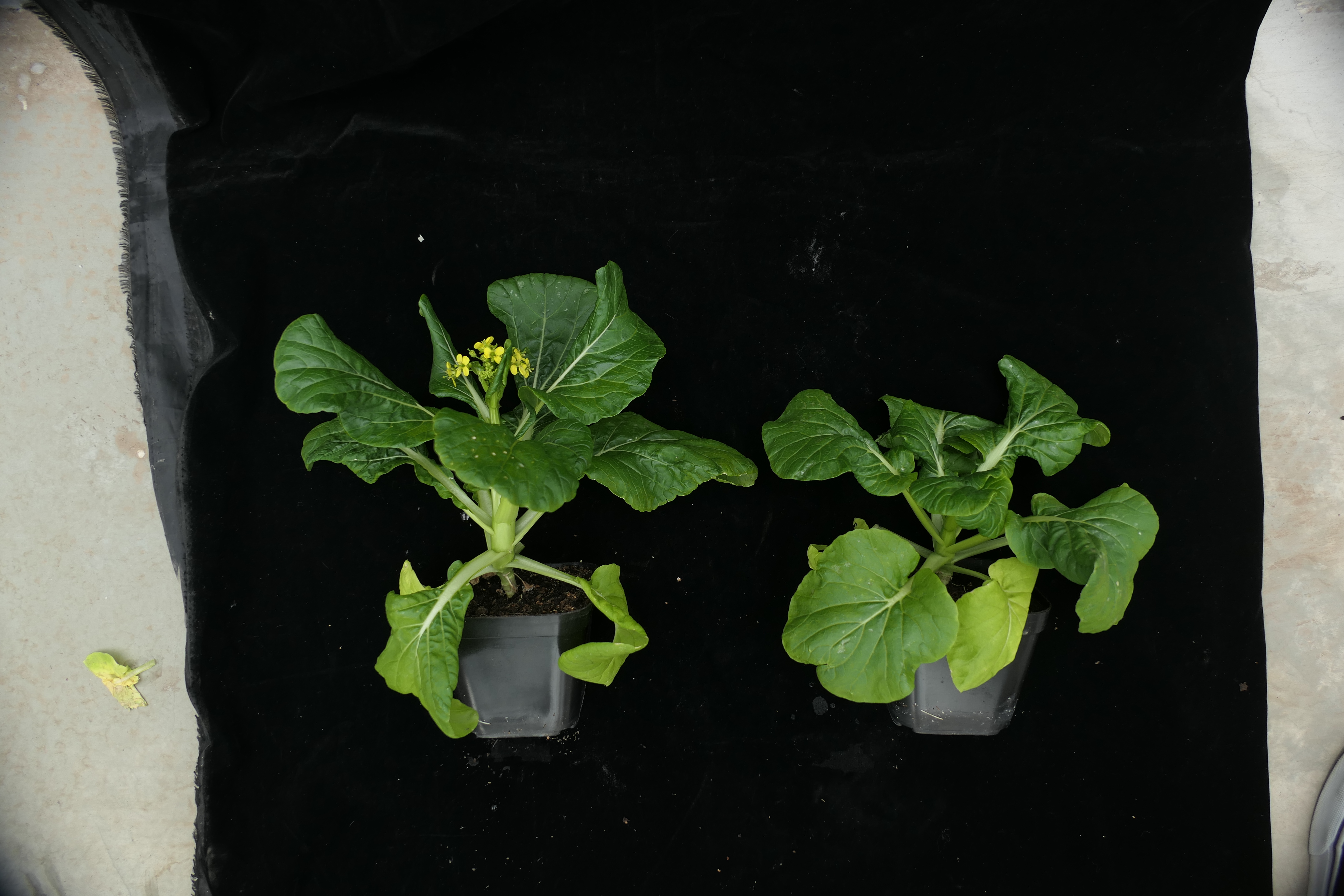

Supplement: Supplementary file 13 — Source Data [file 41467_2024_49721_MOESM13_ESM.zip › 406988_4_data_set_9156724_sddqhm/Source data-Supplementary Dataset/Fig3E.JPG]

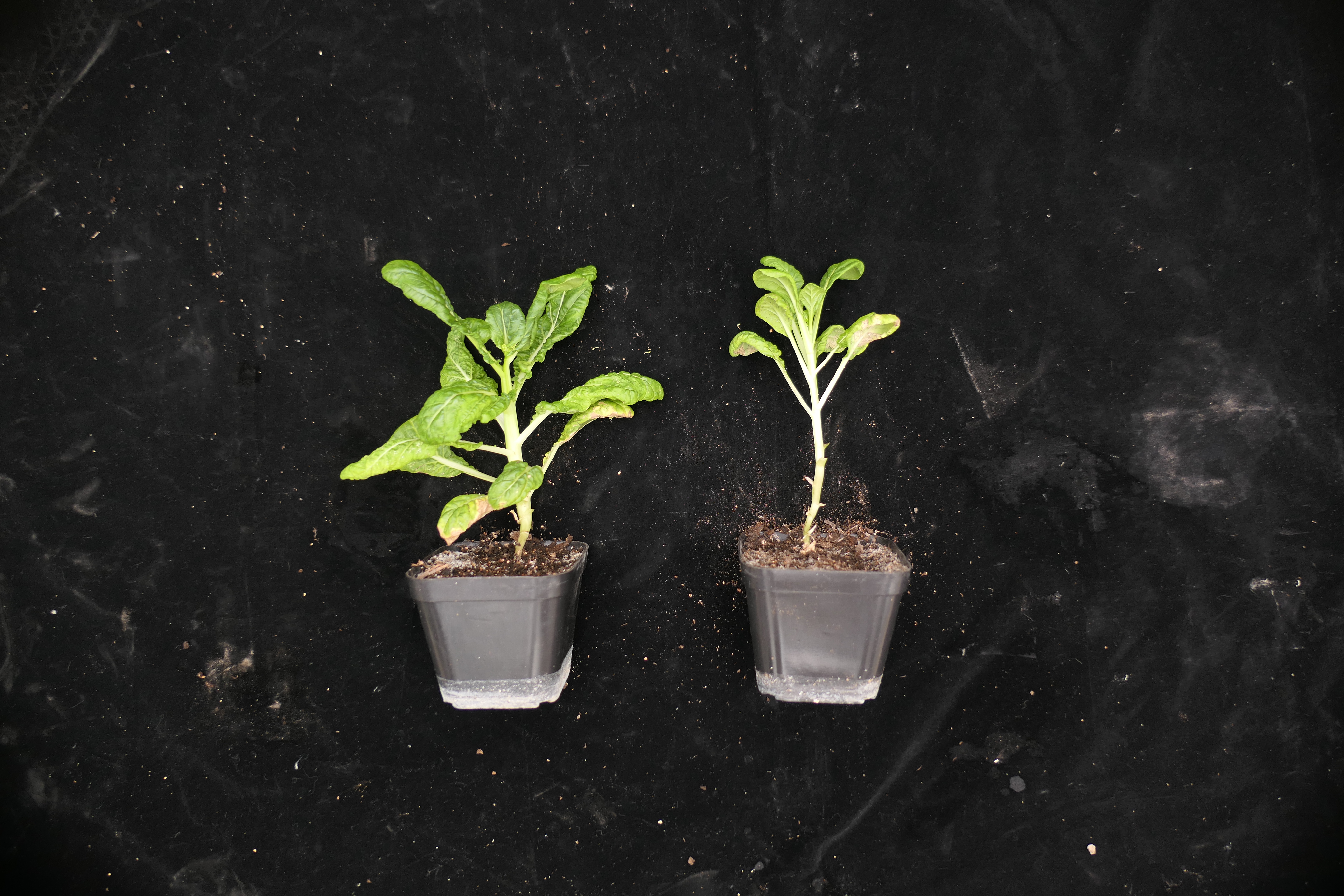

Supplement: Supplementary file 13 — Source Data [file 41467_2024_49721_MOESM13_ESM.zip › 406988_4_data_set_9156724_sddqhm/Source data-Supplementary Dataset/Fig3F.JPG]

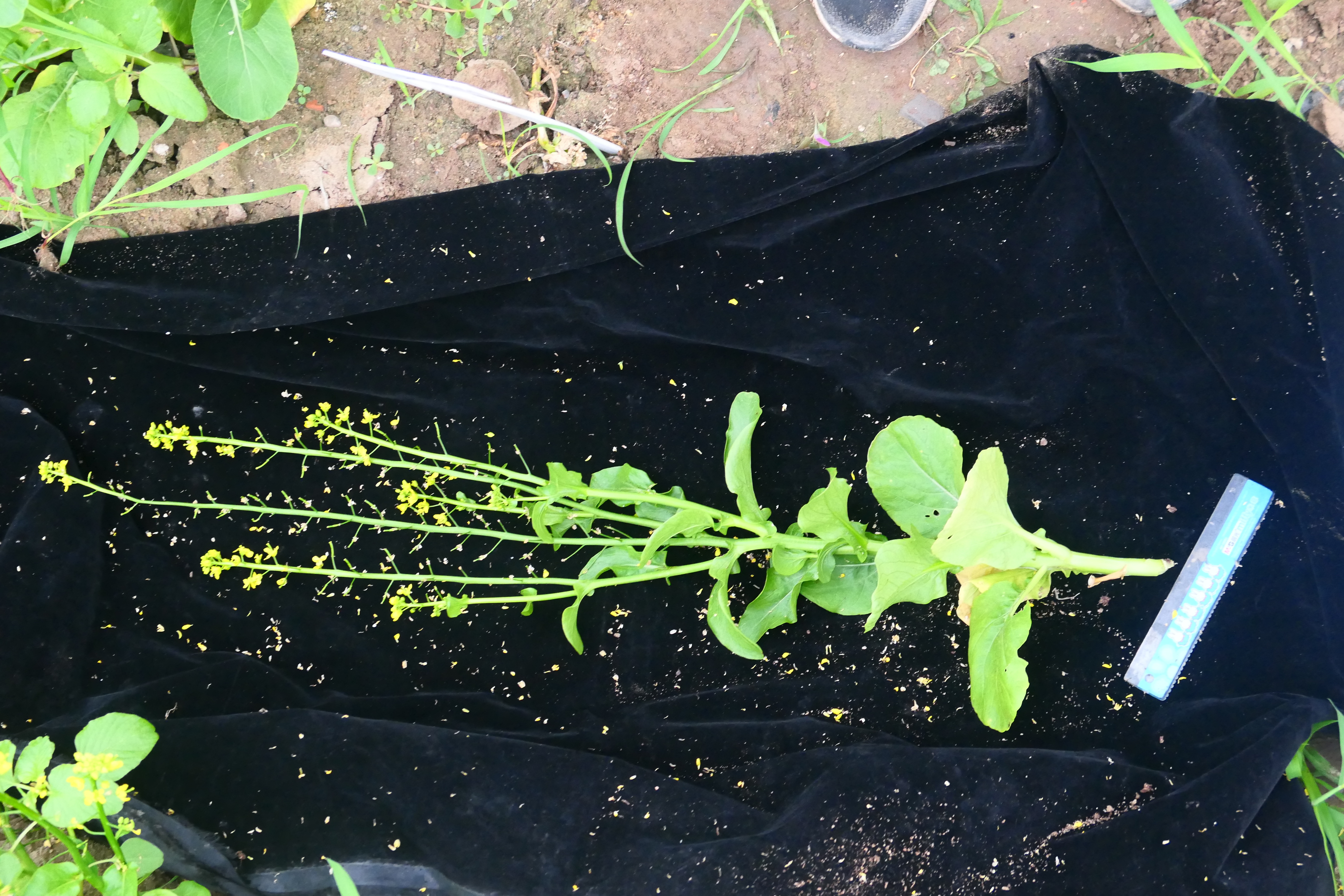

Supplement: Supplementary file 13 — Source Data [file 41467_2024_49721_MOESM13_ESM.zip › 406988_4_data_set_9156724_sddqhm/Source data-Supplementary Dataset/Fig3I/BrJMJ18CR.JPG]

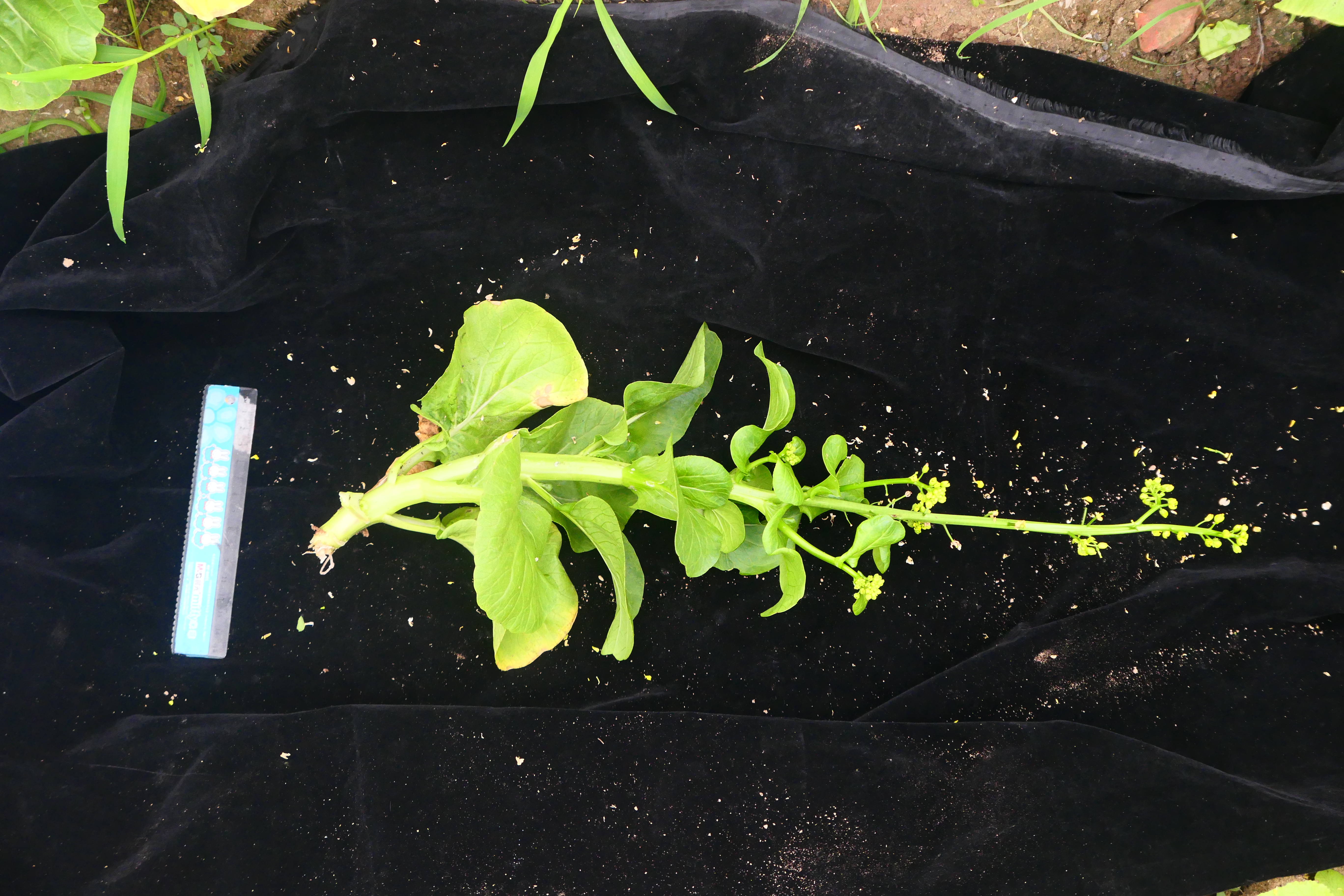

Supplement: Supplementary file 13 — Source Data [file 41467_2024_49721_MOESM13_ESM.zip › 406988_4_data_set_9156724_sddqhm/Source data-Supplementary Dataset/Fig3I/BrJMJ18Par.JPG]

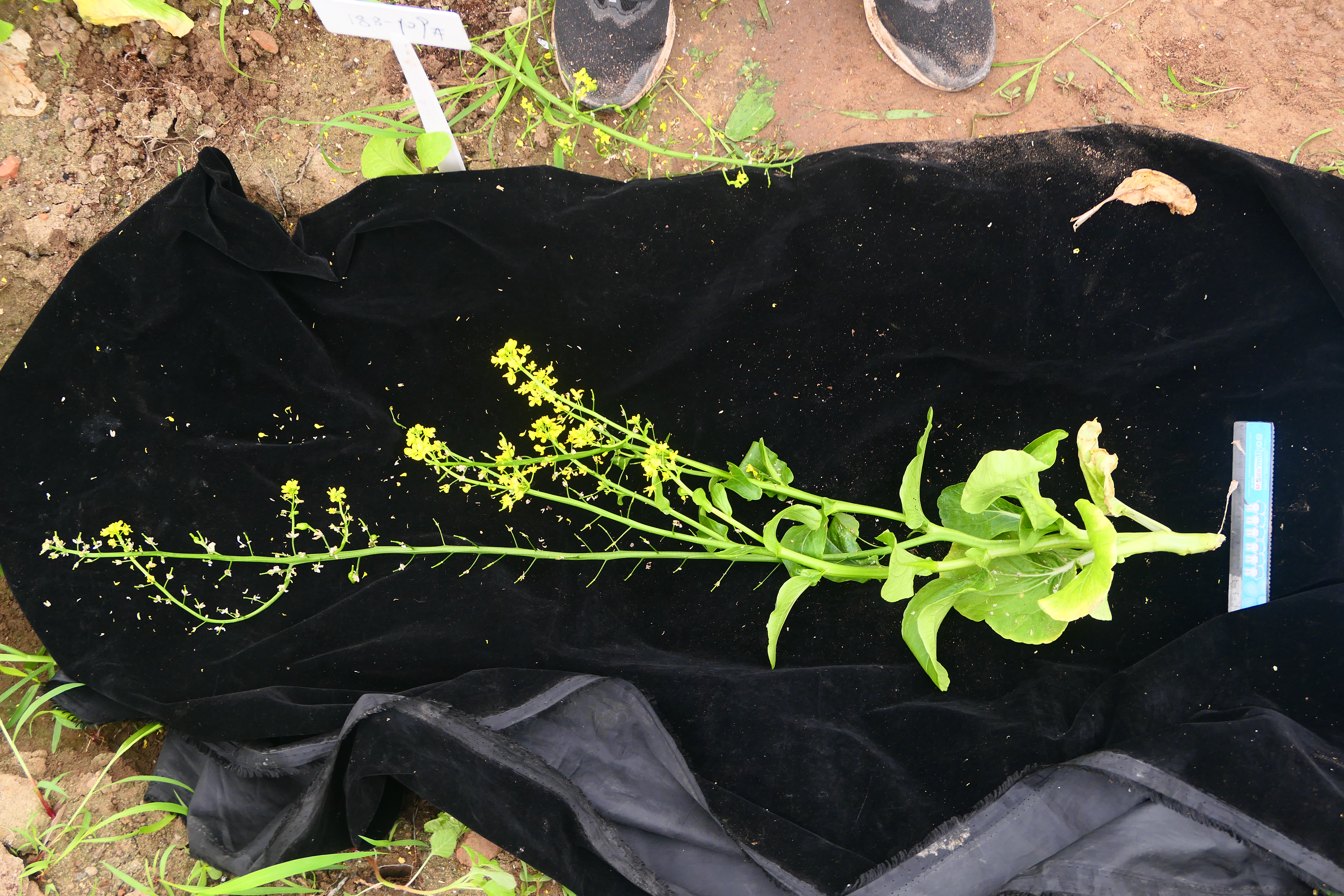

Supplement: Supplementary file 13 — Source Data [file 41467_2024_49721_MOESM13_ESM.zip › 406988_4_data_set_9156724_sddqhm/Source data-Supplementary Dataset/Fig3I/BrJMJ18PC.JPG]

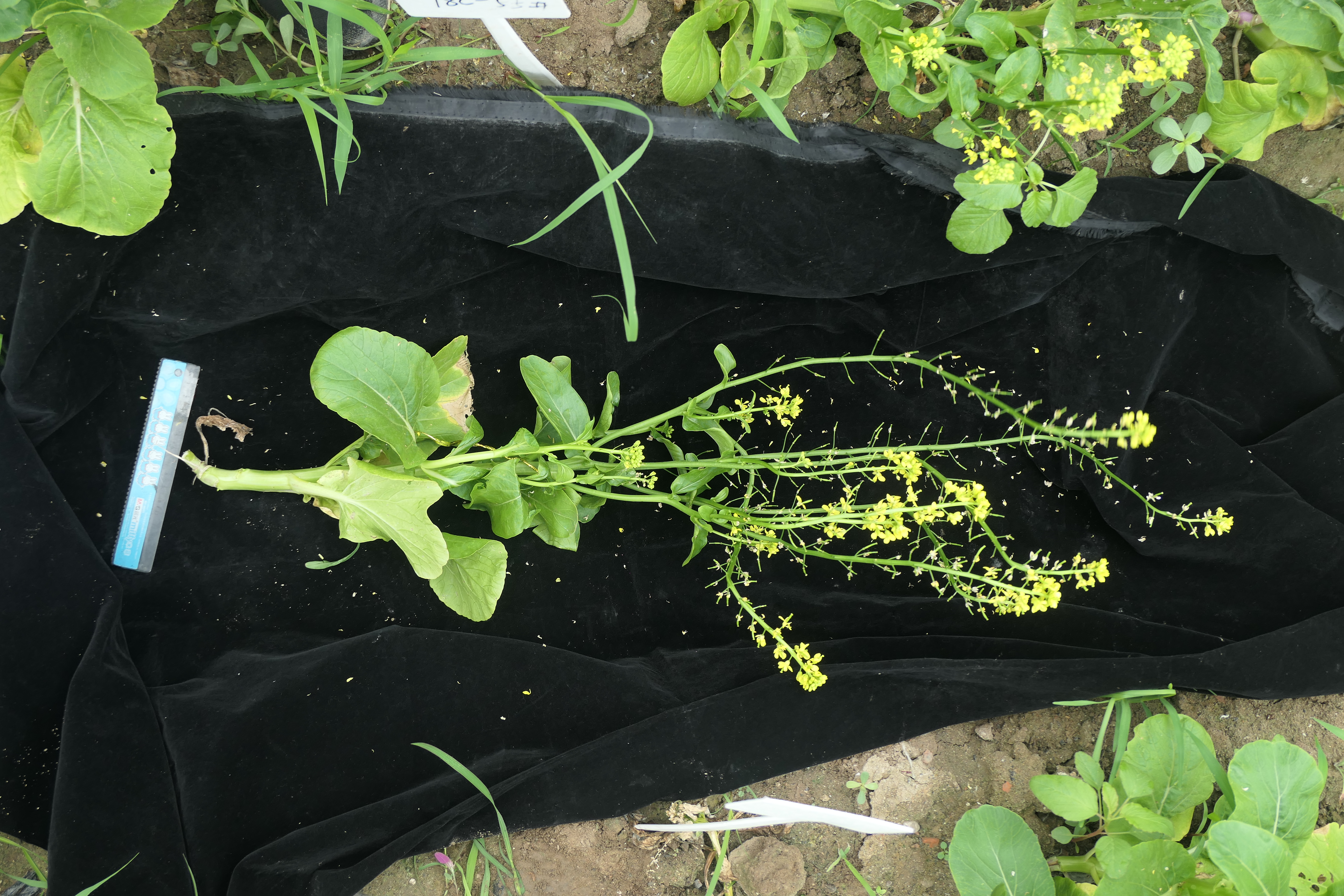

Supplement: Supplementary file 13 — Source Data [file 41467_2024_49721_MOESM13_ESM.zip › 406988_4_data_set_9156724_sddqhm/Source data-Supplementary Dataset/Fig3I/Par.JPG]

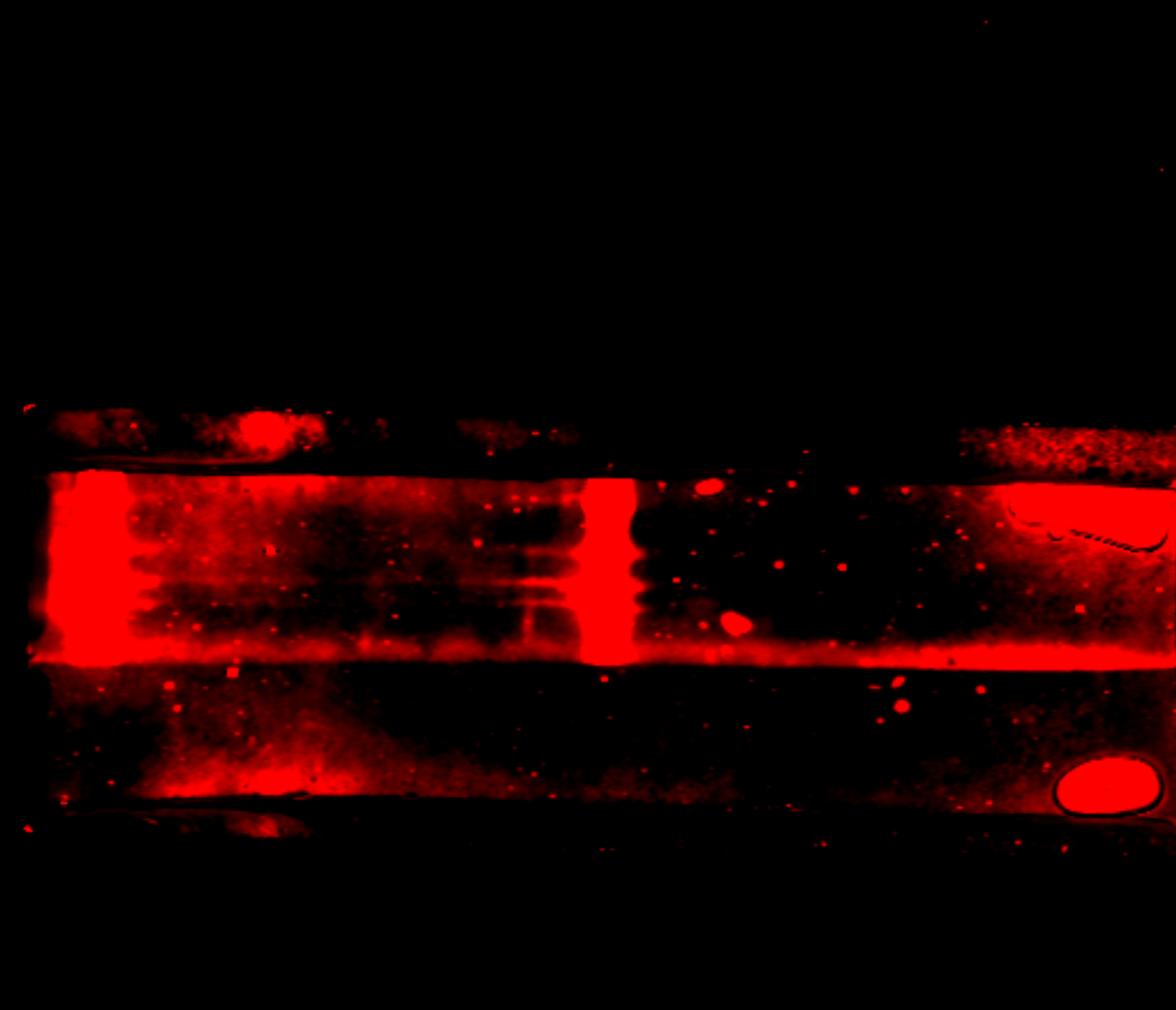

Supplement: Supplementary file 13 — Source Data [file 41467_2024_49721_MOESM13_ESM.zip › 406988_4_data_set_9156724_sddqhm/Source data-Supplementary Dataset/Fig4A and S14A/Anti-H3K362.tif]

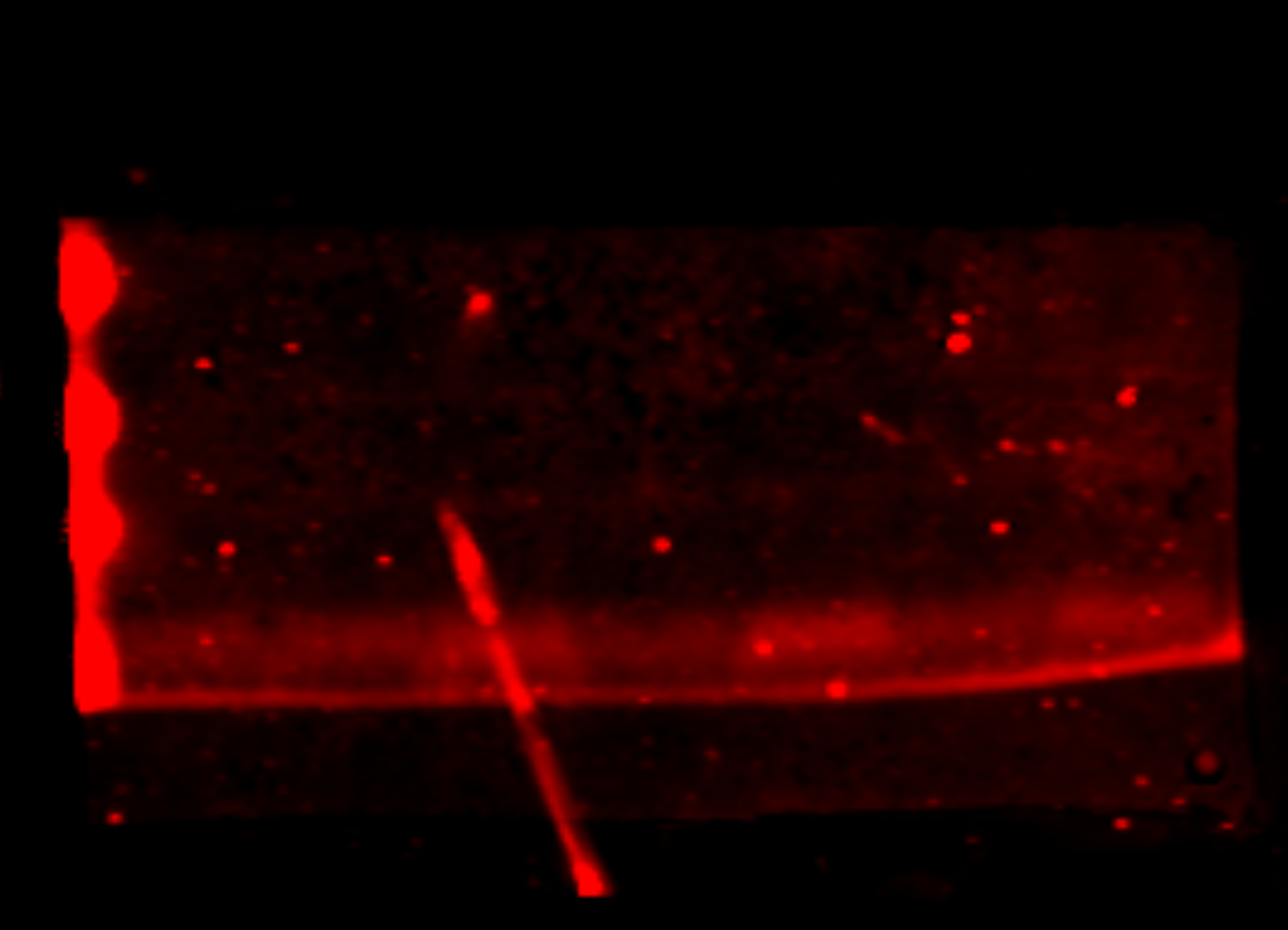

Supplement: Supplementary file 13 — Source Data [file 41467_2024_49721_MOESM13_ESM.zip › 406988_4_data_set_9156724_sddqhm/Source data-Supplementary Dataset/Fig4A and S14A/Anti-H3K363.tif]

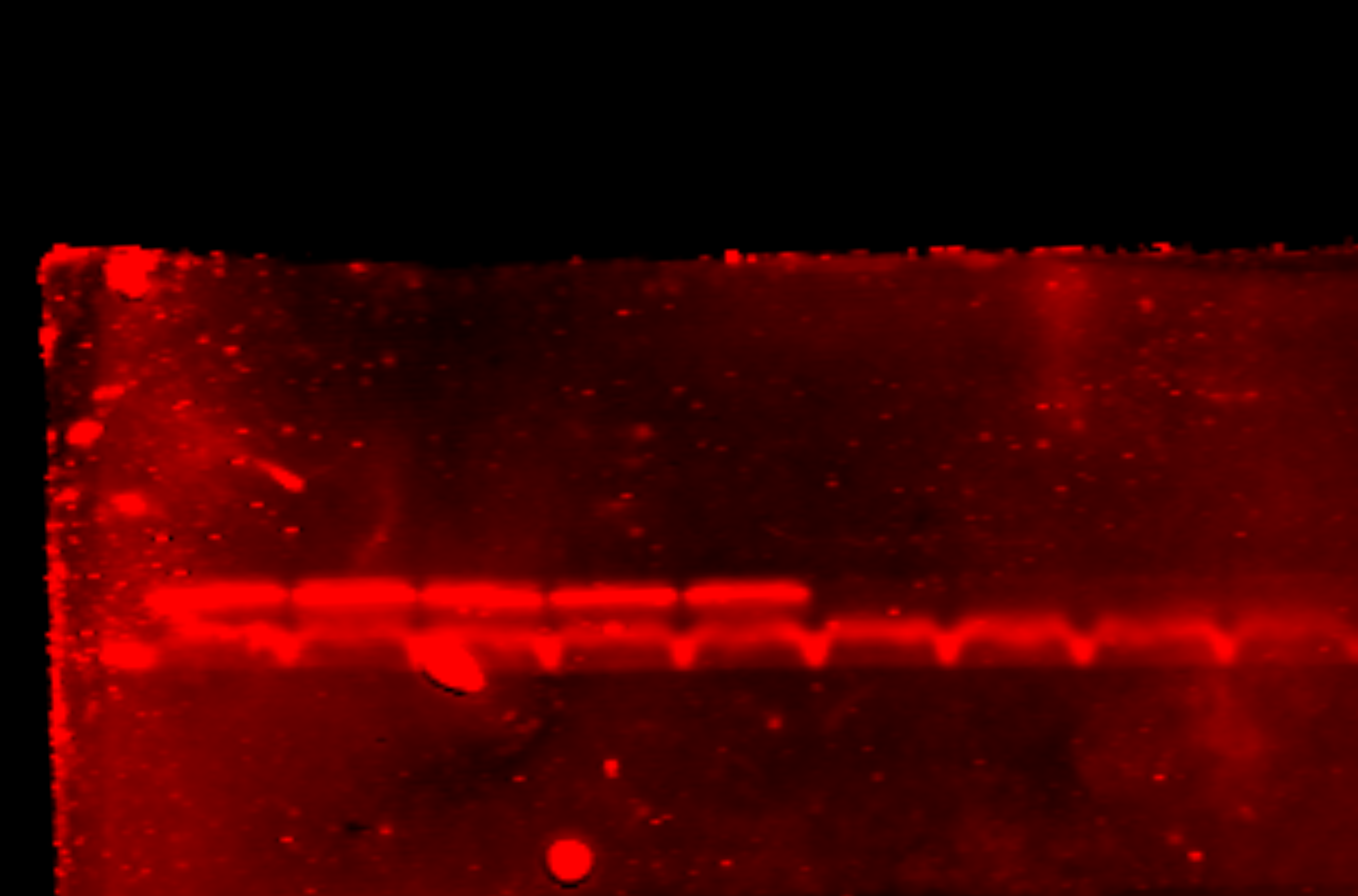

Supplement: Supplementary file 13 — Source Data [file 41467_2024_49721_MOESM13_ESM.zip › 406988_4_data_set_9156724_sddqhm/Source data-Supplementary Dataset/Fig4A and S14A/Anti-H3K4 loading.tif]

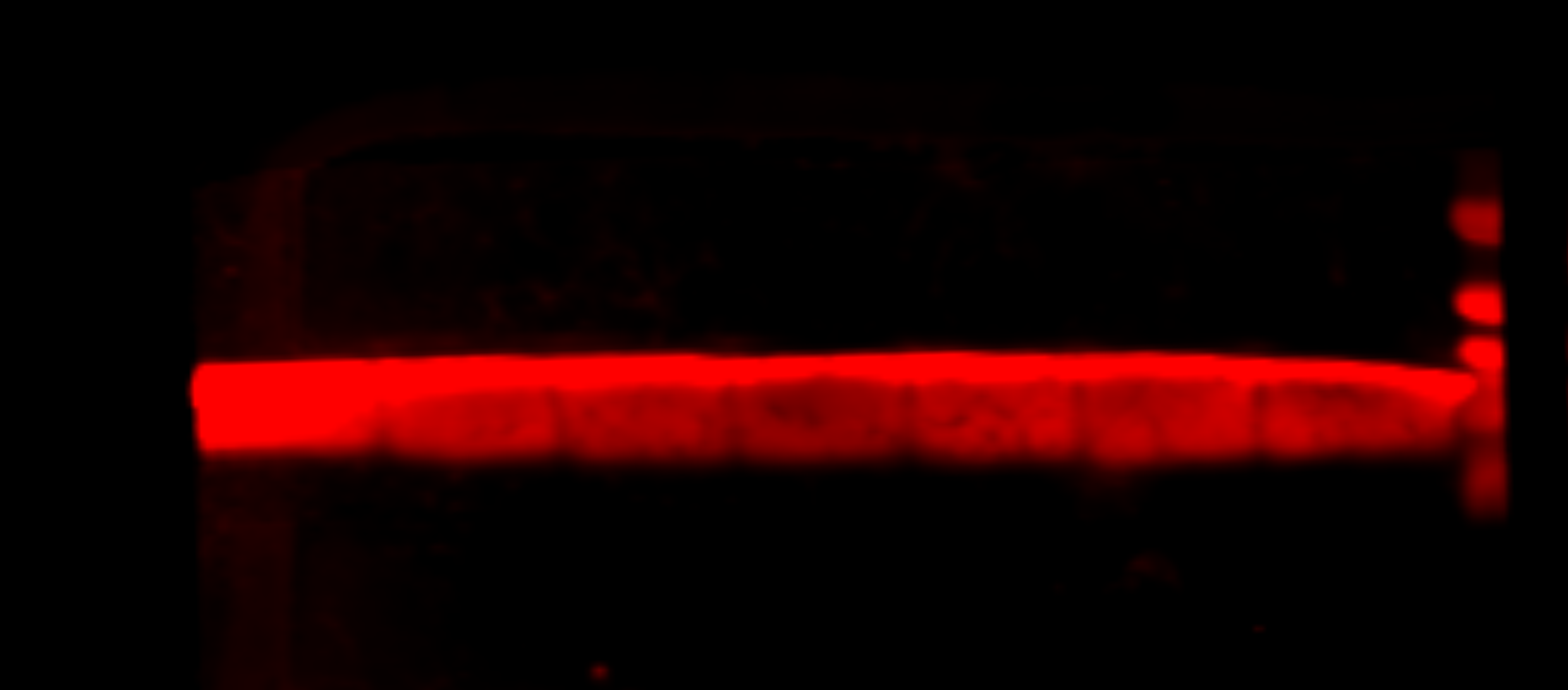

Supplement: Supplementary file 13 — Source Data [file 41467_2024_49721_MOESM13_ESM.zip › 406988_4_data_set_9156724_sddqhm/Source data-Supplementary Dataset/Fig4A and S14A/Anti-H3K4.tif]

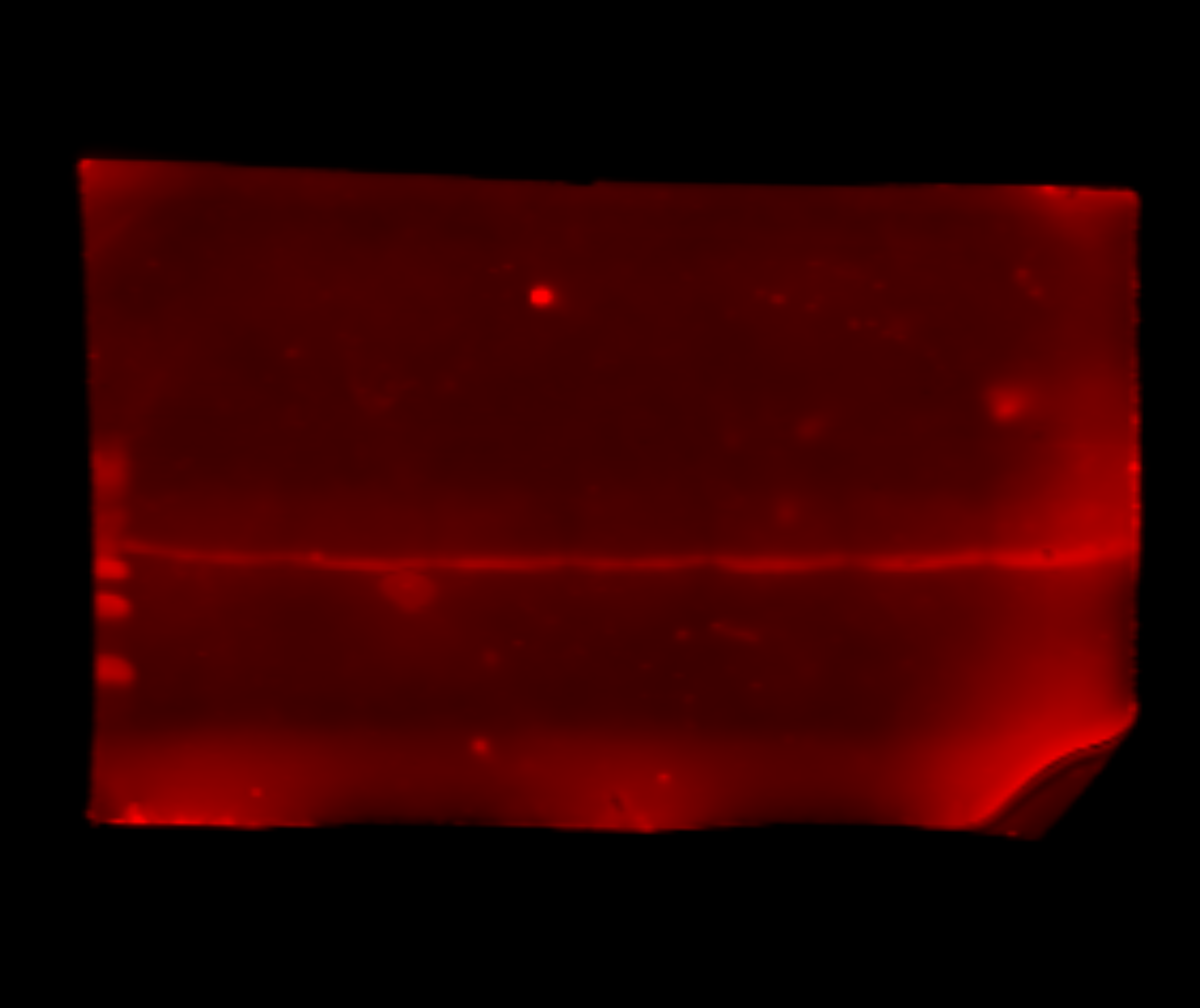

Supplement: Supplementary file 13 — Source Data [file 41467_2024_49721_MOESM13_ESM.zip › 406988_4_data_set_9156724_sddqhm/Source data-Supplementary Dataset/Fig4A and S14A/Anti-H3K9 loading.tif]

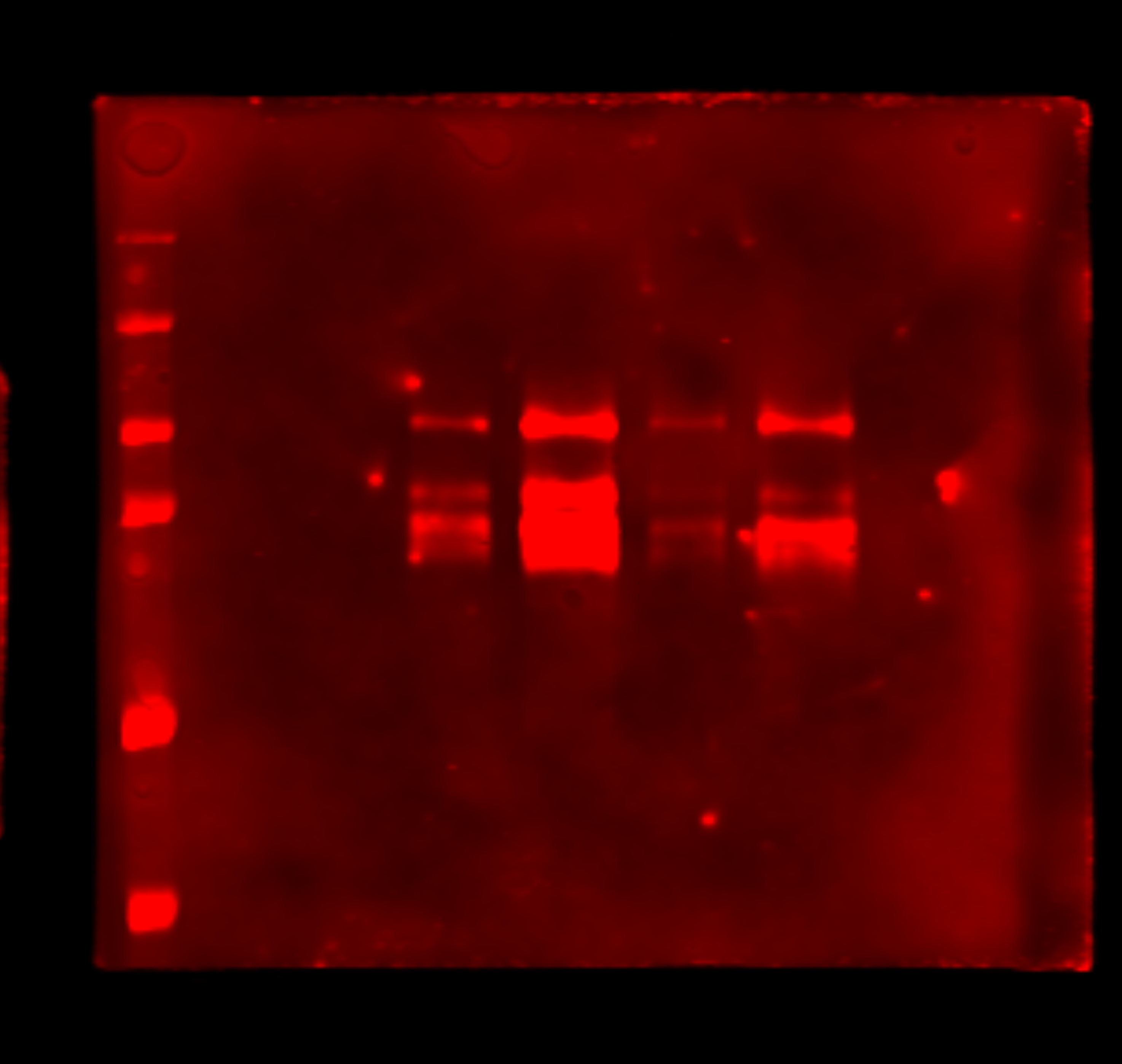

Supplement: Supplementary file 13 — Source Data [file 41467_2024_49721_MOESM13_ESM.zip › 406988_4_data_set_9156724_sddqhm/Source data-Supplementary Dataset/Fig4A and S14A/Anti-JMJ18.tif]

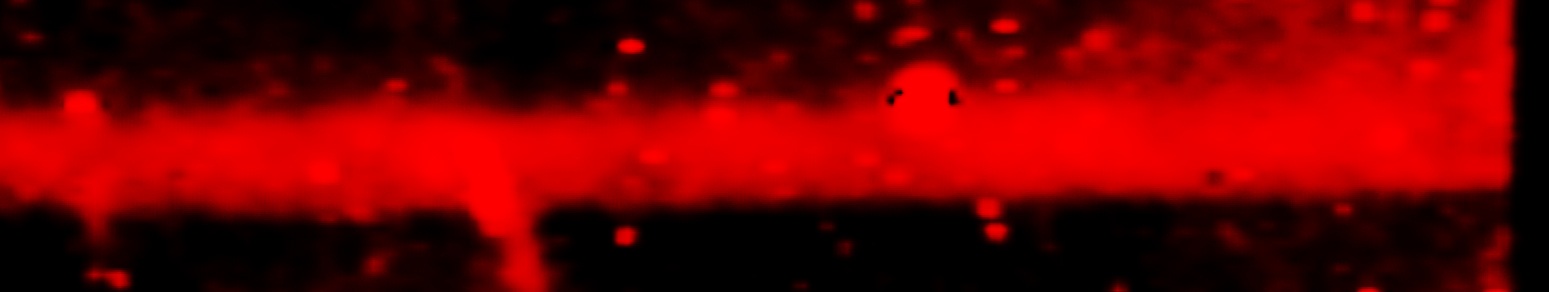

Supplement: Supplementary file 13 — Source Data [file 41467_2024_49721_MOESM13_ESM.zip › 406988_4_data_set_9156724_sddqhm/Source data-Supplementary Dataset/Fig4A and S14A/H3K27me3 loading.jpg]

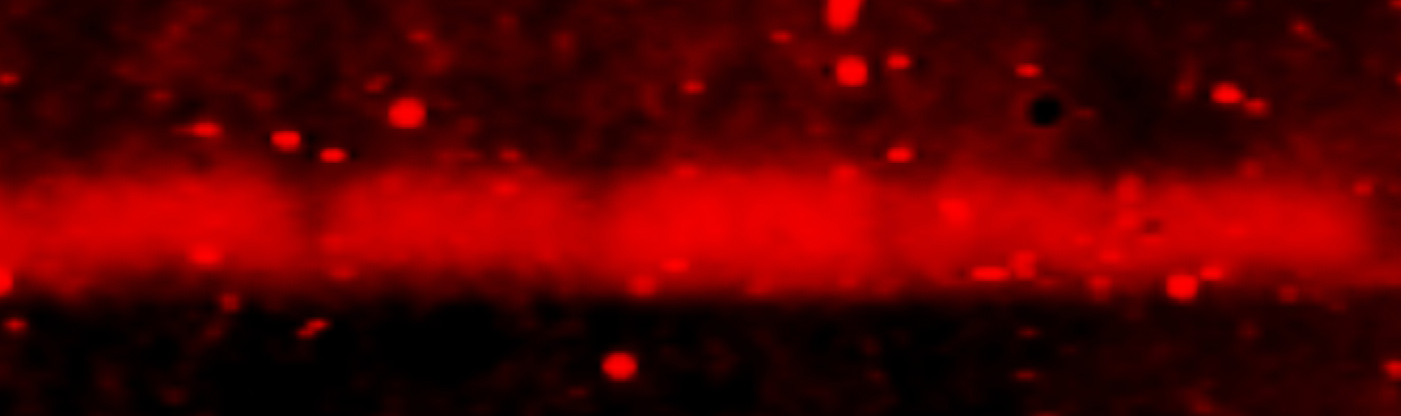

Supplement: Supplementary file 13 — Source Data [file 41467_2024_49721_MOESM13_ESM.zip › 406988_4_data_set_9156724_sddqhm/Source data-Supplementary Dataset/Fig4A and S14A/H3K27me3.jpg]

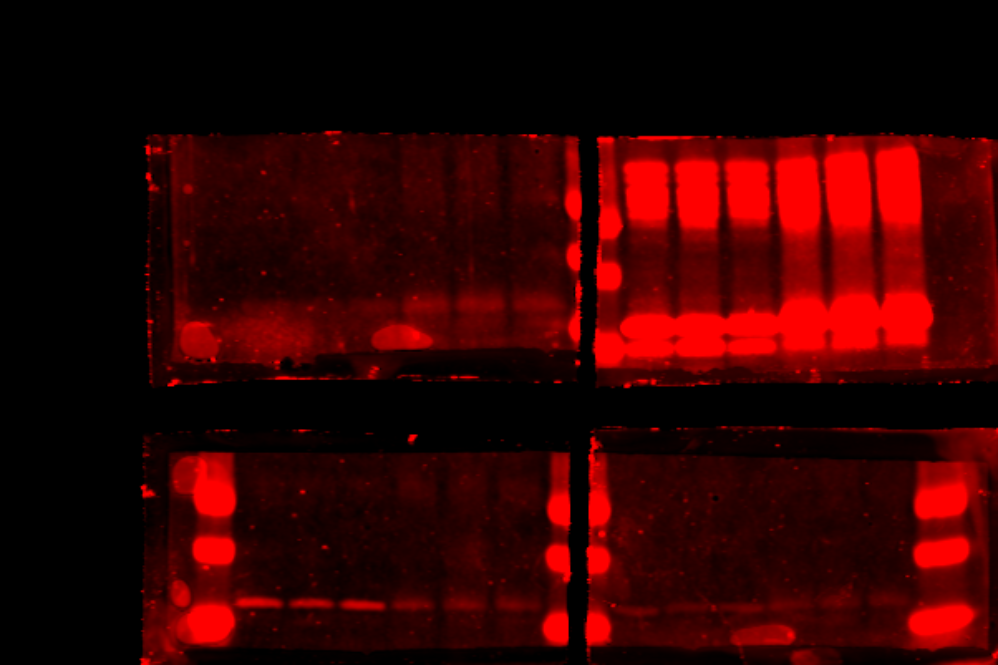

Supplement: Supplementary file 13 — Source Data [file 41467_2024_49721_MOESM13_ESM.zip › 406988_4_data_set_9156724_sddqhm/Source data-Supplementary Dataset/Fig4B and S14B/190425 GFP IP JMJ18 methylation assay 1.tif]

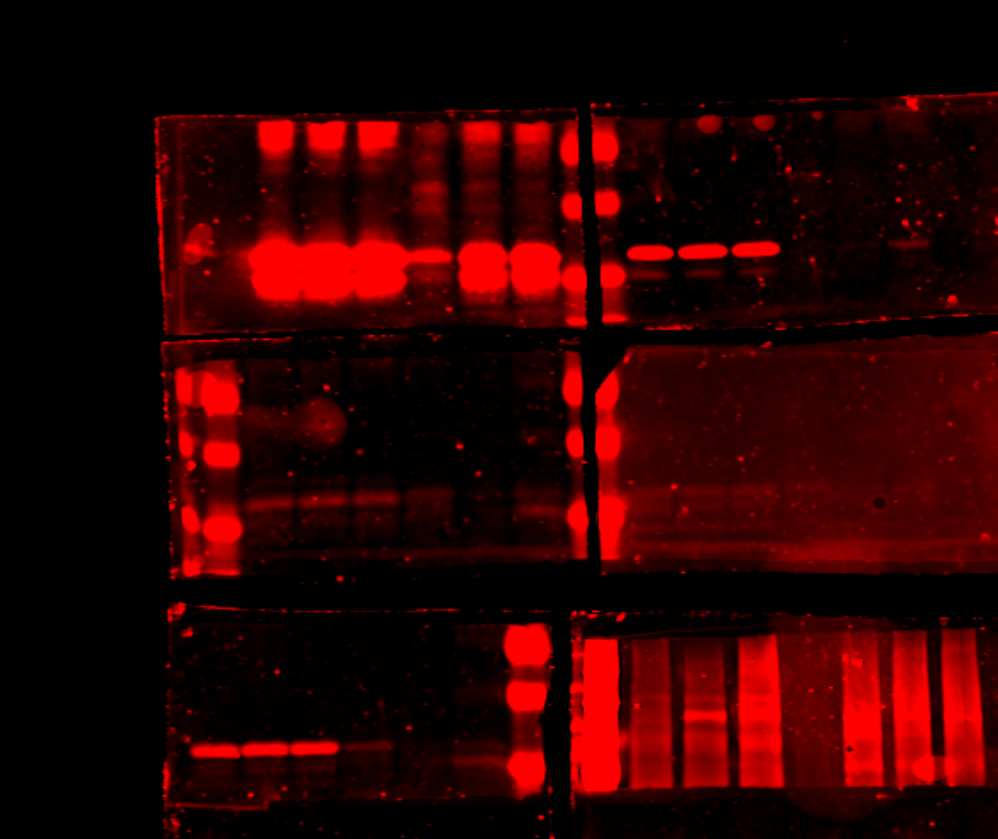

Supplement: Supplementary file 13 — Source Data [file 41467_2024_49721_MOESM13_ESM.zip › 406988_4_data_set_9156724_sddqhm/Source data-Supplementary Dataset/Fig4B and S14B/190518 JMJ18 GFP IP demethylation assay 2.tif]

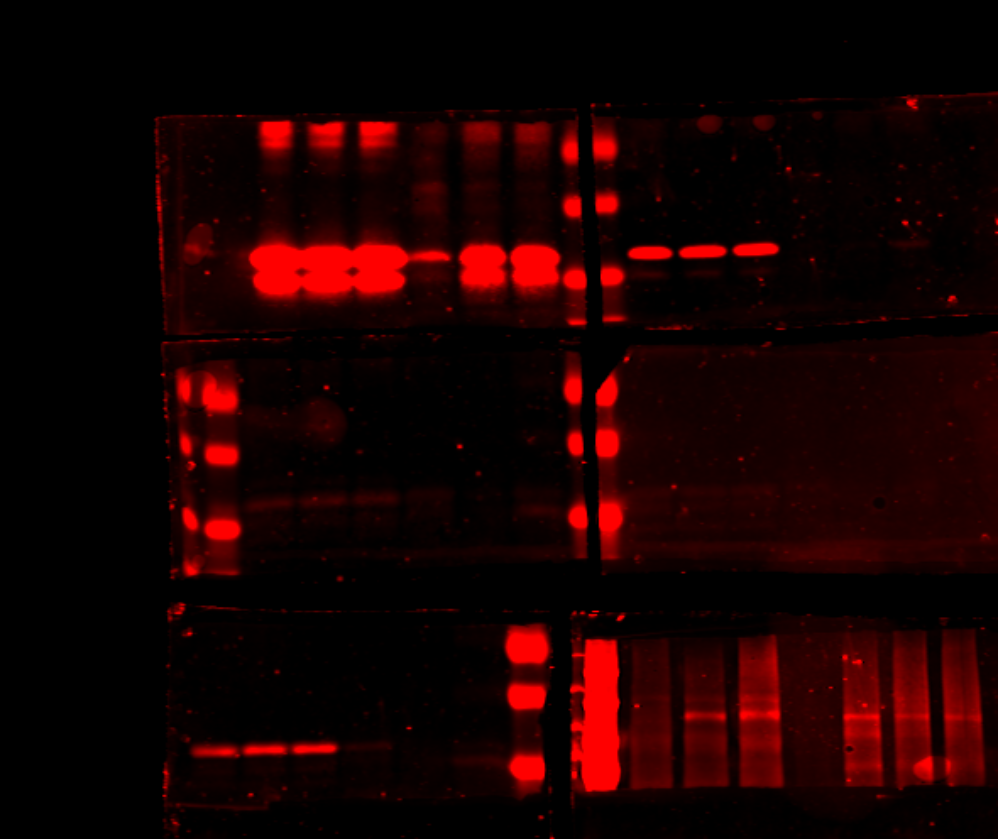

Supplement: Supplementary file 13 — Source Data [file 41467_2024_49721_MOESM13_ESM.zip › 406988_4_data_set_9156724_sddqhm/Source data-Supplementary Dataset/Fig4B and S14B/190518 JMJ18 GFP IP demethylation assay 3.tif]

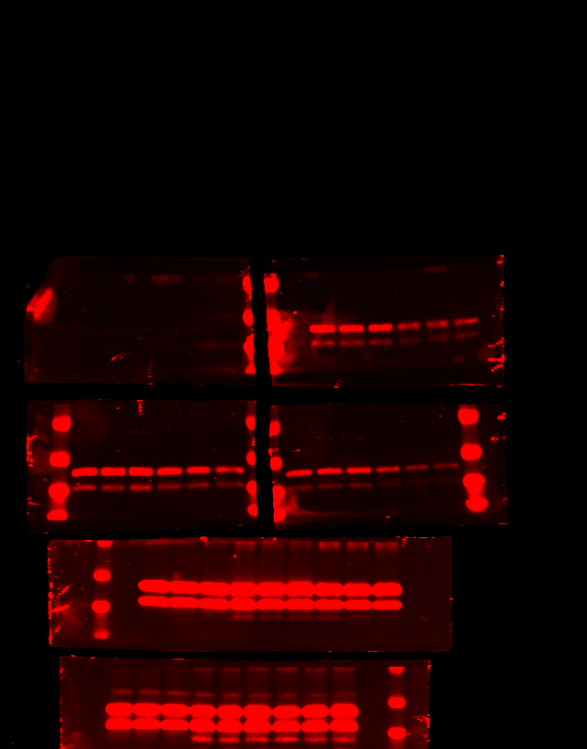

Supplement: Supplementary file 13 — Source Data [file 41467_2024_49721_MOESM13_ESM.zip › 406988_4_data_set_9156724_sddqhm/Source data-Supplementary Dataset/Fig4B and S14B/20190831 BrJMJ18 demethylase assay 6.tif]

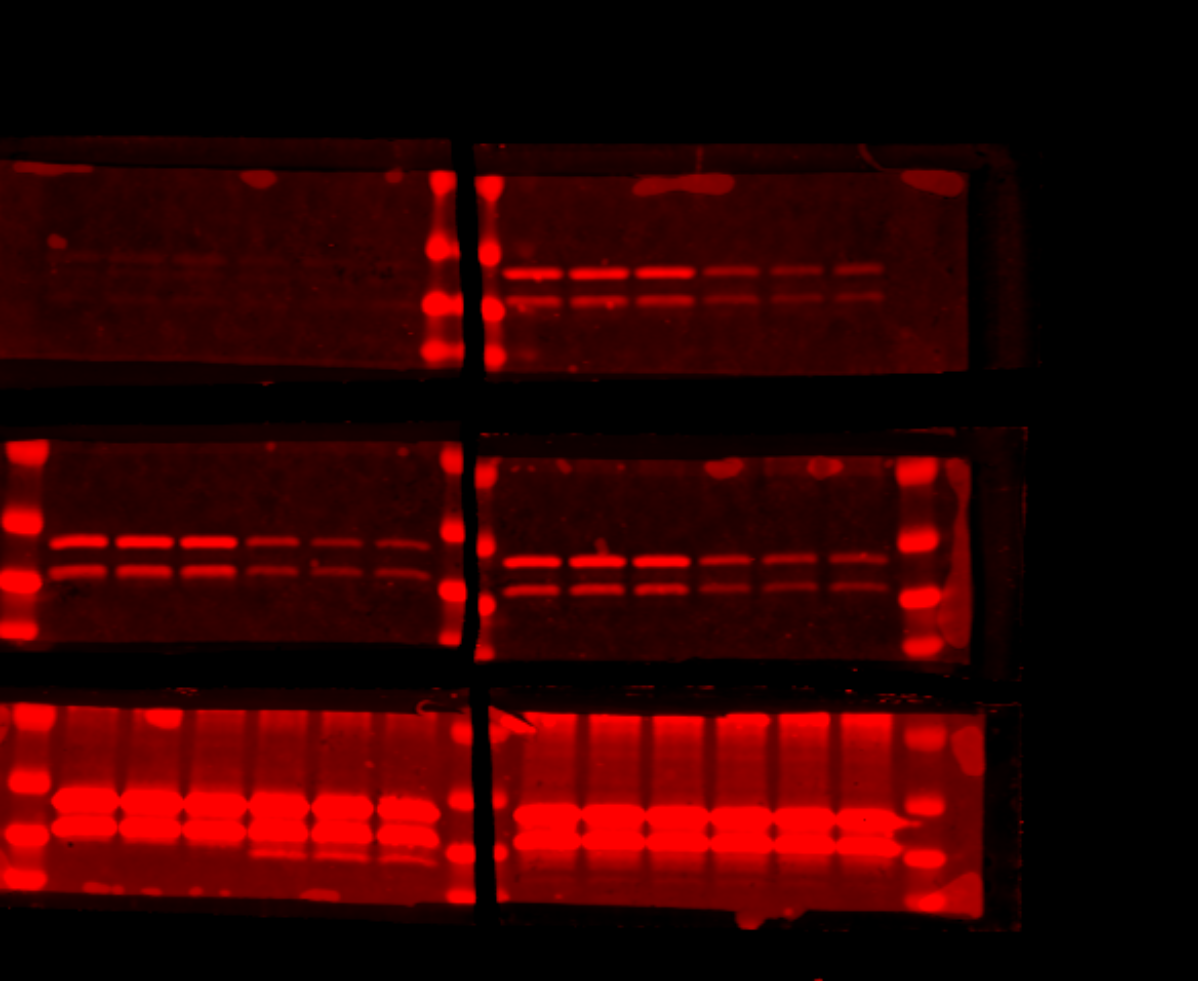

Supplement: Supplementary file 13 — Source Data [file 41467_2024_49721_MOESM13_ESM.zip › 406988_4_data_set_9156724_sddqhm/Source data-Supplementary Dataset/Fig4B and S14B/20191011 18GFP demethylase assay 1.tif]

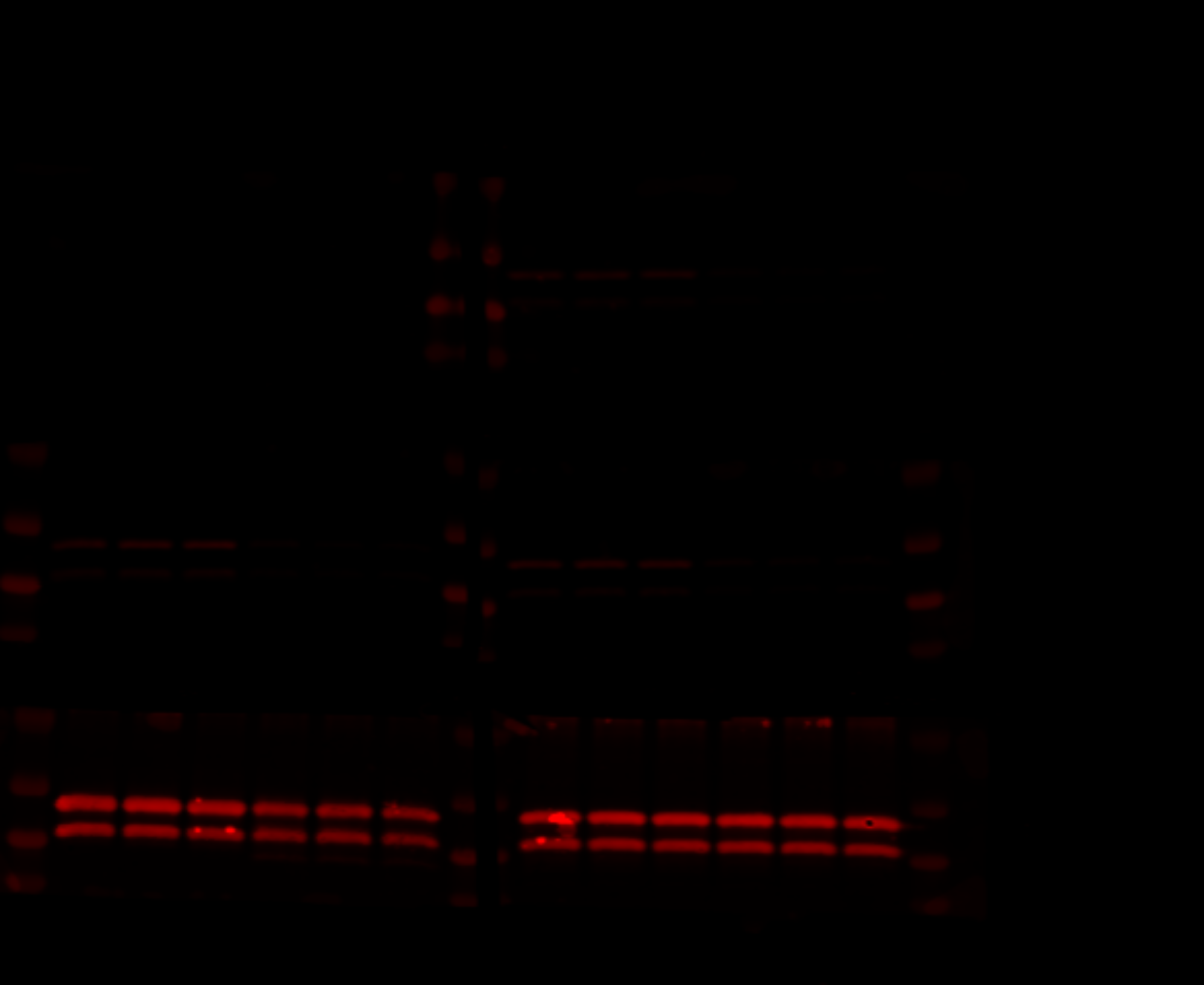

Supplement: Supplementary file 13 — Source Data [file 41467_2024_49721_MOESM13_ESM.zip › 406988_4_data_set_9156724_sddqhm/Source data-Supplementary Dataset/Fig4B and S14B/20191011 18GFP demethylase assay 2.tif]

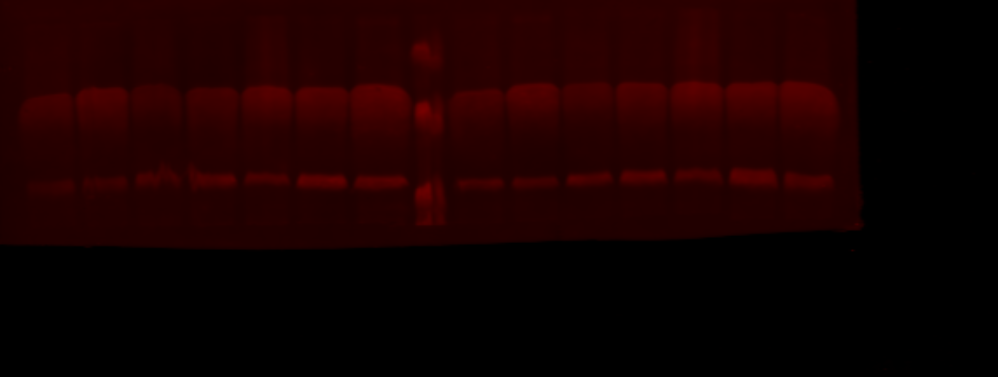

Supplement: Supplementary file 13 — Source Data [file 41467_2024_49721_MOESM13_ESM.zip › 406988_4_data_set_9156724_sddqhm/Source data-Supplementary Dataset/Fig4B and S14B/H3 4 9 27 36 me3 loading.tif]

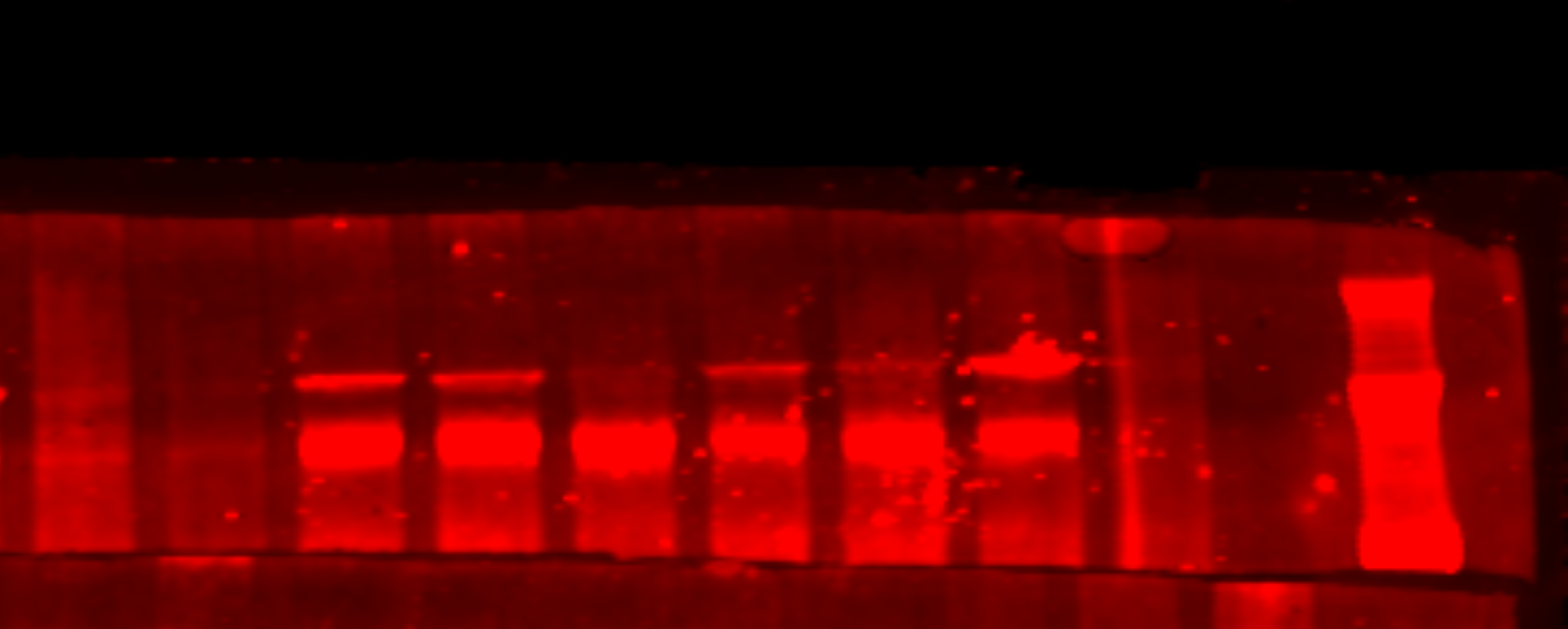

Supplement: Supplementary file 13 — Source Data [file 41467_2024_49721_MOESM13_ESM.zip › 406988_4_data_set_9156724_sddqhm/Source data-Supplementary Dataset/Fig4C/BrJMJ18.tif]

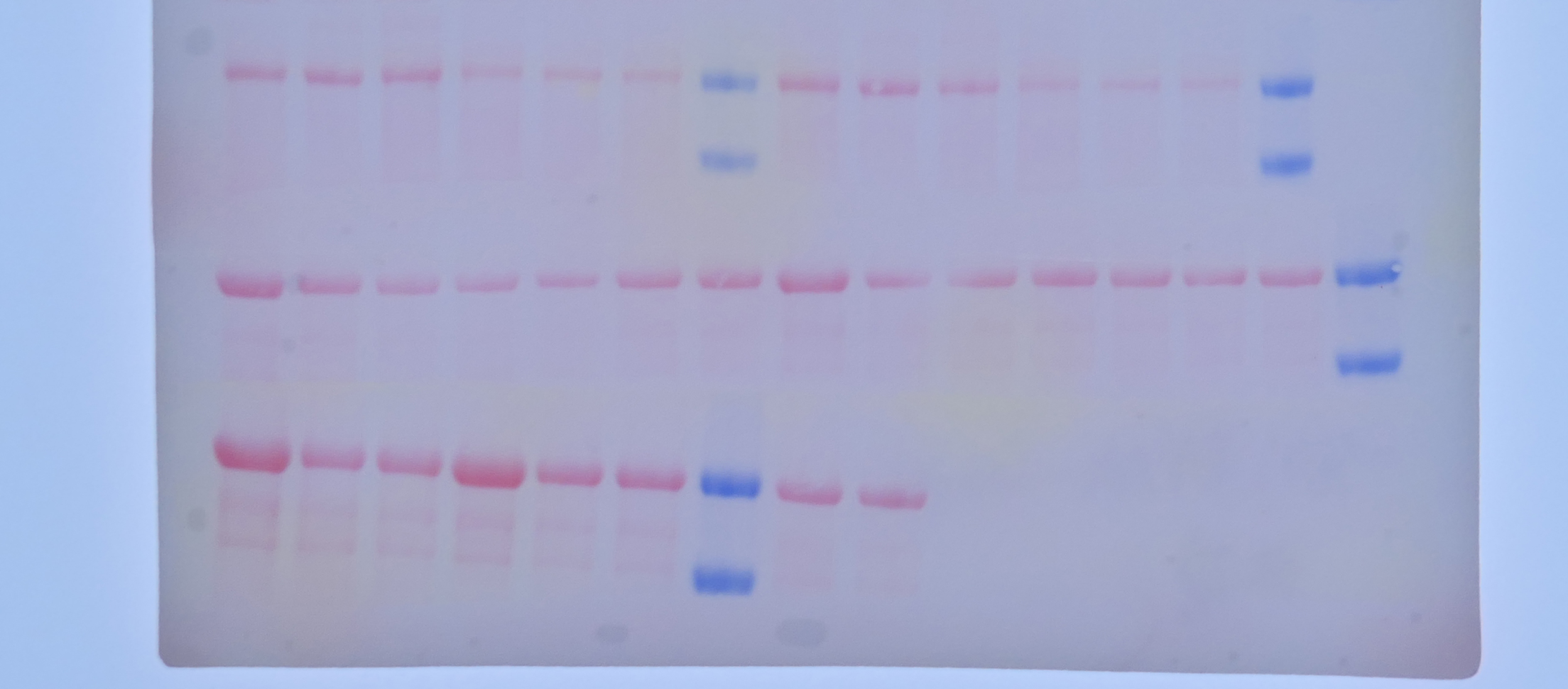

Supplement: Supplementary file 13 — Source Data [file 41467_2024_49721_MOESM13_ESM.zip › 406988_4_data_set_9156724_sddqhm/Source data-Supplementary Dataset/Fig4C/Fig4C GFP loading.jpg]

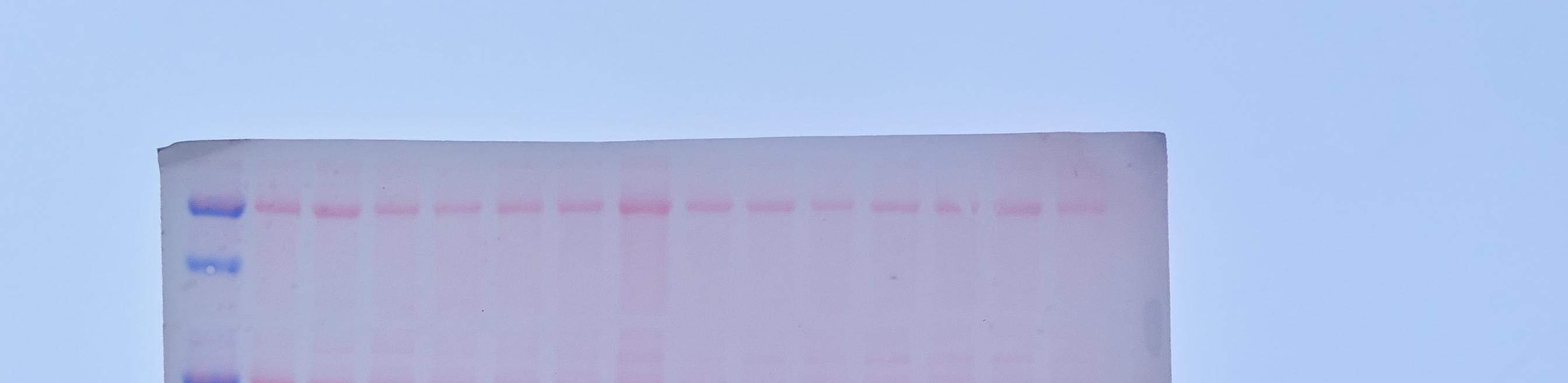

Supplement: Supplementary file 13 — Source Data [file 41467_2024_49721_MOESM13_ESM.zip › 406988_4_data_set_9156724_sddqhm/Source data-Supplementary Dataset/Fig4C/Fig4C histone loading.jpg]

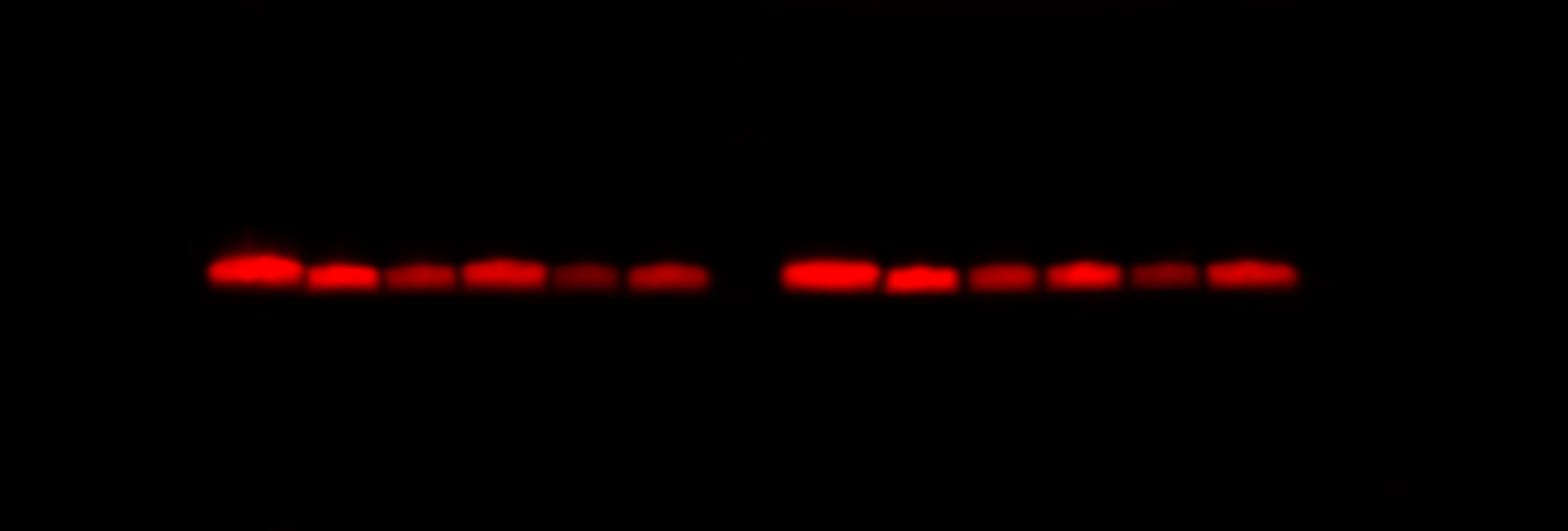

Supplement: Supplementary file 13 — Source Data [file 41467_2024_49721_MOESM13_ESM.zip › 406988_4_data_set_9156724_sddqhm/Source data-Supplementary Dataset/Fig4C/H3K36 me2 me3.tif]

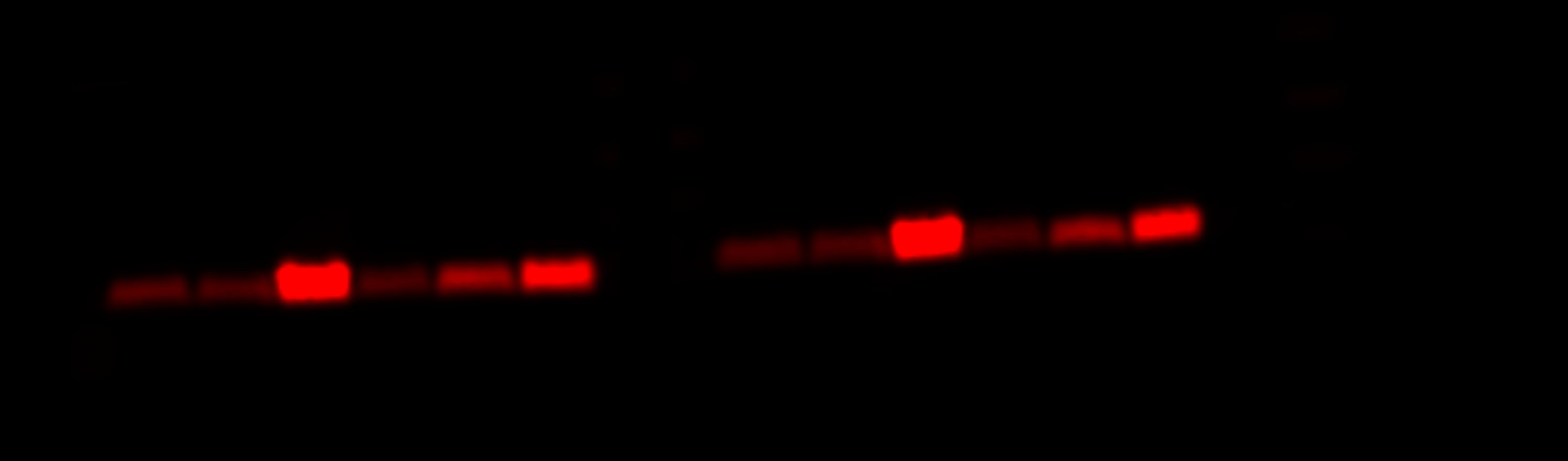

Supplement: Supplementary file 13 — Source Data [file 41467_2024_49721_MOESM13_ESM.zip › 406988_4_data_set_9156724_sddqhm/Source data-Supplementary Dataset/Fig4C-1.tif]

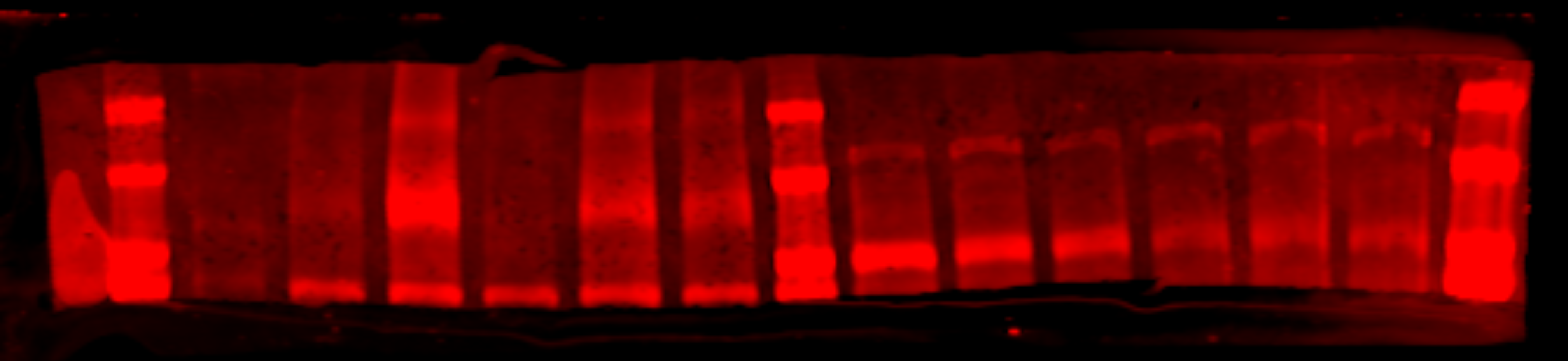

Supplement: Supplementary file 13 — Source Data [file 41467_2024_49721_MOESM13_ESM.zip › 406988_4_data_set_9156724_sddqhm/Source data-Supplementary Dataset/Fig4C-2.tif]

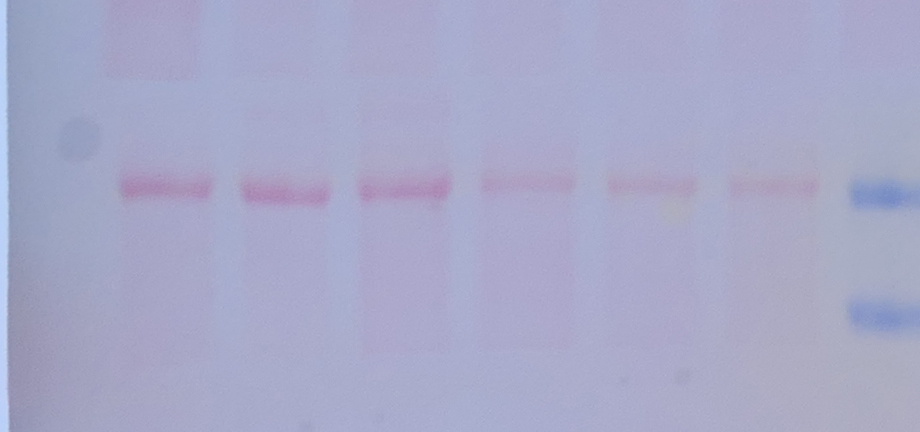

Supplement: Supplementary file 13 — Source Data [file 41467_2024_49721_MOESM13_ESM.zip › 406988_4_data_set_9156724_sddqhm/Source data-Supplementary Dataset/Fig4C-3.JPG]

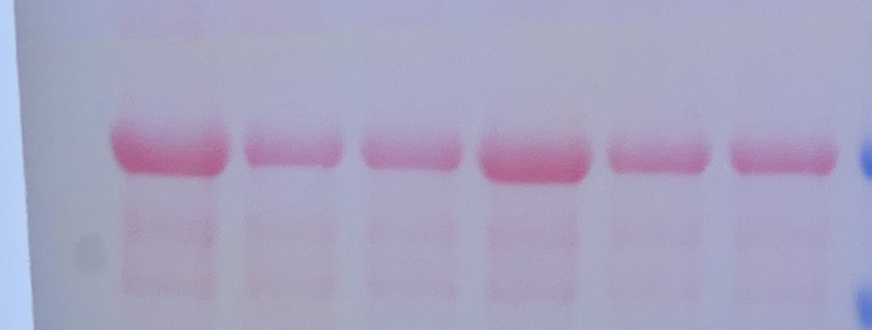

Supplement: Supplementary file 13 — Source Data [file 41467_2024_49721_MOESM13_ESM.zip › 406988_4_data_set_9156724_sddqhm/Source data-Supplementary Dataset/Fig4C-4.jpg]

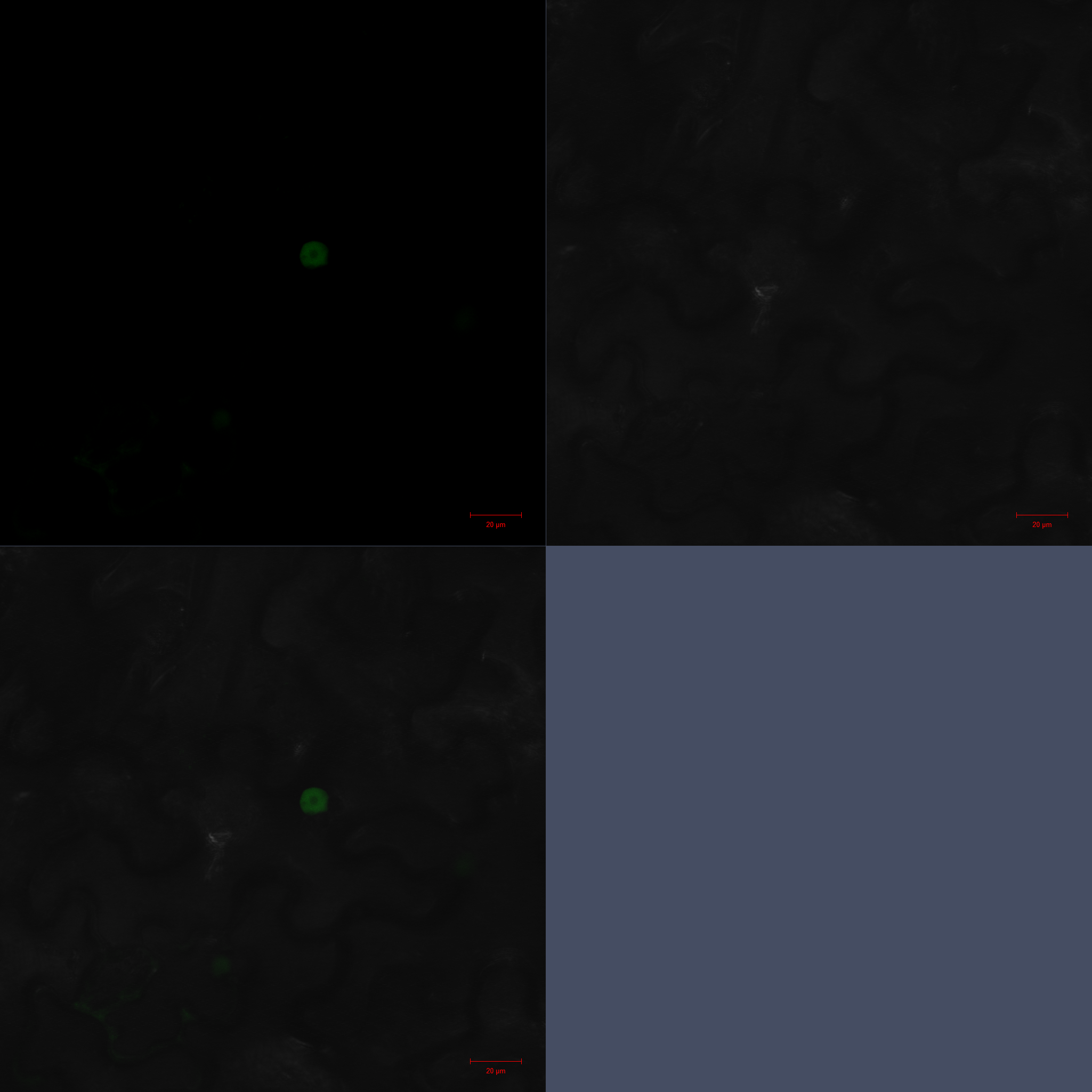

Supplement: Supplementary file 13 — Source Data [file 41467_2024_49721_MOESM13_ESM.zip › 406988_4_data_set_9156724_sddqhm/Source data-Supplementary Dataset/Fig4E-1.tif]

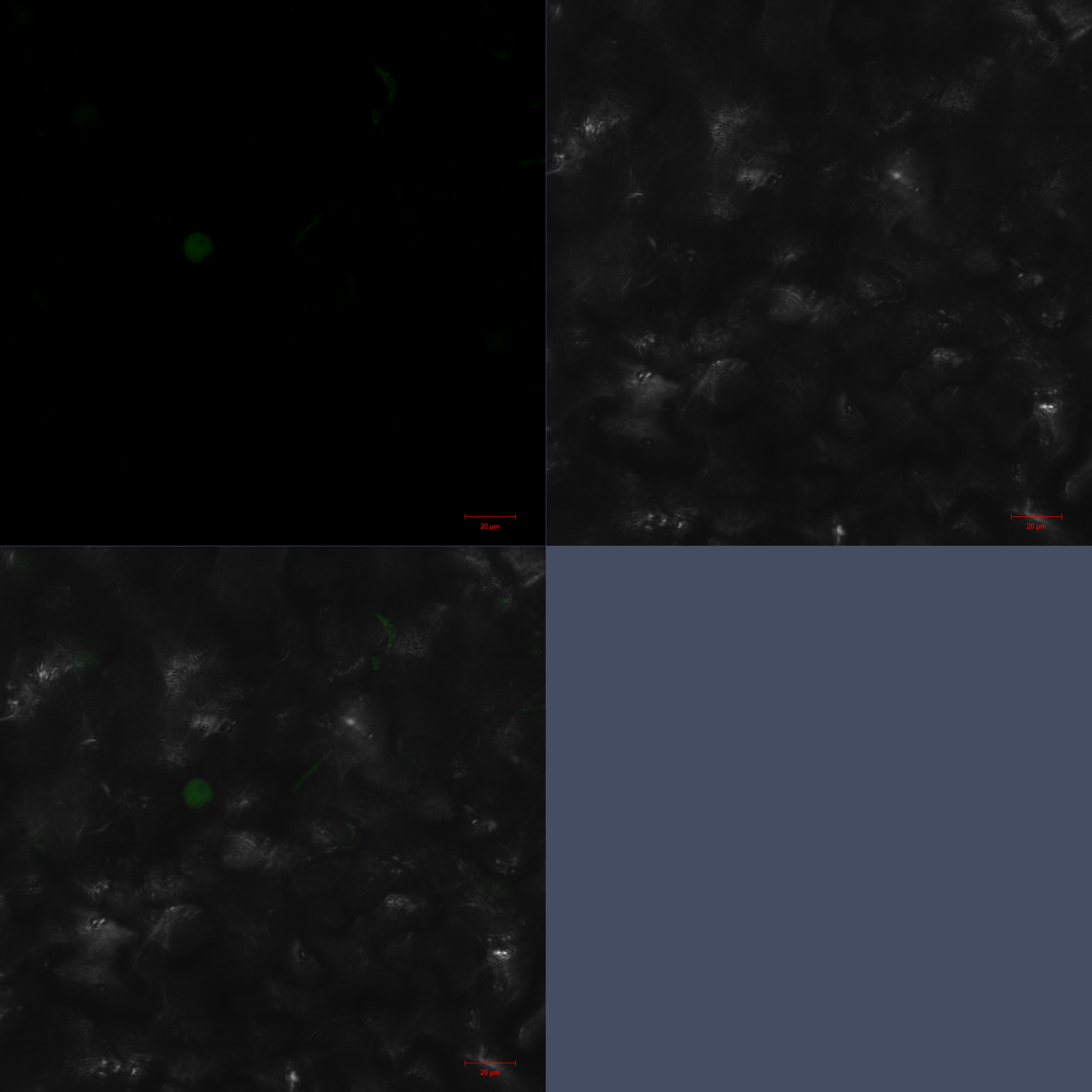

Supplement: Supplementary file 13 — Source Data [file 41467_2024_49721_MOESM13_ESM.zip › 406988_4_data_set_9156724_sddqhm/Source data-Supplementary Dataset/Fig4E-2.tif]

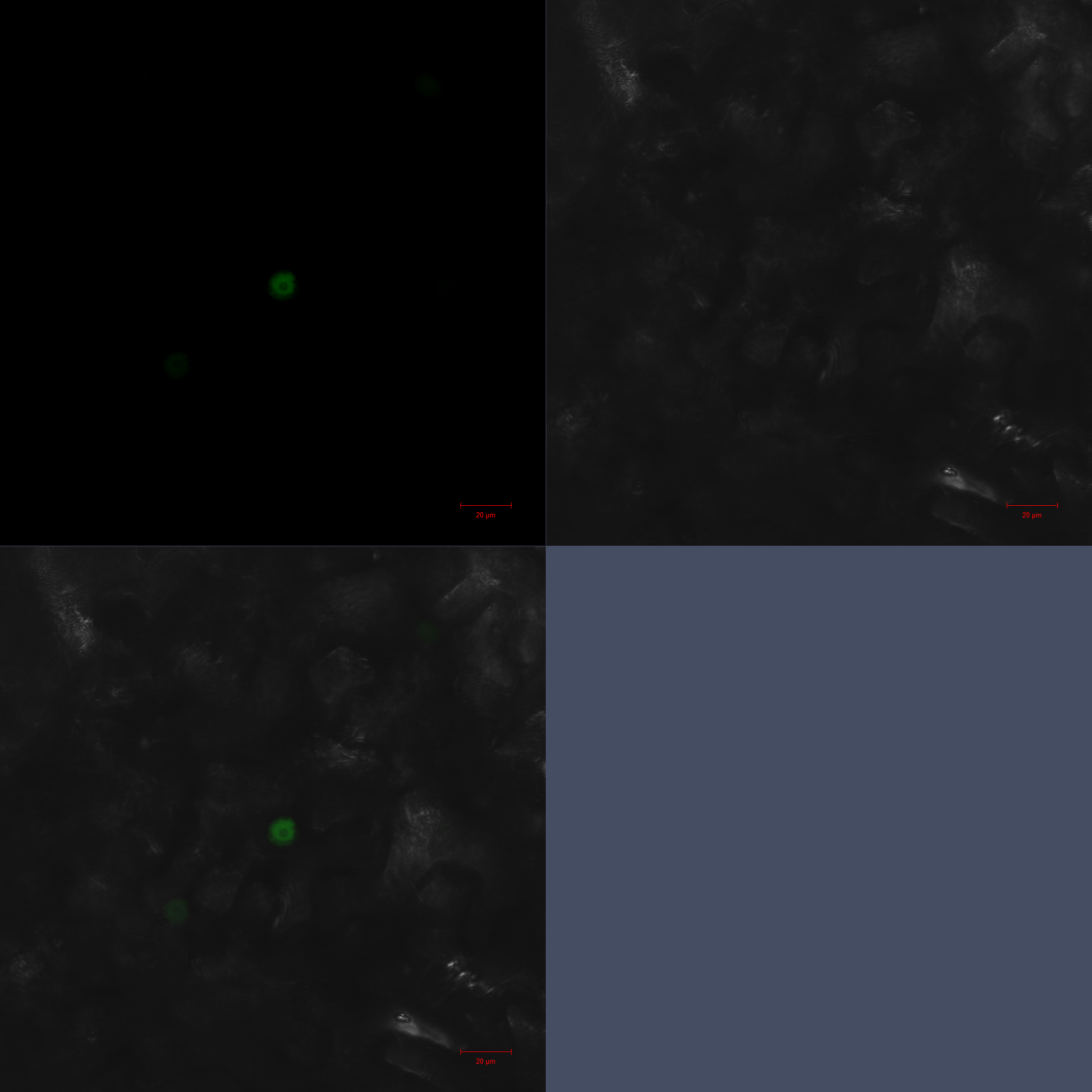

Supplement: Supplementary file 13 — Source Data [file 41467_2024_49721_MOESM13_ESM.zip › 406988_4_data_set_9156724_sddqhm/Source data-Supplementary Dataset/Fig4F-1.tif]

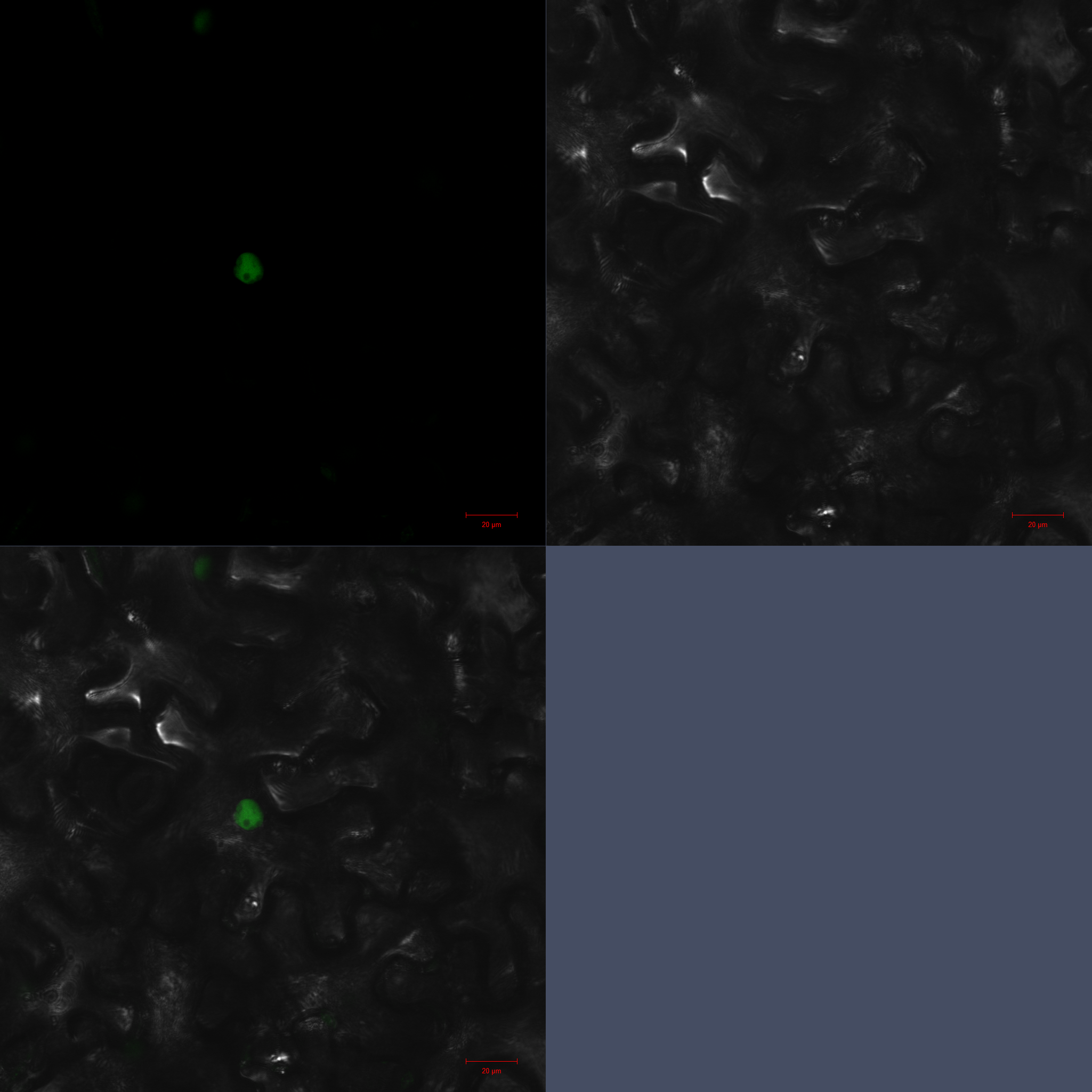

Supplement: Supplementary file 13 — Source Data [file 41467_2024_49721_MOESM13_ESM.zip › 406988_4_data_set_9156724_sddqhm/Source data-Supplementary Dataset/Fig4F-2.tif]

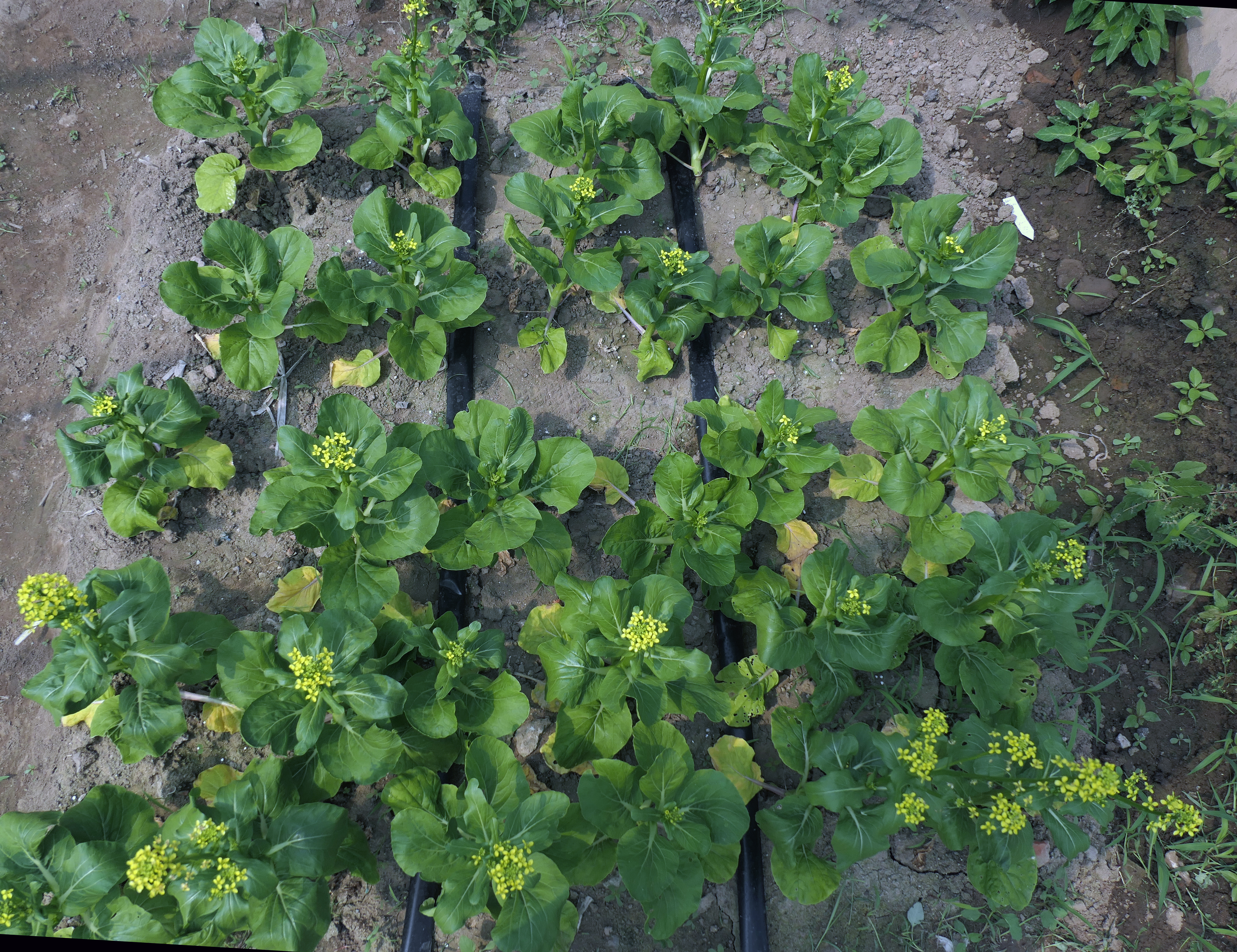

Supplement: Supplementary file 13 — Source Data [file 41467_2024_49721_MOESM13_ESM.zip › 406988_4_data_set_9156724_sddqhm/Source data-Supplementary Dataset/Fig6D.jpg]

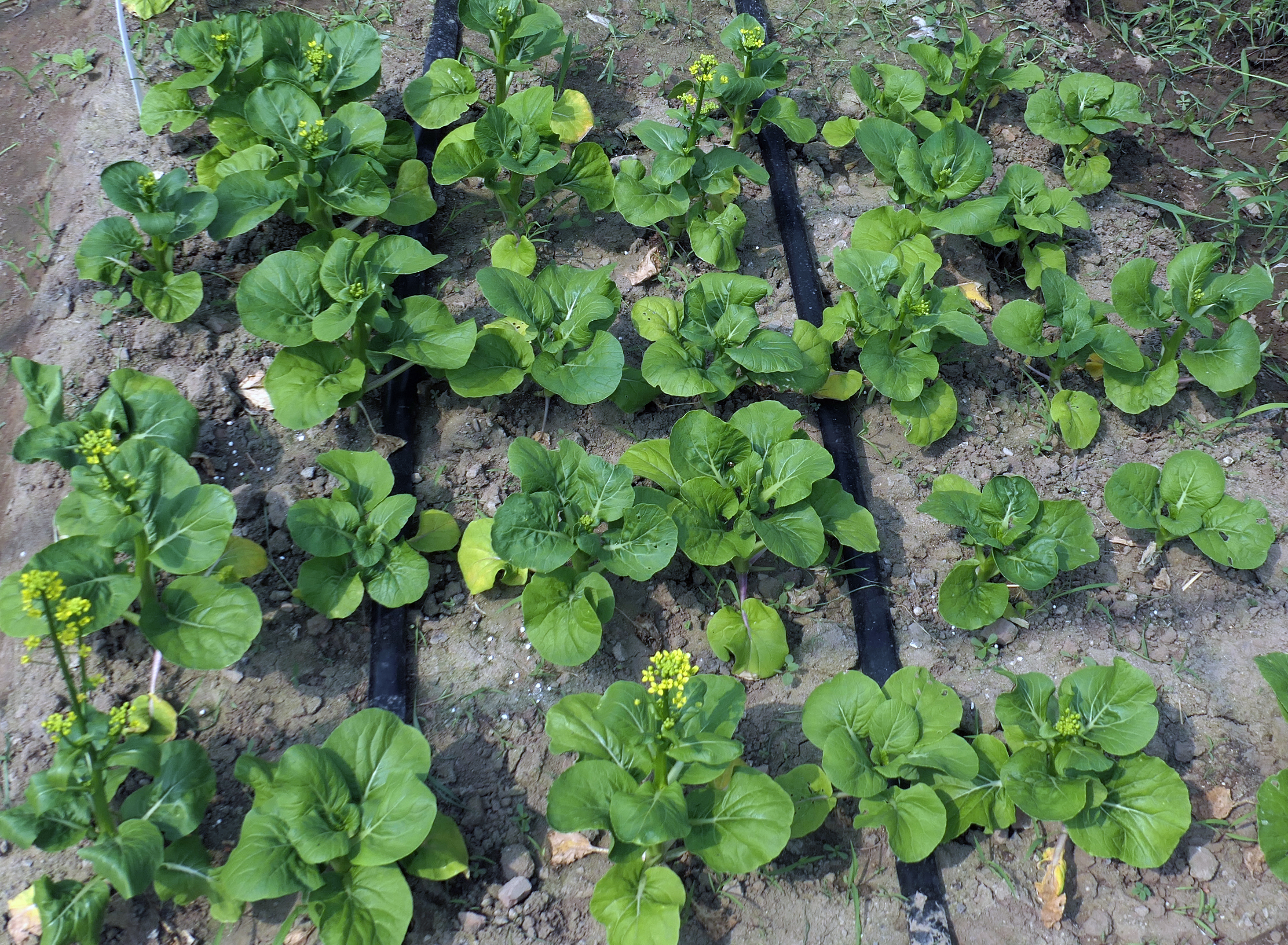

Supplement: Supplementary file 13 — Source Data [file 41467_2024_49721_MOESM13_ESM.zip › 406988_4_data_set_9156724_sddqhm/Source data-Supplementary Dataset/Fig6E.jpg]

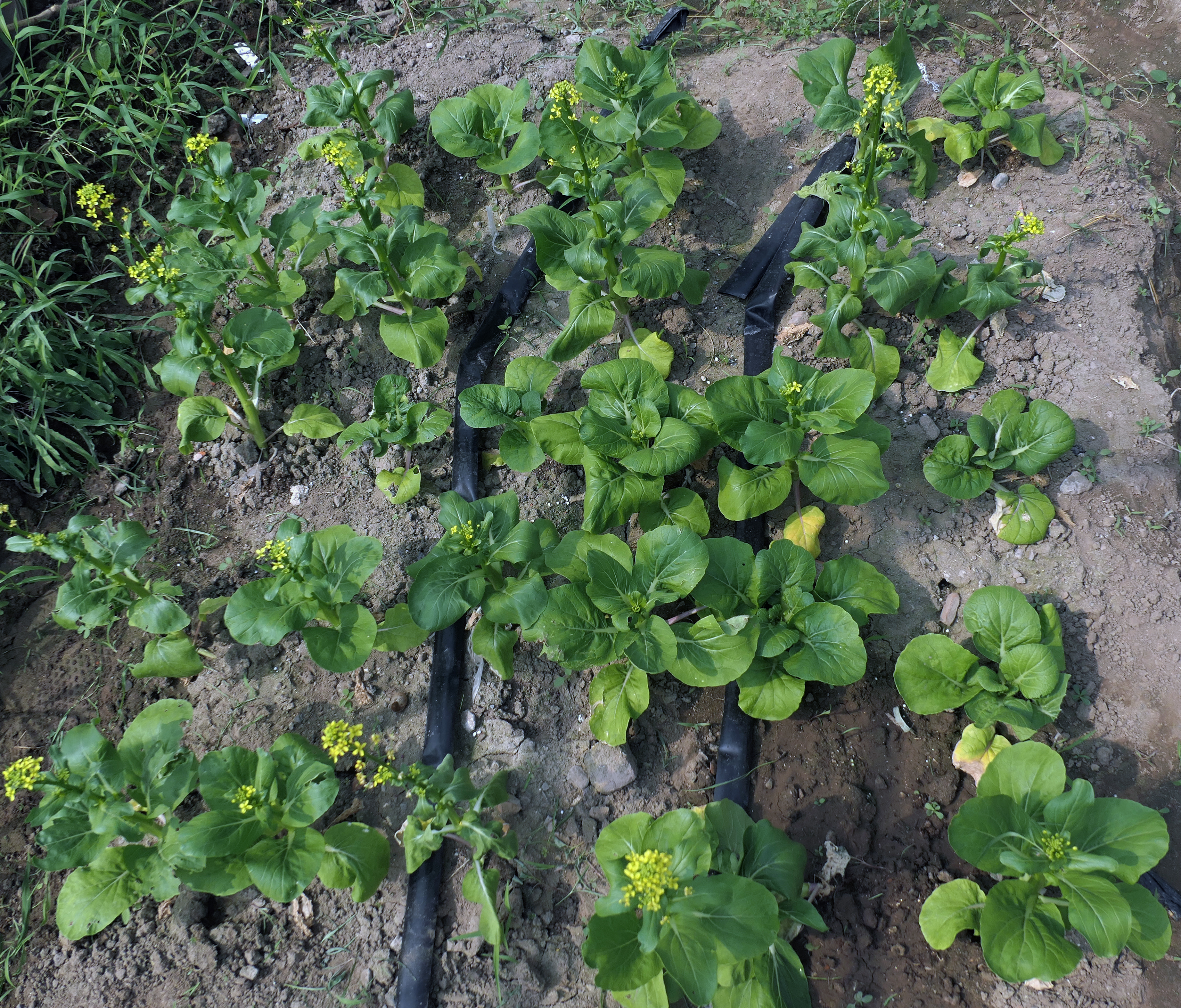

Supplement: Supplementary file 13 — Source Data [file 41467_2024_49721_MOESM13_ESM.zip › 406988_4_data_set_9156724_sddqhm/Source data-Supplementary Dataset/Fig6F.jpg]

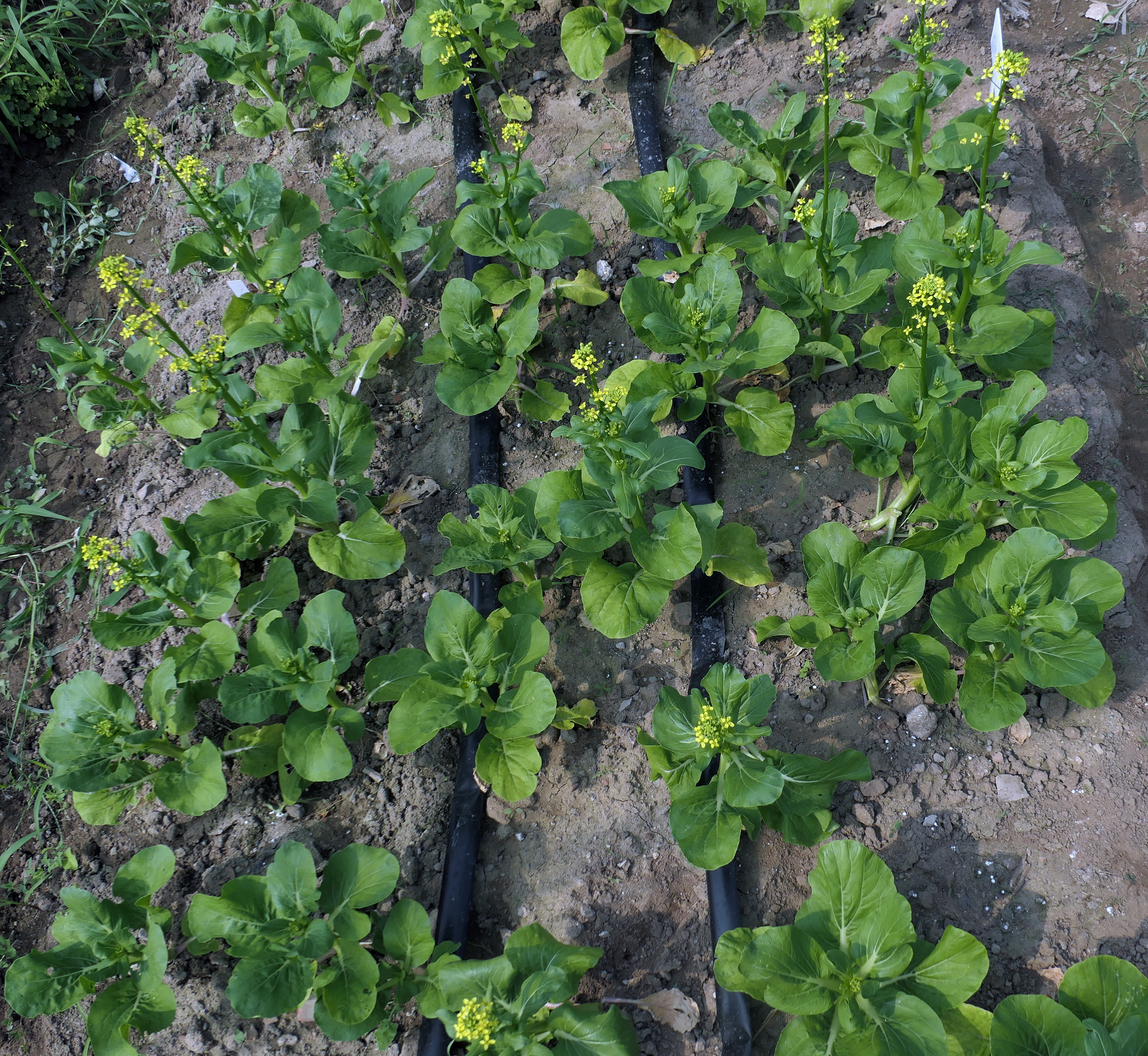

Supplement: Supplementary file 13 — Source Data [file 41467_2024_49721_MOESM13_ESM.zip › 406988_4_data_set_9156724_sddqhm/Source data-Supplementary Dataset/Fig6G.jpg]

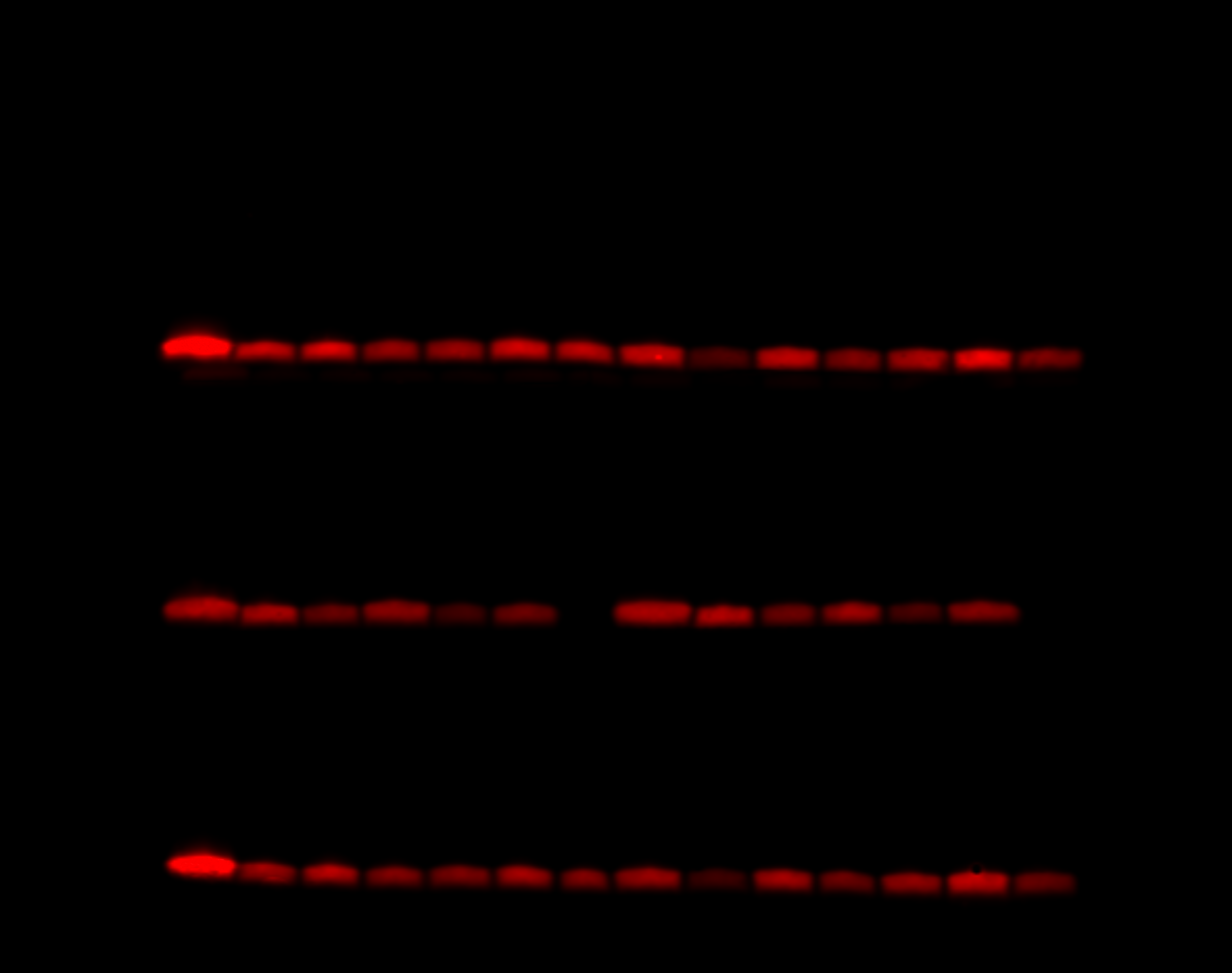

Supplement: Supplementary file 13 — Source Data [file 41467_2024_49721_MOESM13_ESM.zip › 406988_4_data_set_9156724_sddqhm/Source data-Supplementary Dataset/Fig8-1.tif]

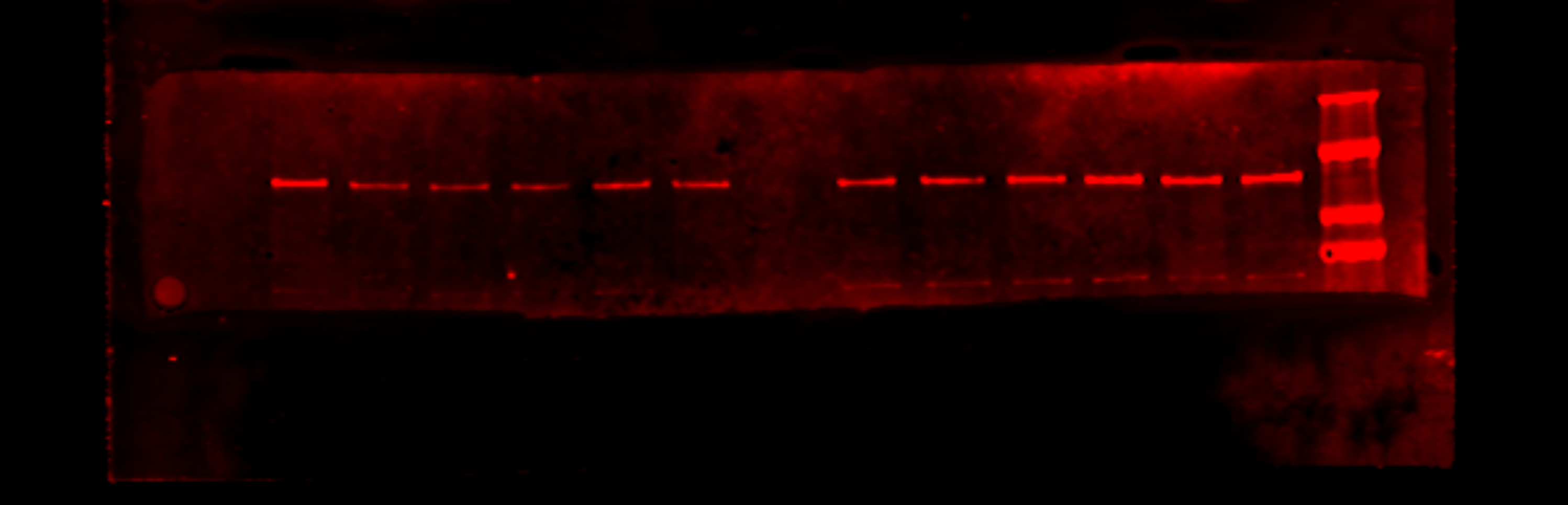

Supplement: Supplementary file 13 — Source Data [file 41467_2024_49721_MOESM13_ESM.zip › 406988_4_data_set_9156724_sddqhm/Source data-Supplementary Dataset/Fig8-2.tif]

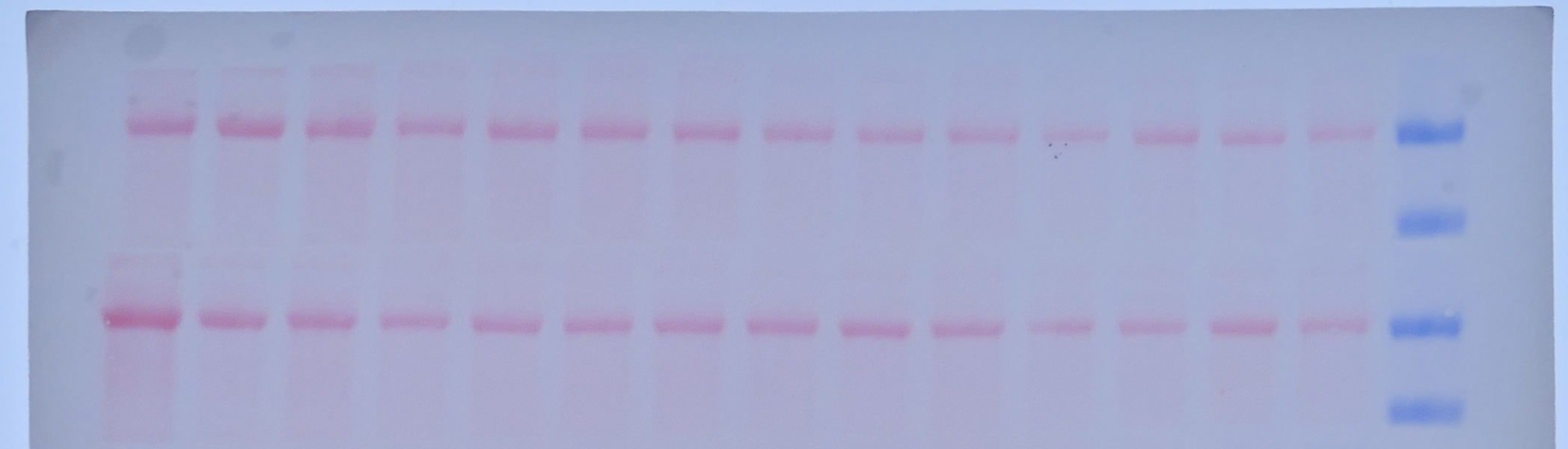

Supplement: Supplementary file 13 — Source Data [file 41467_2024_49721_MOESM13_ESM.zip › 406988_4_data_set_9156724_sddqhm/Source data-Supplementary Dataset/Fig8-3.jpg]

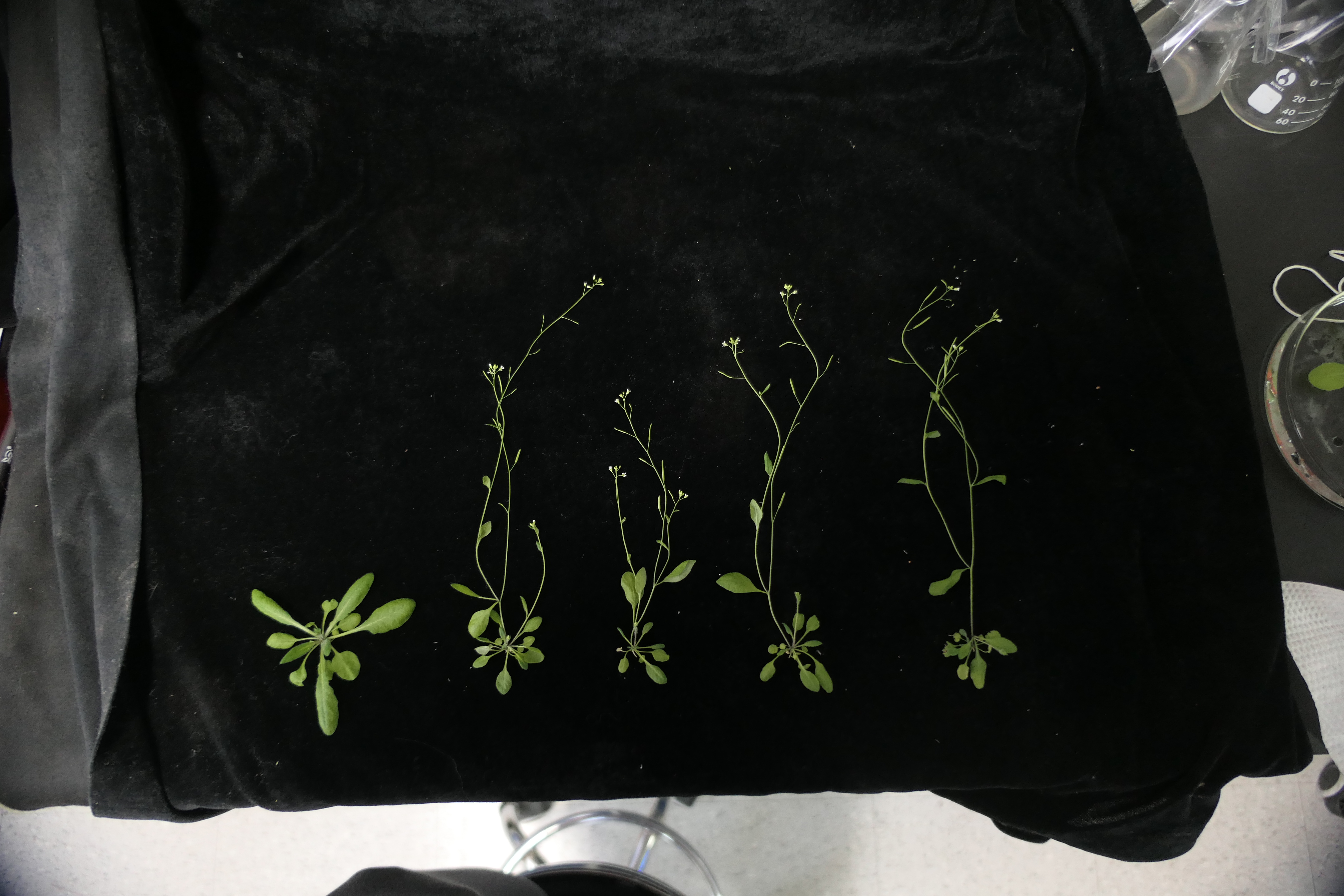

Supplement: Supplementary file 13 — Source Data [file 41467_2024_49721_MOESM13_ESM.zip › 406988_4_data_set_9156724_sddqhm/Source data-Supplementary Dataset/FigS12A.JPG]

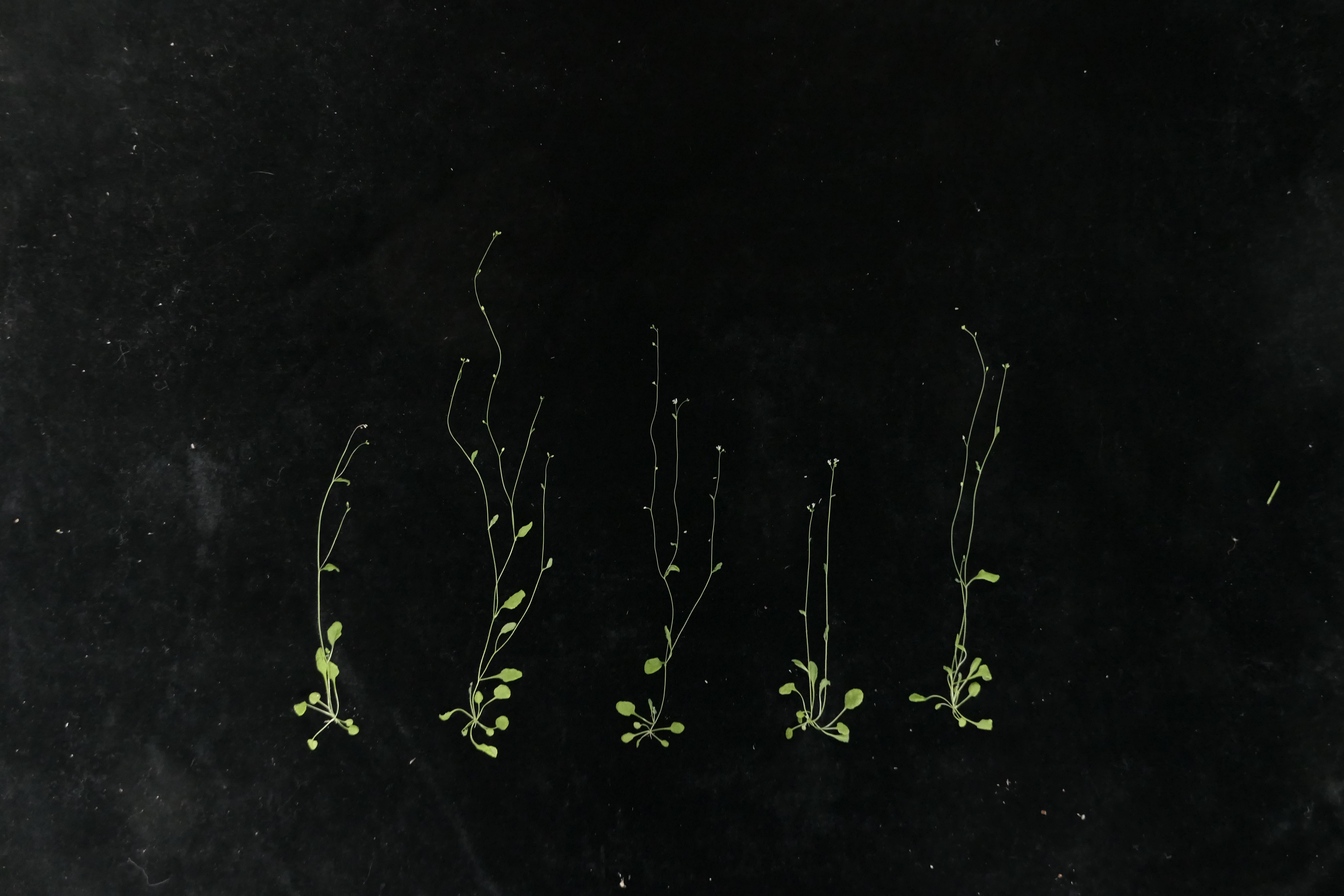

Supplement: Supplementary file 13 — Source Data [file 41467_2024_49721_MOESM13_ESM.zip › 406988_4_data_set_9156724_sddqhm/Source data-Supplementary Dataset/FigS12B.JPG]

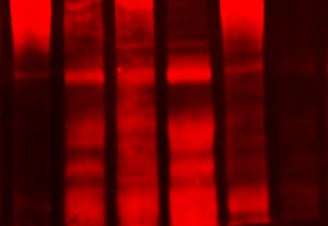

Supplement: Supplementary file 13 — Source Data [file 41467_2024_49721_MOESM13_ESM.zip › 406988_4_data_set_9156724_sddqhm/Source data-Supplementary Dataset/FigS12D-1.tif]

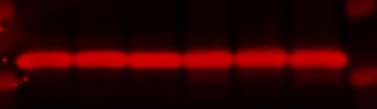

Supplement: Supplementary file 13 — Source Data [file 41467_2024_49721_MOESM13_ESM.zip › 406988_4_data_set_9156724_sddqhm/Source data-Supplementary Dataset/FigS12D-2.jpg]

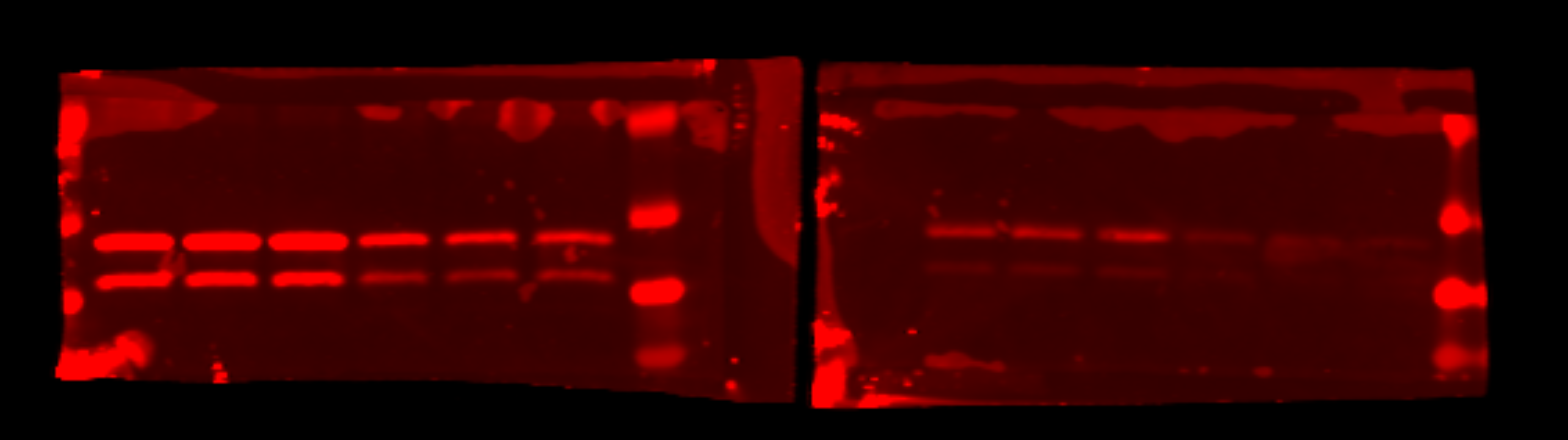

Supplement: Supplementary file 13 — Source Data [file 41467_2024_49721_MOESM13_ESM.zip › 406988_4_data_set_9156724_sddqhm/Source data-Supplementary Dataset/FigS14 H3K9.tif]

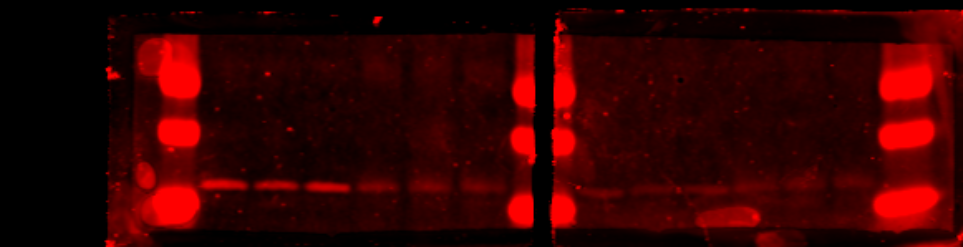

Supplement: Supplementary file 13 — Source Data [file 41467_2024_49721_MOESM13_ESM.zip › 406988_4_data_set_9156724_sddqhm/Source data-Supplementary Dataset/FigS14-1.tif]

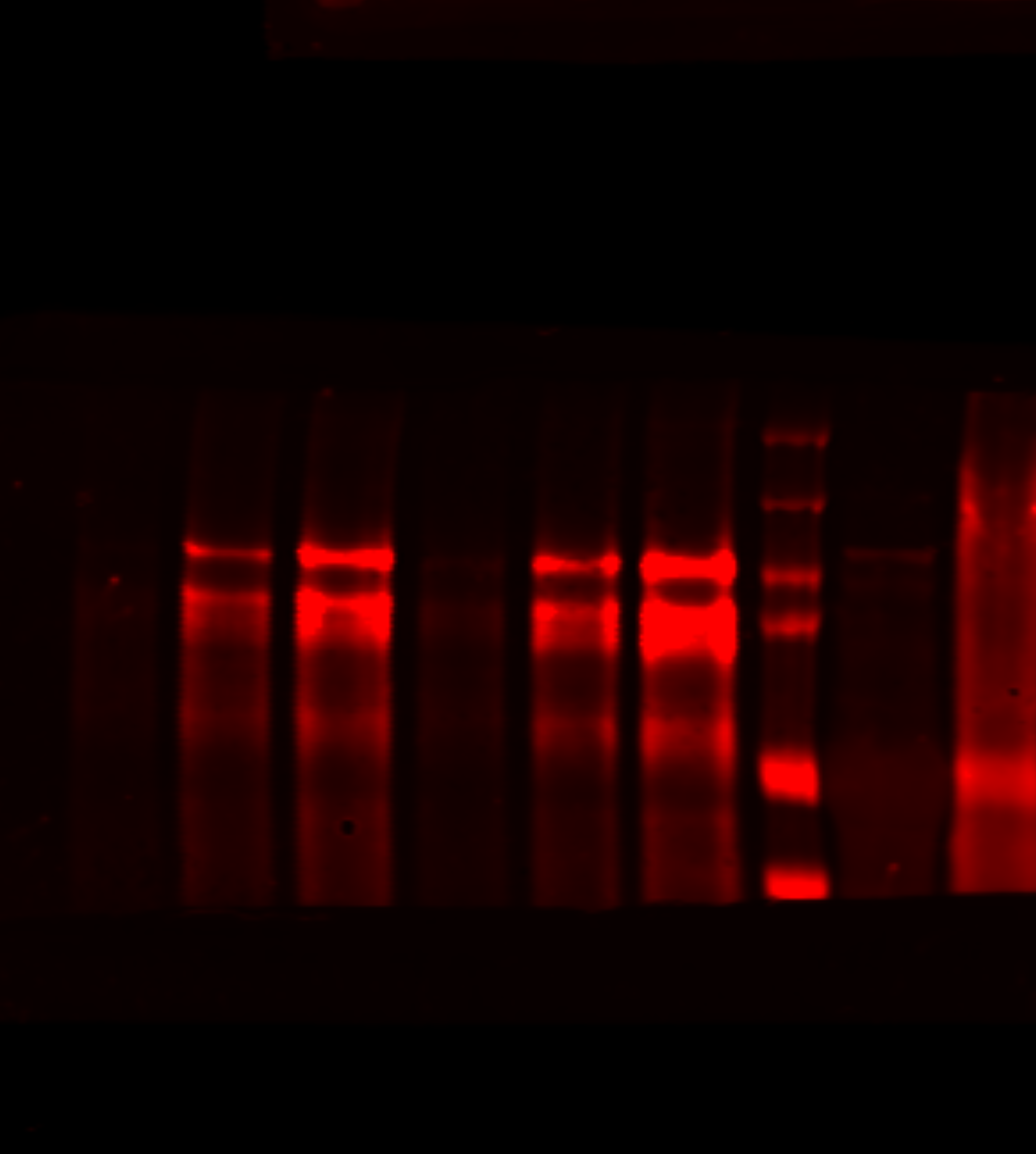

Supplement: Supplementary file 13 — Source Data [file 41467_2024_49721_MOESM13_ESM.zip › 406988_4_data_set_9156724_sddqhm/Source data-Supplementary Dataset/FigS19-1.tif]

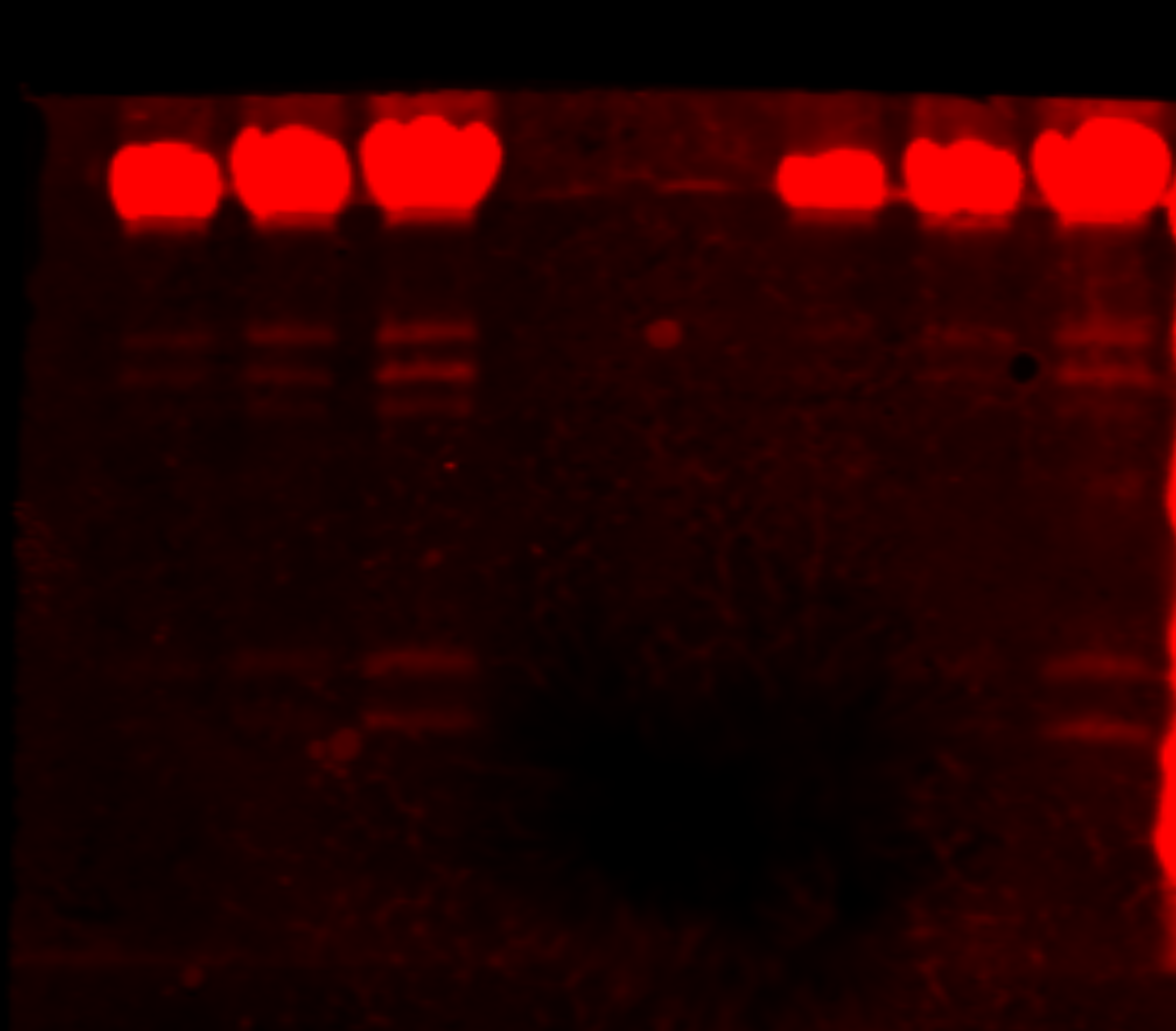

Supplement: Supplementary file 13 — Source Data [file 41467_2024_49721_MOESM13_ESM.zip › 406988_4_data_set_9156724_sddqhm/Source data-Supplementary Dataset/FigS19-2.tif]

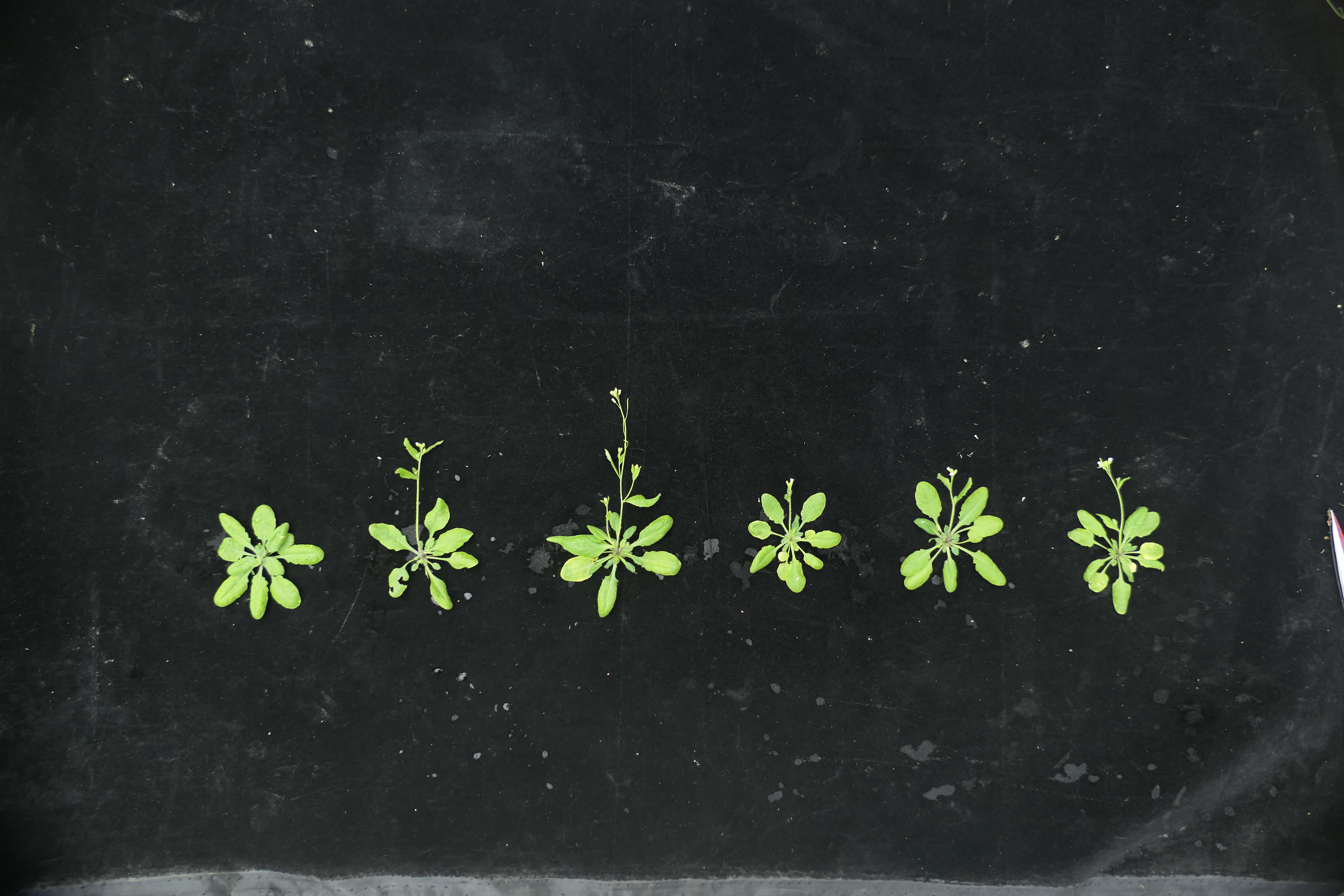

Supplement: Supplementary file 13 — Source Data [file 41467_2024_49721_MOESM13_ESM.zip › 406988_4_data_set_9156724_sddqhm/Source data-Supplementary Dataset/FigS23A.JPG]

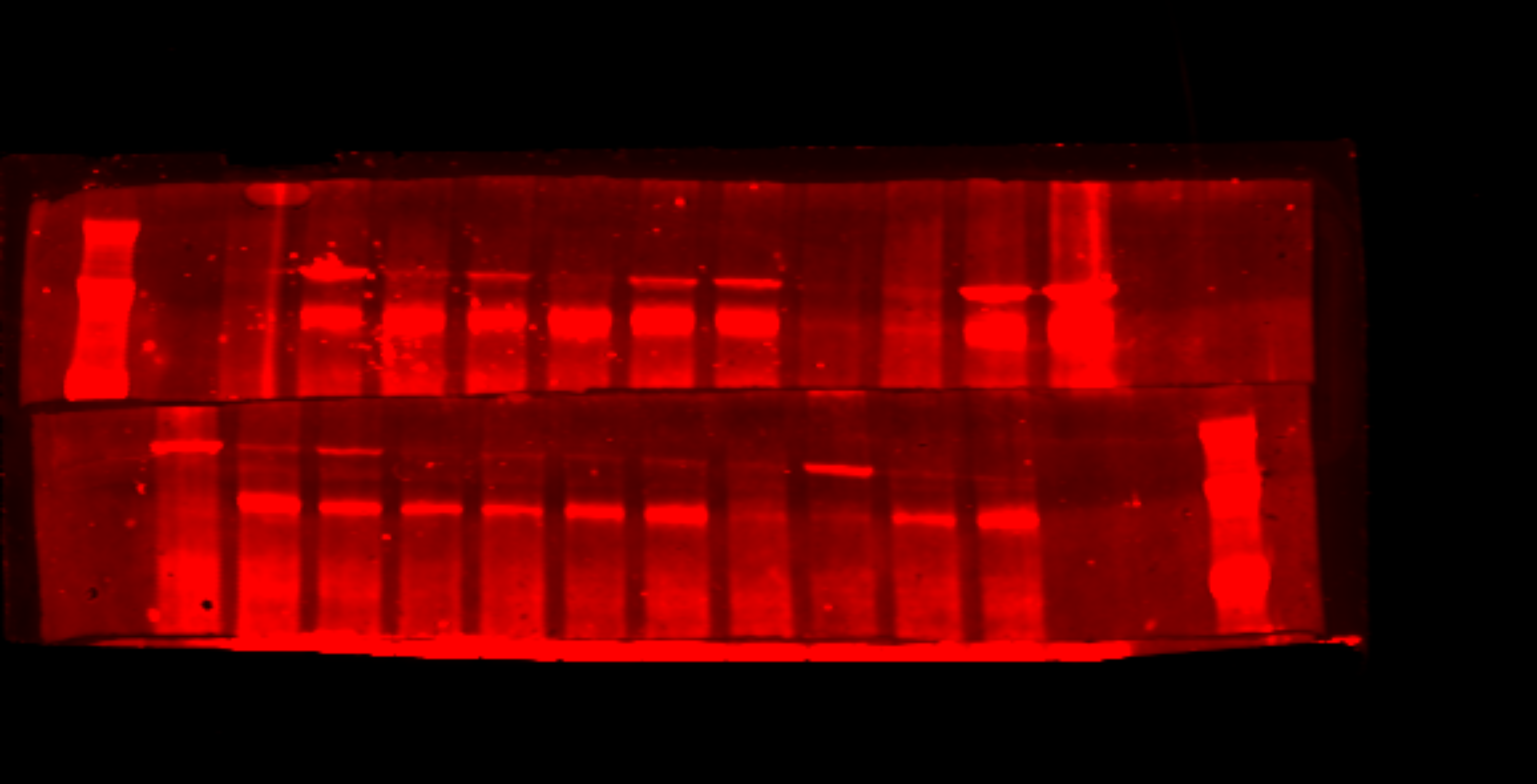

Supplement: Supplementary file 13 — Source Data [file 41467_2024_49721_MOESM13_ESM.zip › 406988_4_data_set_9156724_sddqhm/Source data-Supplementary Dataset/FigS23AB WB.tif]

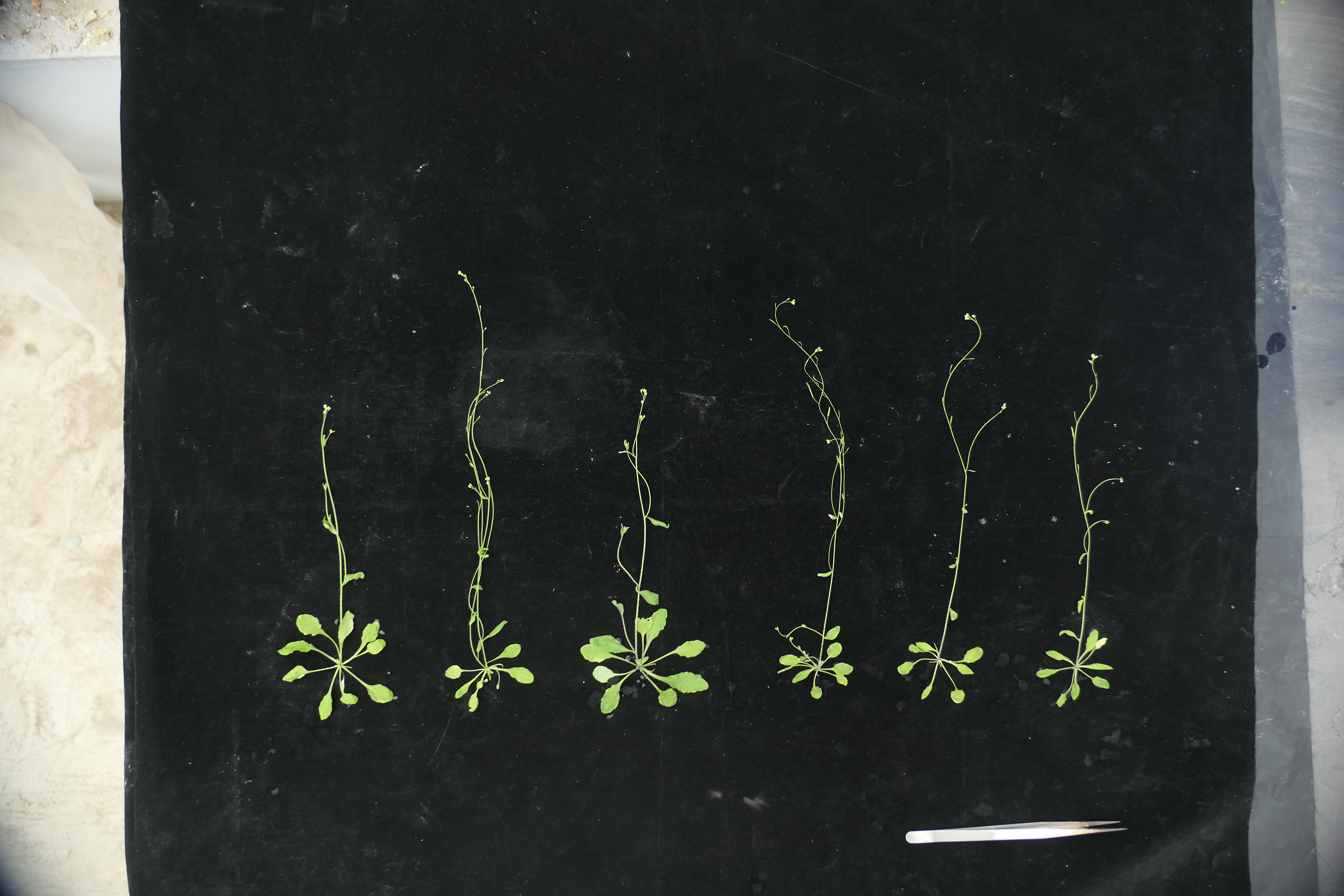

Supplement: Supplementary file 13 — Source Data [file 41467_2024_49721_MOESM13_ESM.zip › 406988_4_data_set_9156724_sddqhm/Source data-Supplementary Dataset/FigS23B.JPG]

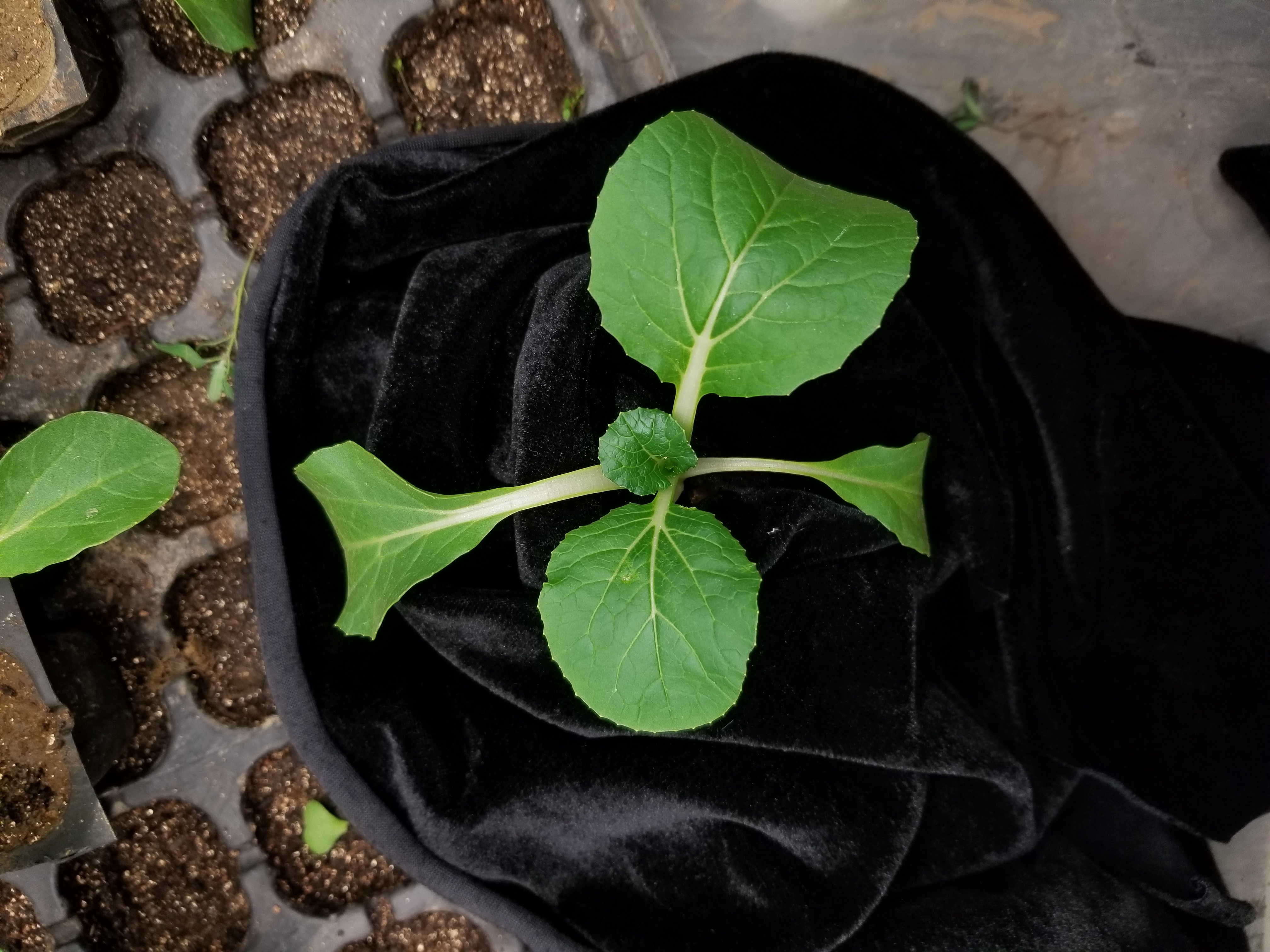

Supplement: Supplementary file 13 — Source Data [file 41467_2024_49721_MOESM13_ESM.zip › 406988_4_data_set_9156724_sddqhm/Source data-Supplementary Dataset/FigS3/20190416_164825.jpg]

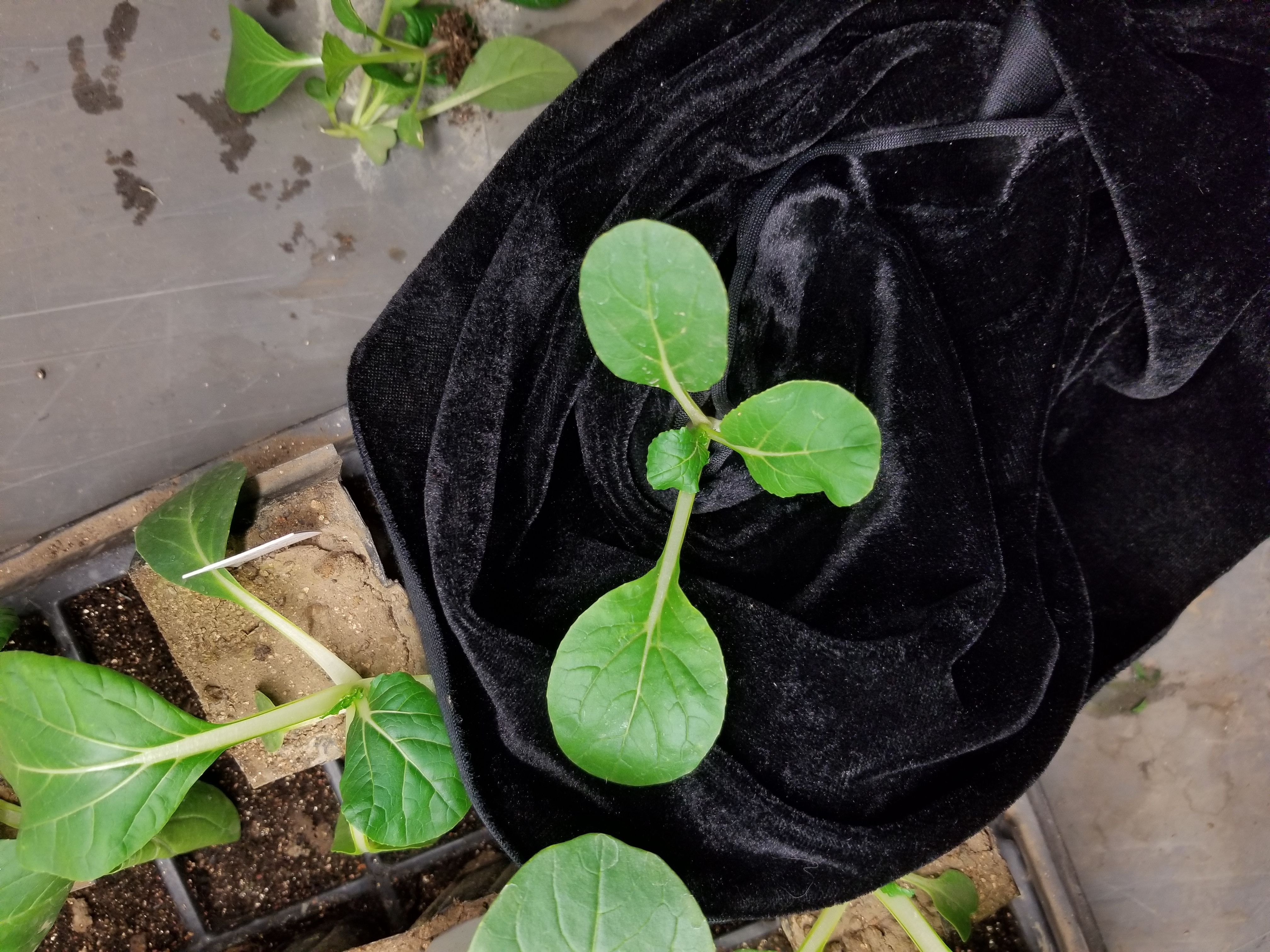

Supplement: Supplementary file 13 — Source Data [file 41467_2024_49721_MOESM13_ESM.zip › 406988_4_data_set_9156724_sddqhm/Source data-Supplementary Dataset/FigS3/20190416_165825.jpg]

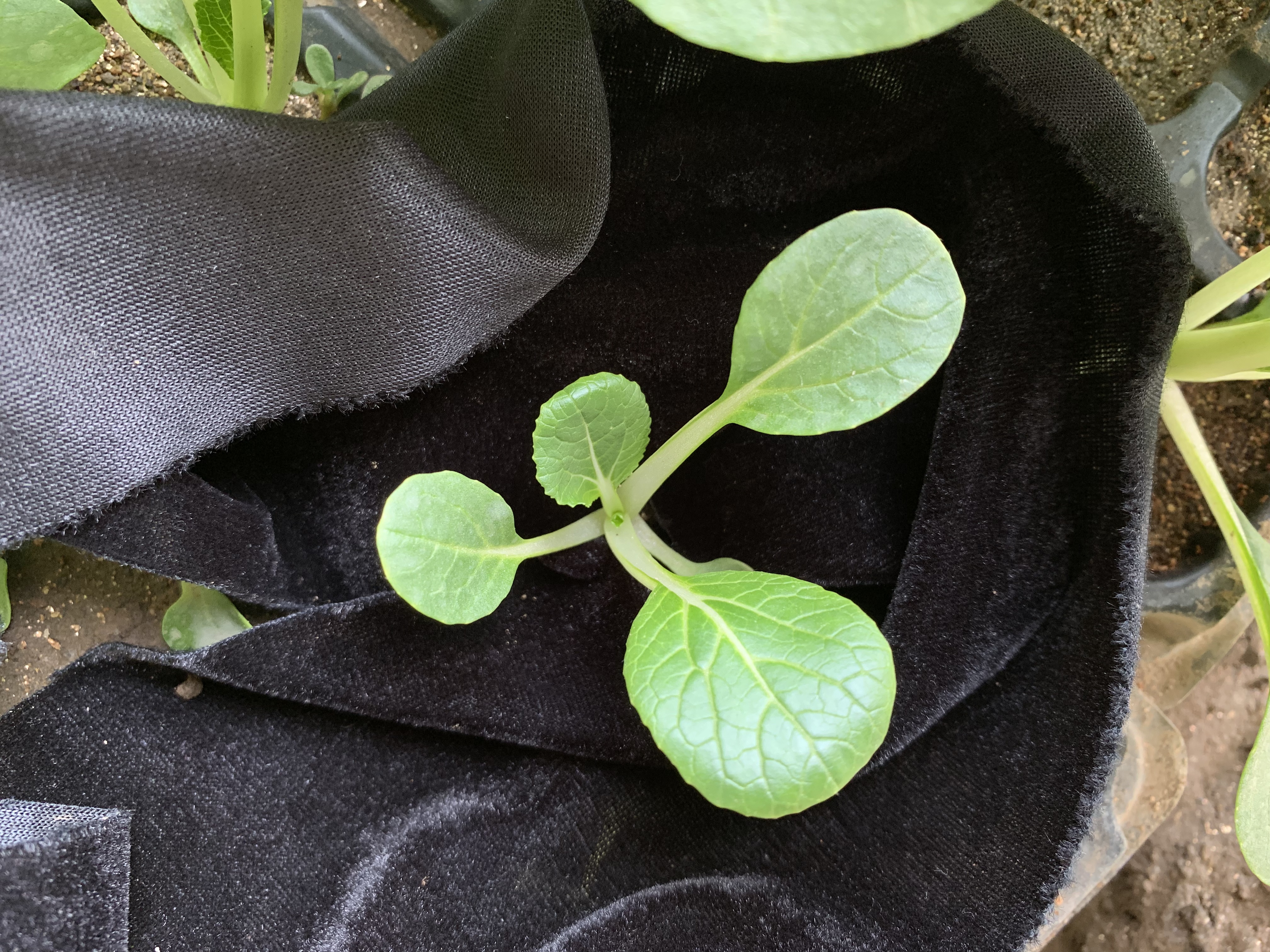

Supplement: Supplementary file 13 — Source Data [file 41467_2024_49721_MOESM13_ESM.zip › 406988_4_data_set_9156724_sddqhm/Source data-Supplementary Dataset/FigS3/IMG_6549.JPG]

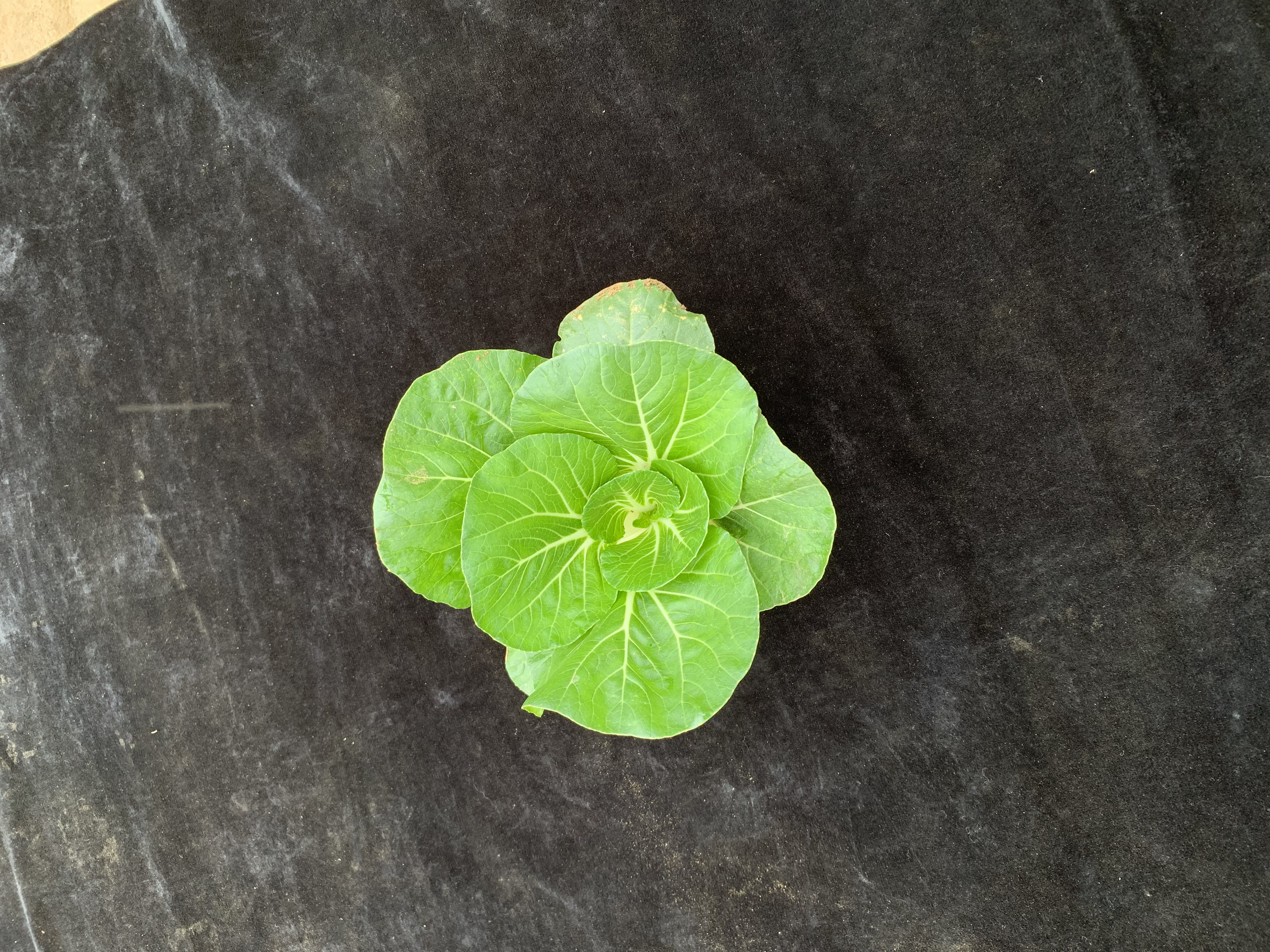

Supplement: Supplementary file 13 — Source Data [file 41467_2024_49721_MOESM13_ESM.zip › 406988_4_data_set_9156724_sddqhm/Source data-Supplementary Dataset/FigS3/IMG_8893.JPG]

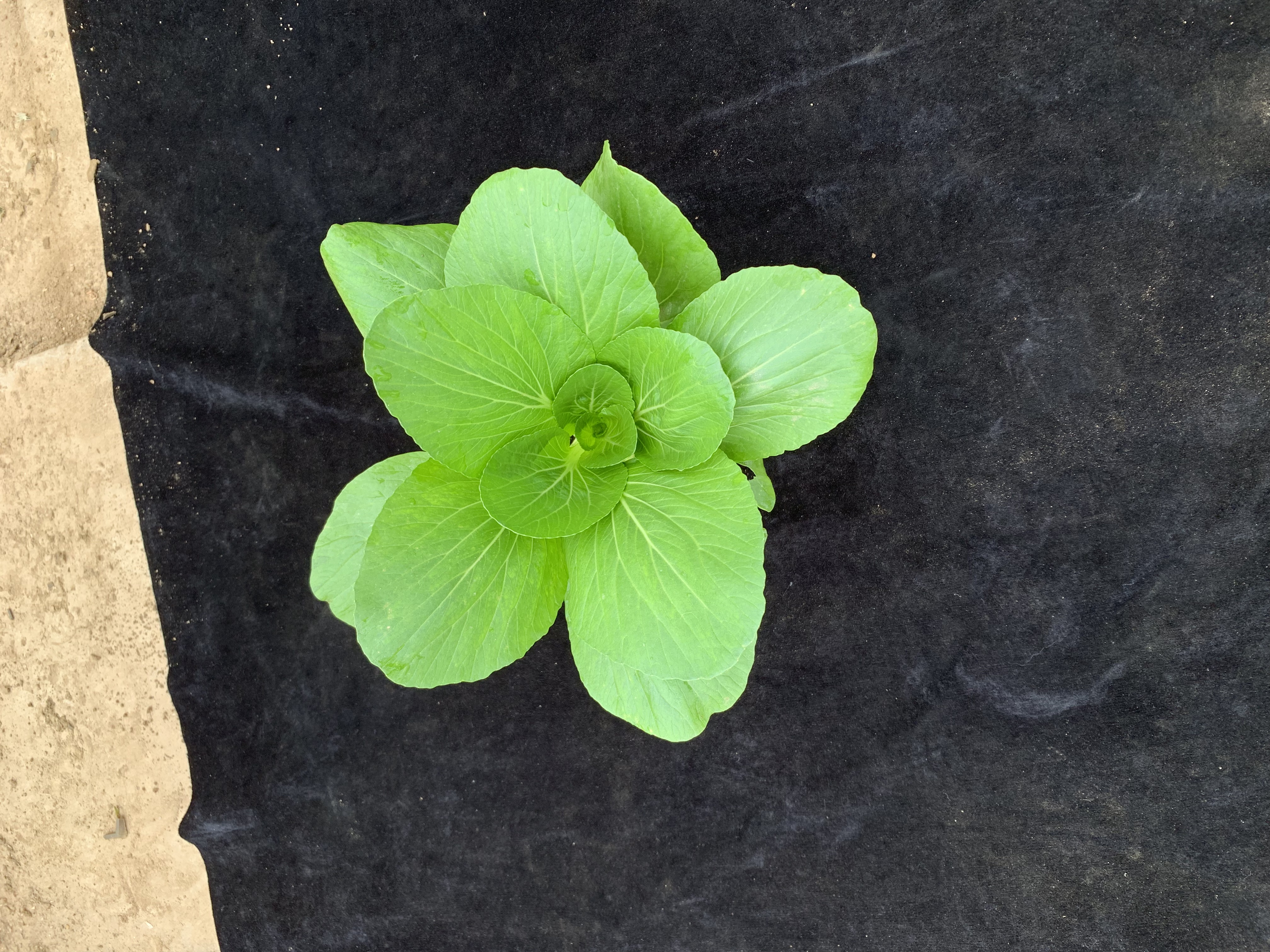

Supplement: Supplementary file 13 — Source Data [file 41467_2024_49721_MOESM13_ESM.zip › 406988_4_data_set_9156724_sddqhm/Source data-Supplementary Dataset/FigS3/IMG_8897.JPG]

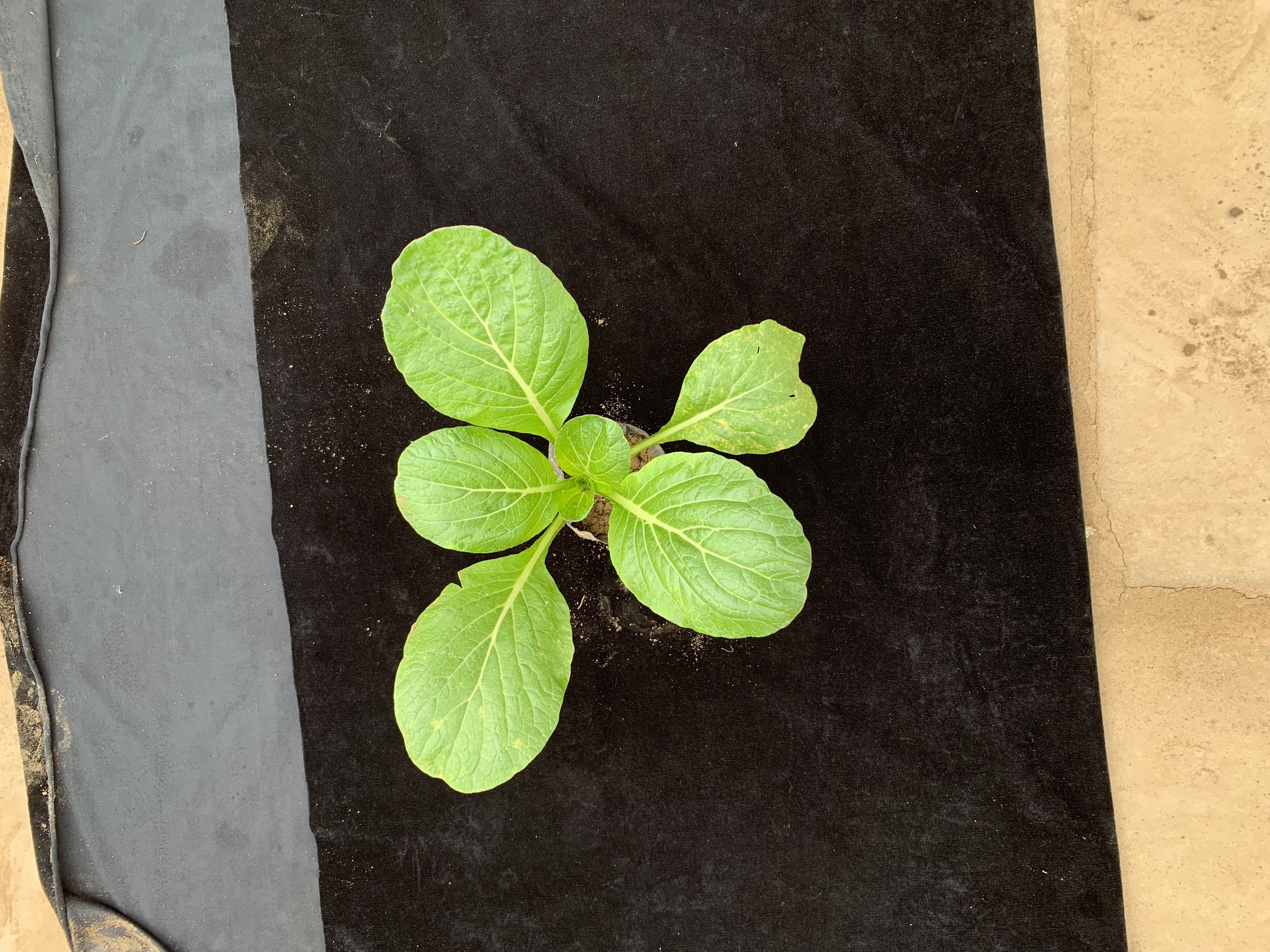

Supplement: Supplementary file 13 — Source Data [file 41467_2024_49721_MOESM13_ESM.zip › 406988_4_data_set_9156724_sddqhm/Source data-Supplementary Dataset/FigS3/IMG_8902.JPG]

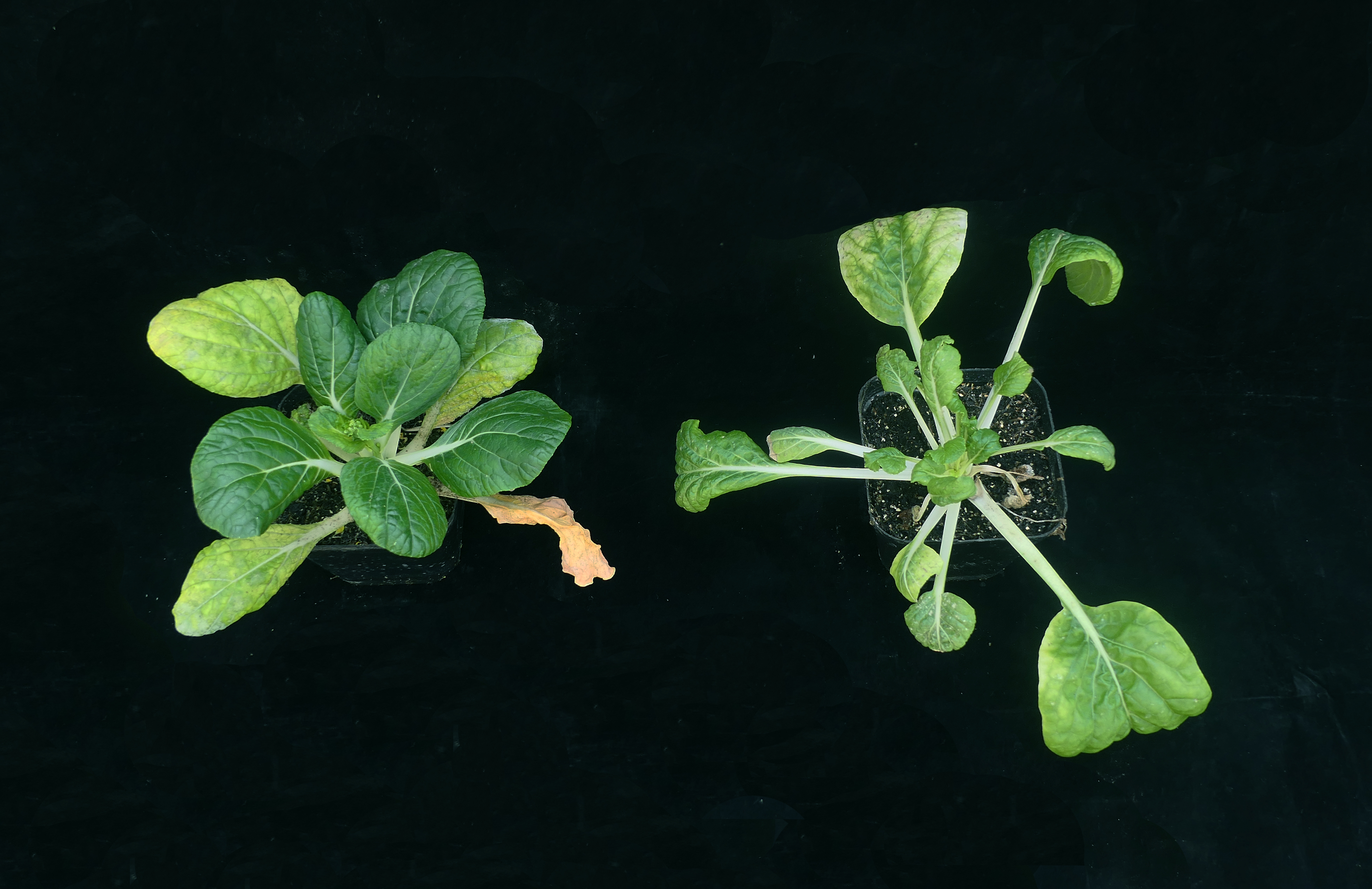

Supplement: Supplementary file 13 — Source Data [file 41467_2024_49721_MOESM13_ESM.zip › 406988_4_data_set_9156724_sddqhm/Source data-Supplementary Dataset/FigS4/FigS4 DG016.jpg]

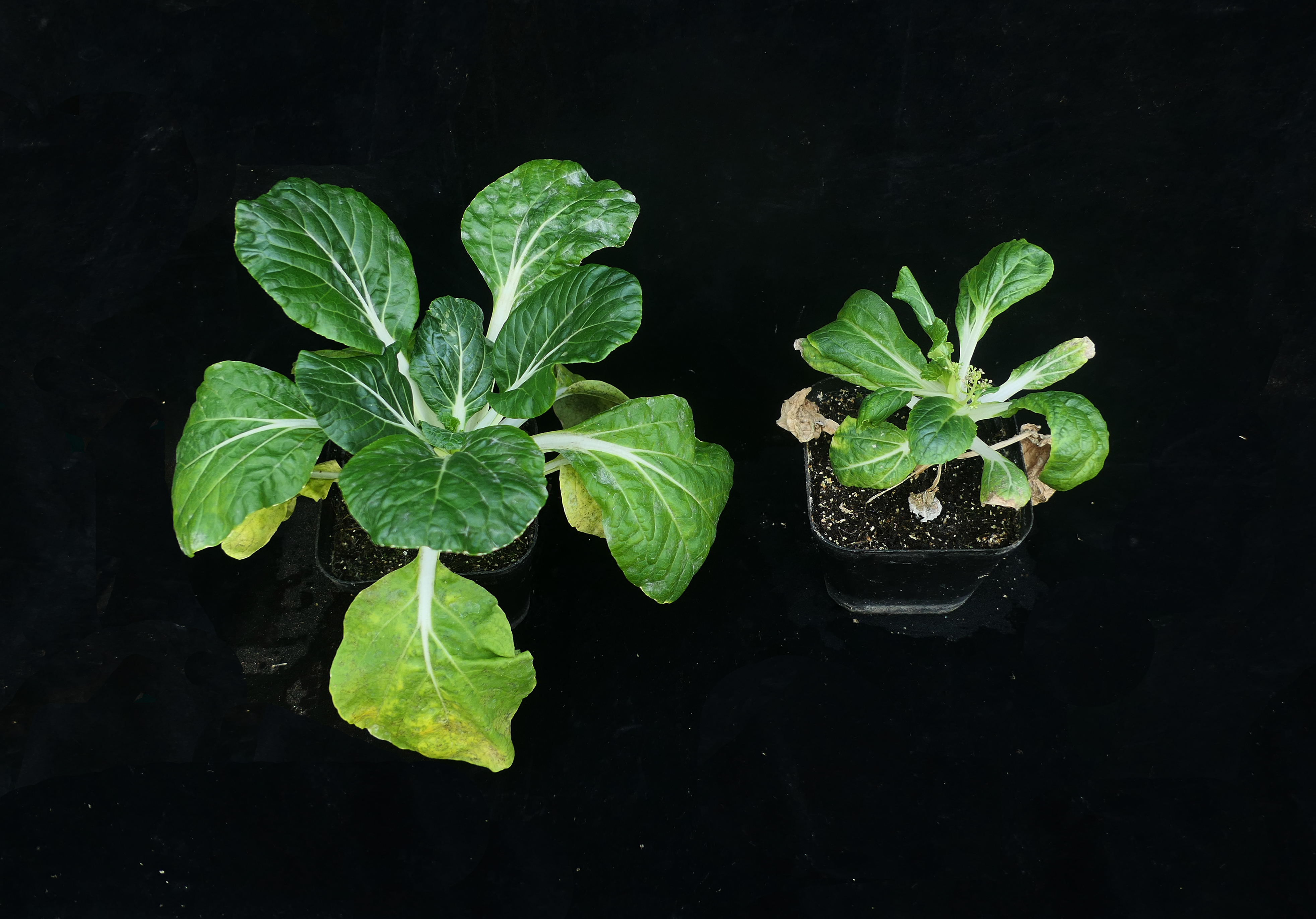

Supplement: Supplementary file 13 — Source Data [file 41467_2024_49721_MOESM13_ESM.zip › 406988_4_data_set_9156724_sddqhm/Source data-Supplementary Dataset/FigS4/FigS4 DG109.jpg]

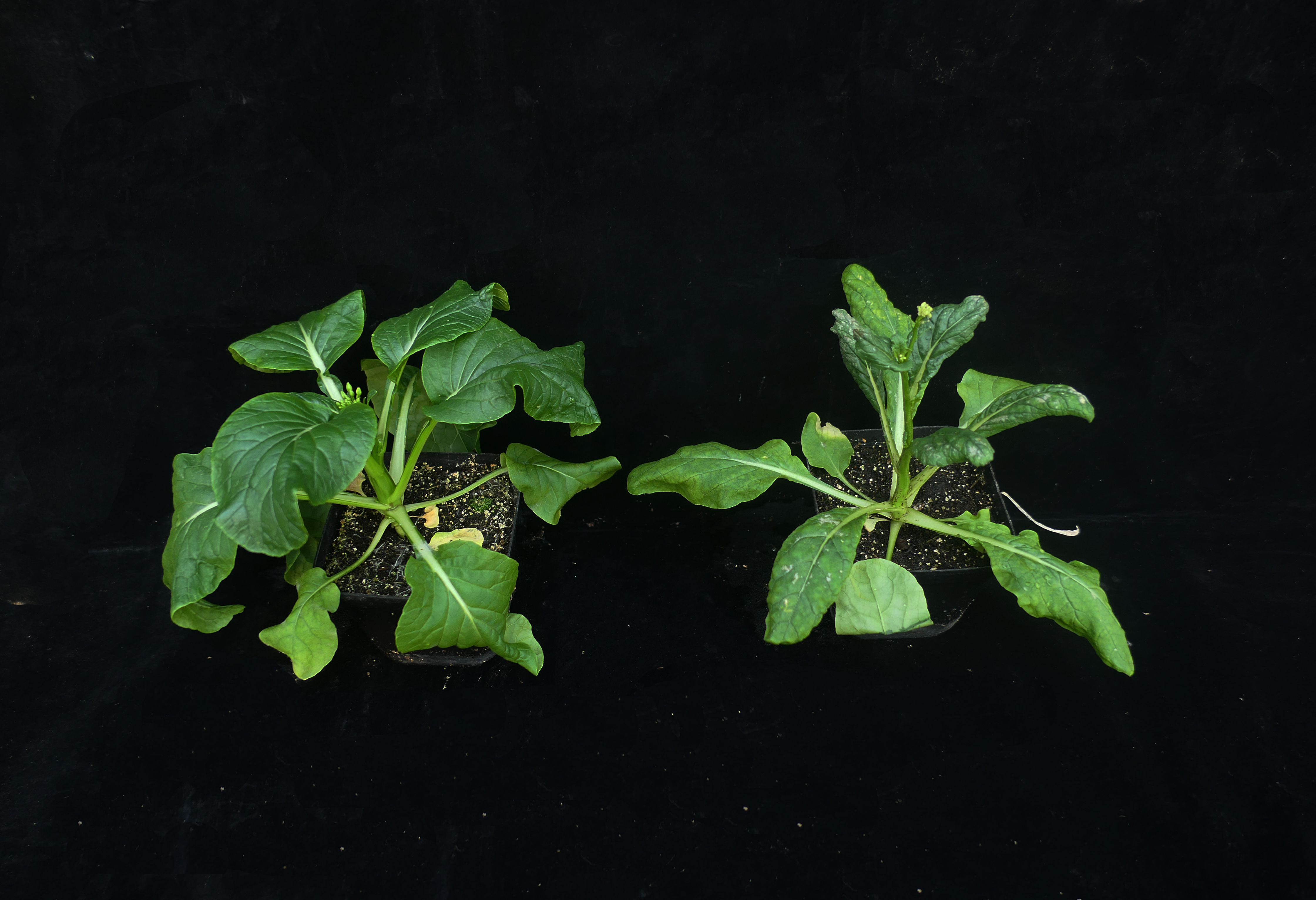

Supplement: Supplementary file 13 — Source Data [file 41467_2024_49721_MOESM13_ESM.zip › 406988_4_data_set_9156724_sddqhm/Source data-Supplementary Dataset/FigS4/FigS4 Par110.jpg]

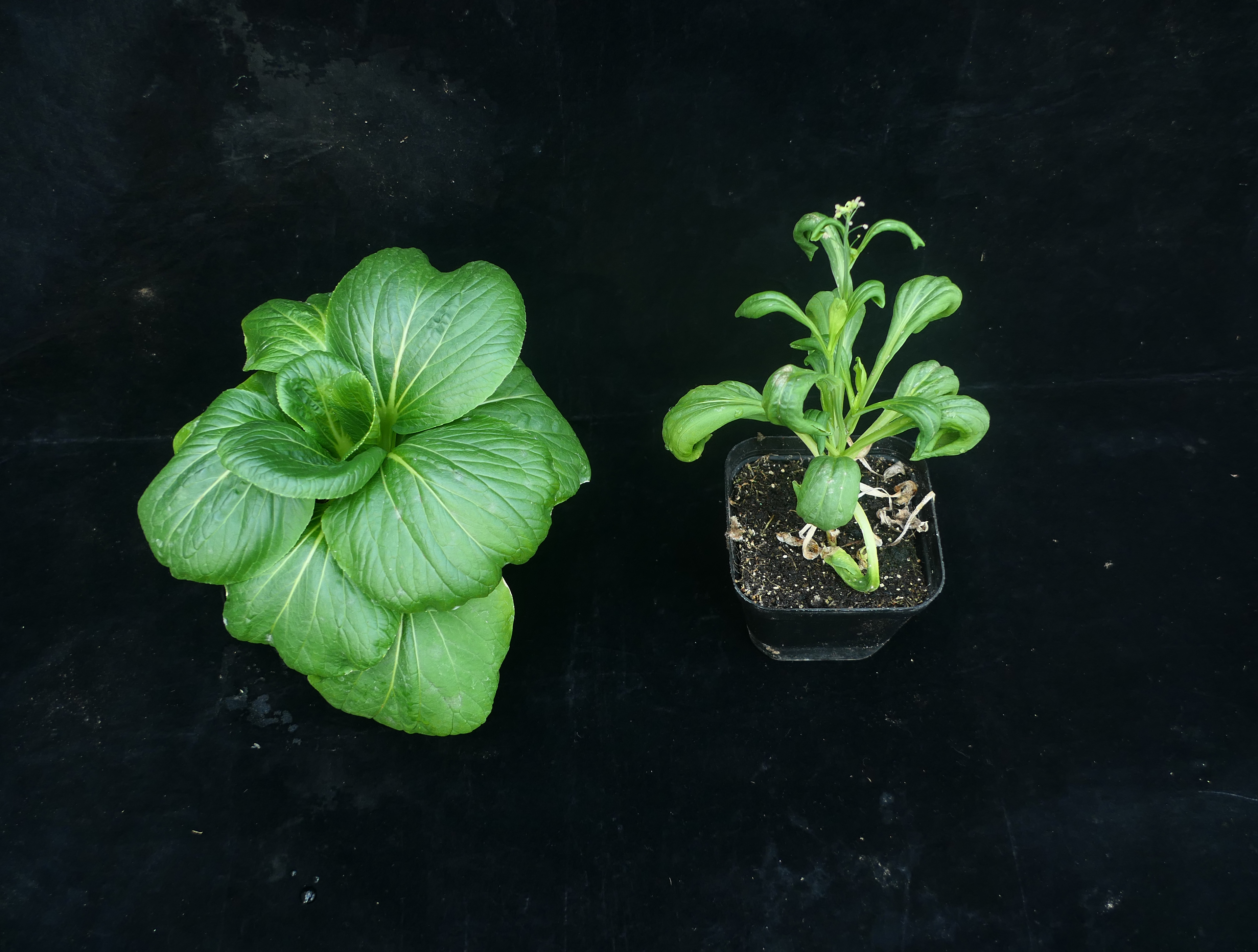

Supplement: Supplementary file 13 — Source Data [file 41467_2024_49721_MOESM13_ESM.zip › 406988_4_data_set_9156724_sddqhm/Source data-Supplementary Dataset/FigS4/FigS4 PC032.jpg]
